# Supplementary figures and images for: Proof of mechanism and target engagement of glutamatergic drugs for the treatment of schizophrenia: RCTs of pomaglumetad and TS-134 on ketamine-induced psychotic symptoms and pharmacoBOLD in healthy volunteers
Source: Neuropsychopharmacology. 2020 May 13;45(11):1842–50. doi: 10.1038/s41386-020-0706-z (PMC7608251; doi:10.1038/s41386-020-0706-z)

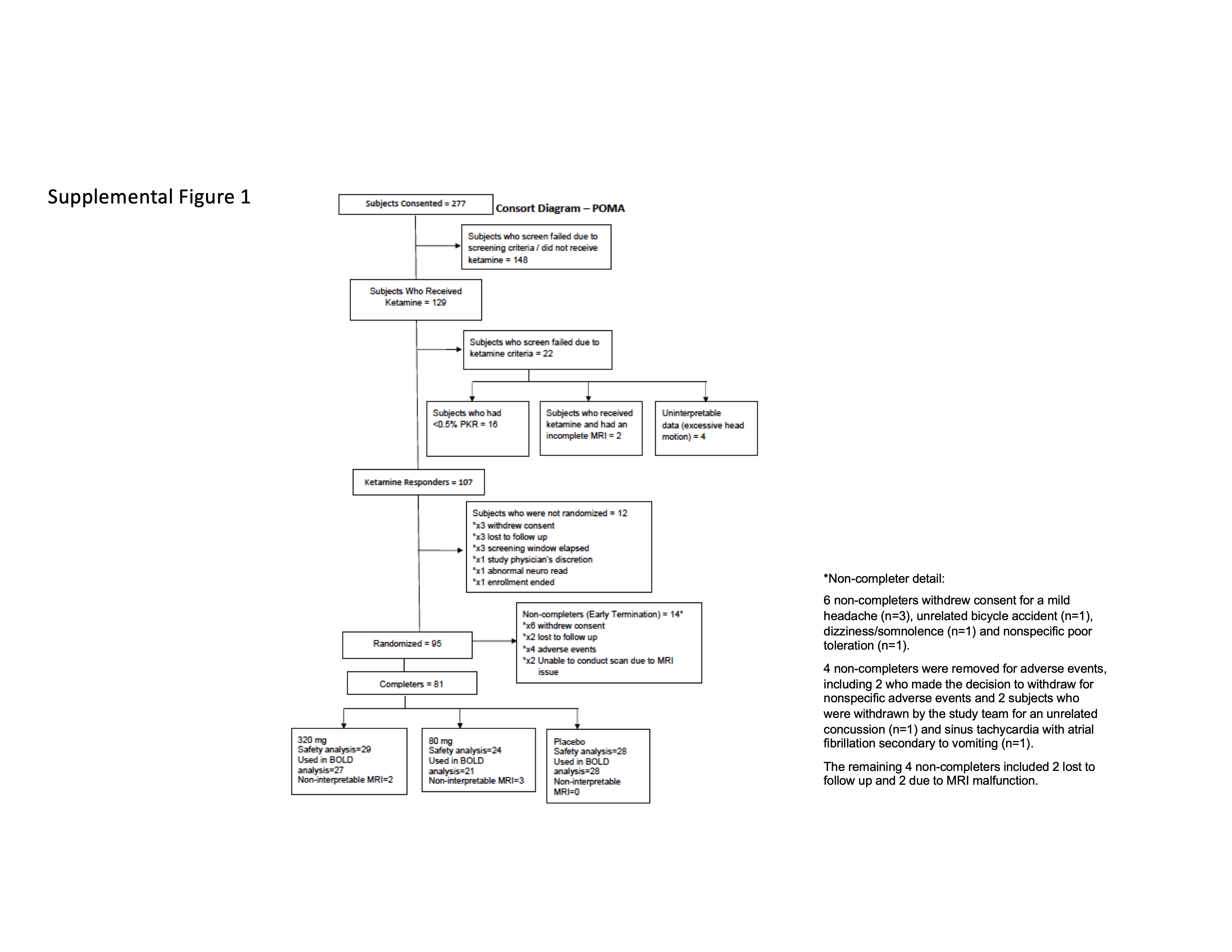

Supplement: Supplementary file 1 — Supplemental Figure 1 [file 41386_2020_706_MOESM1_ESM.tif]

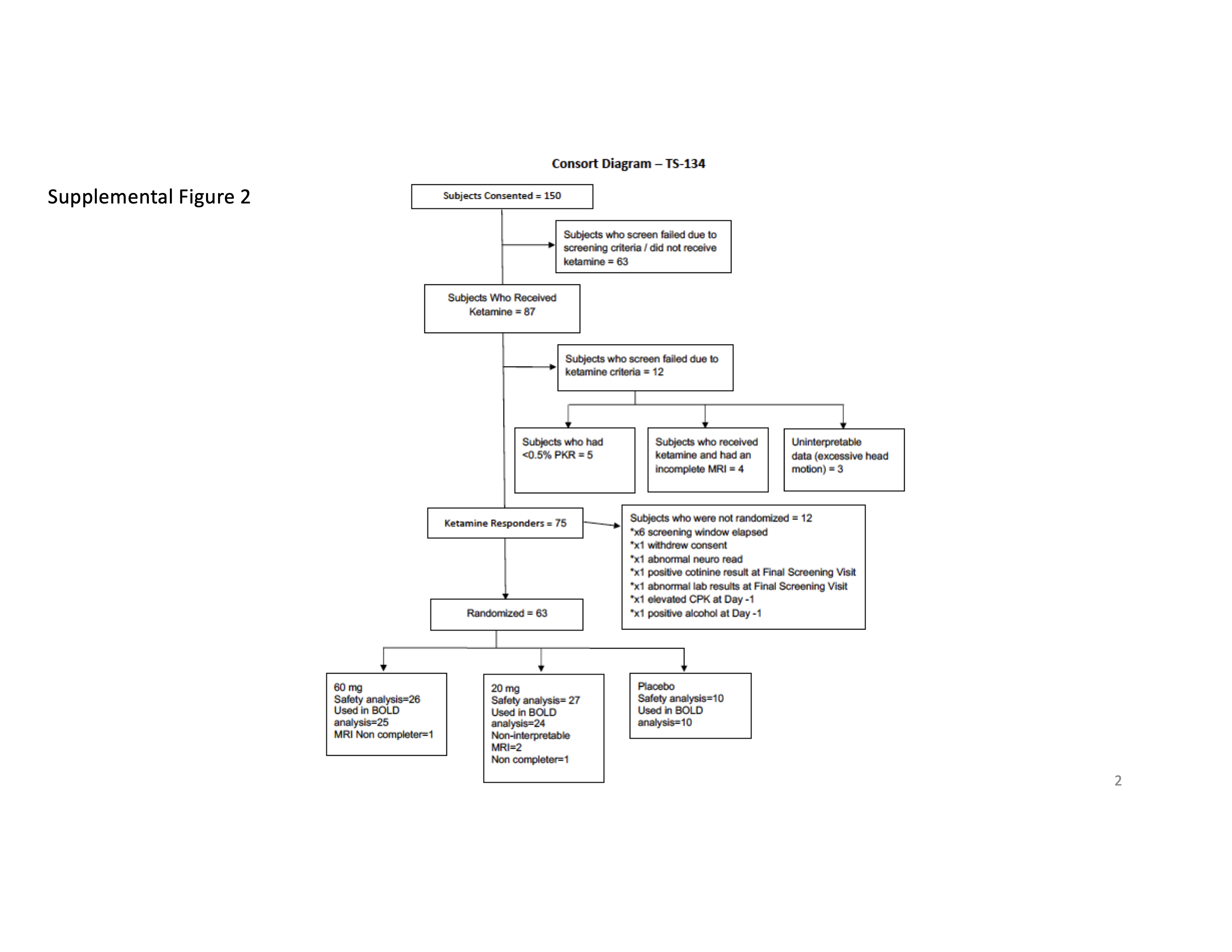

Supplement: Supplementary file 2 — Supplemental Figure 2 [file 41386_2020_706_MOESM2_ESM.tif]

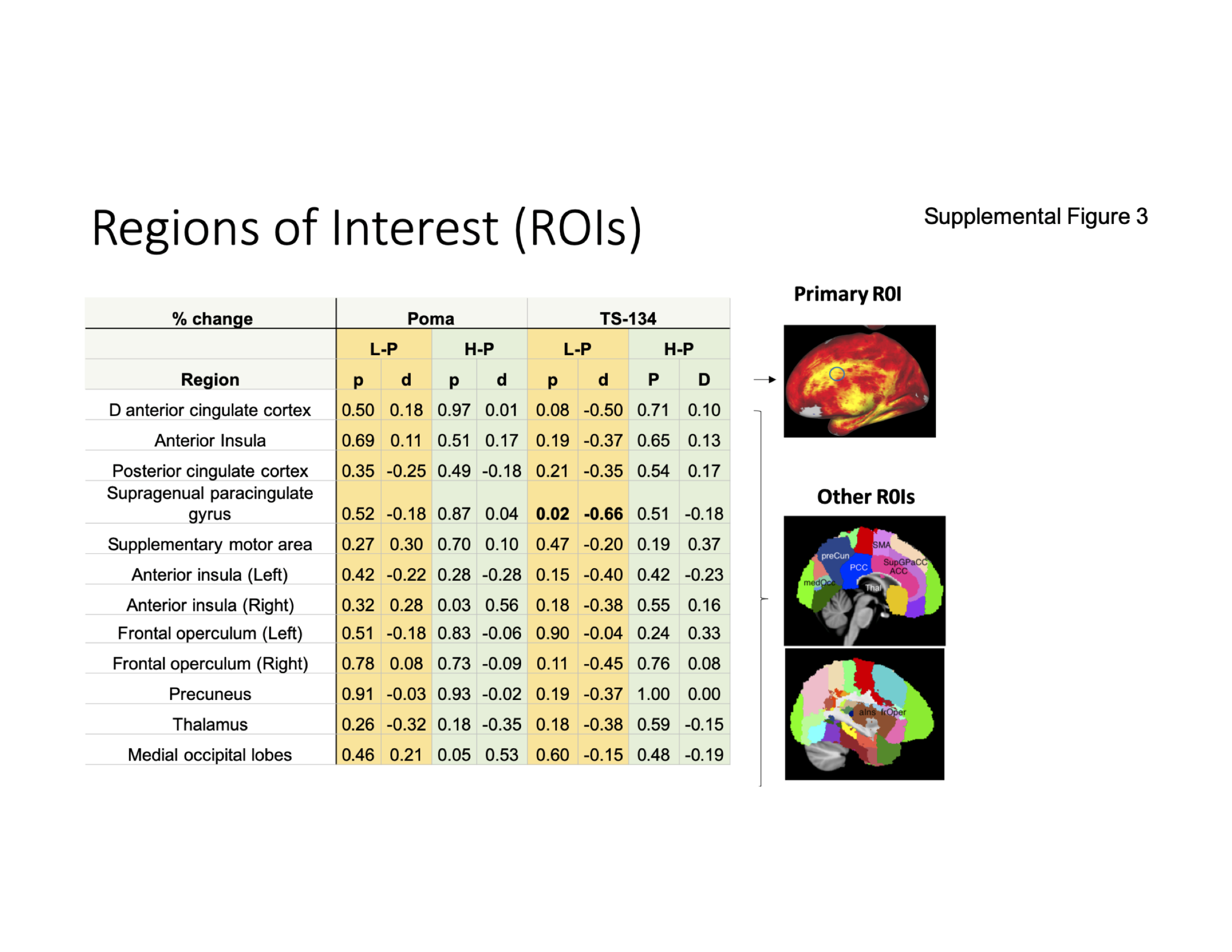

Supplement: Supplementary file 3 — Supplemental Figure 3 [file 41386_2020_706_MOESM3_ESM.tif]

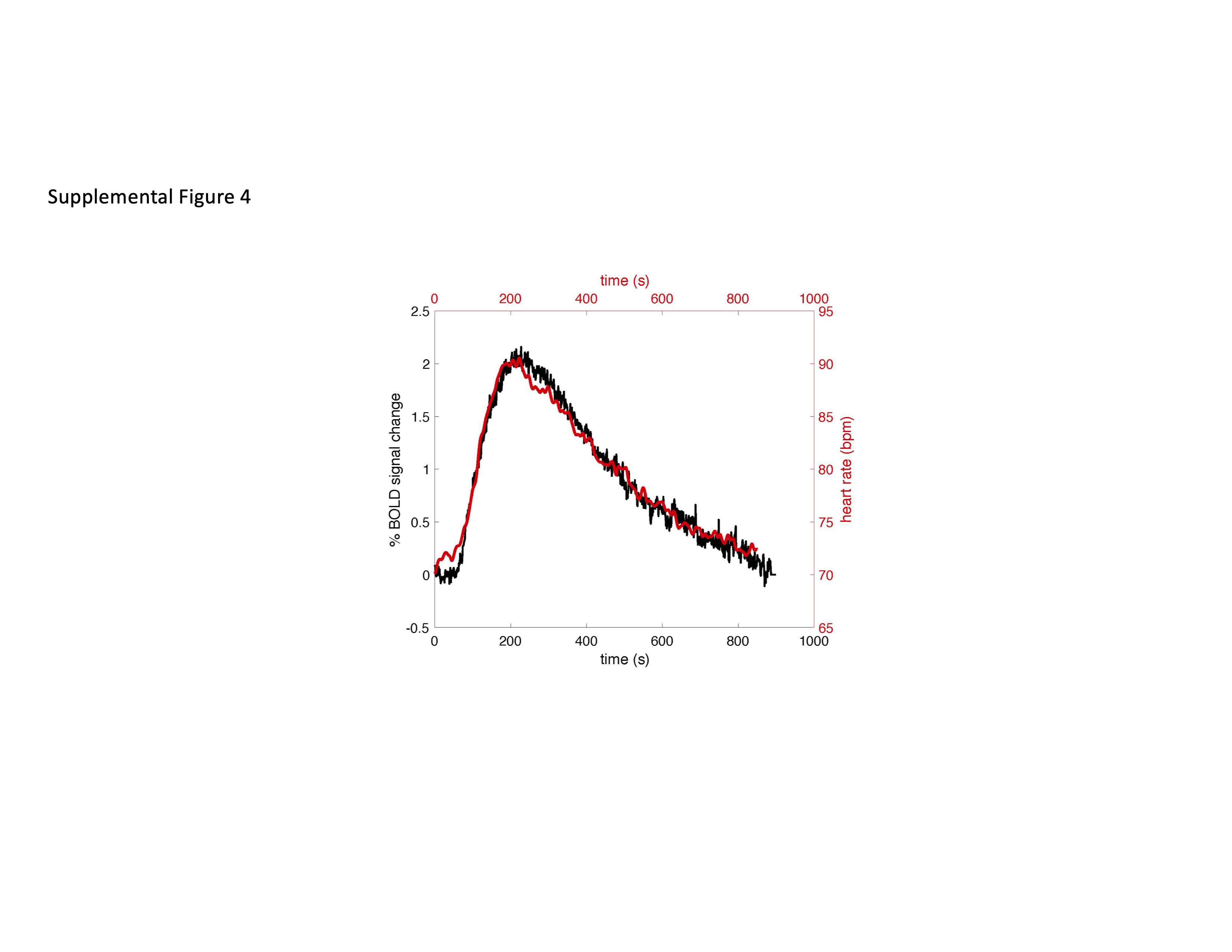

Supplement: Supplementary file 4 — Supplemental Figure 4 [file 41386_2020_706_MOESM4_ESM.tif]

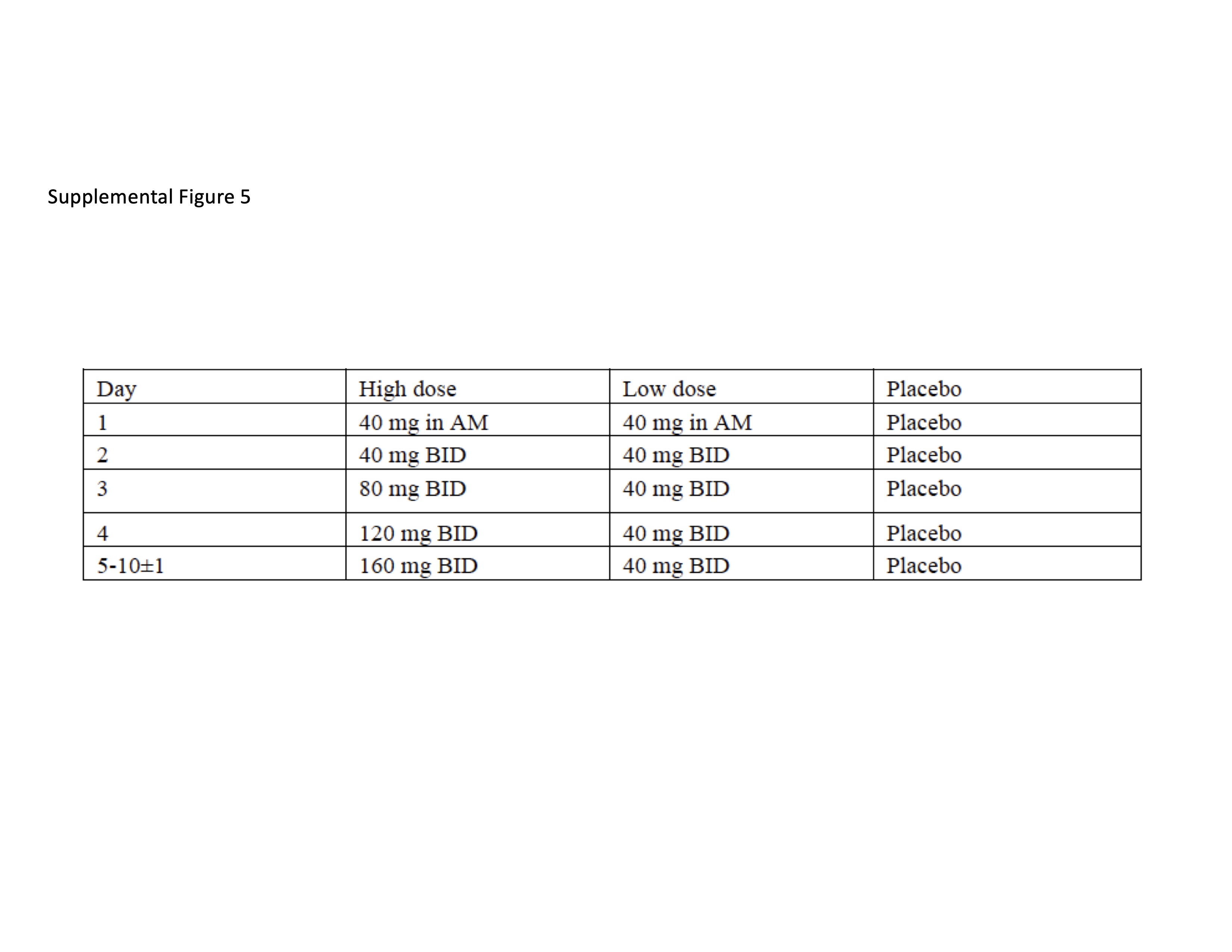

Supplement: Supplementary file 5 — Supplemental Figure 5 [file 41386_2020_706_MOESM5_ESM.tif]

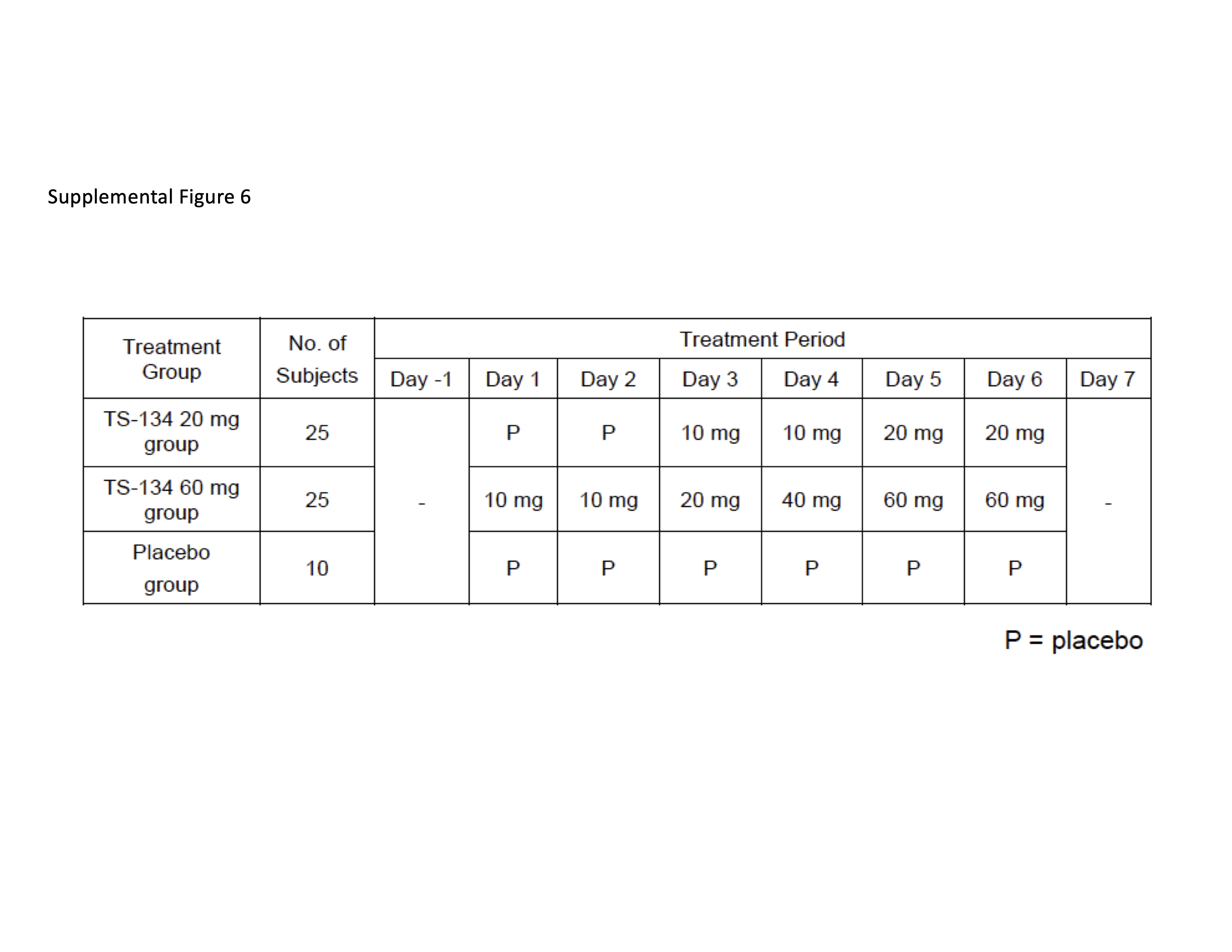

Supplement: Supplementary file 6 — Supplemental Figure 6 [file 41386_2020_706_MOESM6_ESM.tif]
