# Supplementary material for: Proof of mechanism and target engagement of glutamatergic drugs for the treatment of schizophrenia: RCTs of pomaglumetad and TS-134 on ketamine-induced psychotic symptoms and pharmacoBOLD in healthy volunteers
Source: Neuropsychopharmacology. 2020 May 13;45(11):1842–50. doi: 10.1038/s41386-020-0706-z (PMC7608251; doi:10.1038/s41386-020-0706-z)
Supplement: Supplementary file 7 — Supplemental Tables [file 41386_2020_706_MOESM7_ESM.pdf]

		Within Group Screening-Day 10 Change									Between Group			
		Placebo			Low Dose			High dose			L-P		H-P	
		Value	P	d	Value	p	d	Value	P	d	P	D	P	d
**dACC**	**%change (peak)**	-0.1±0.6	0.39	-0.15	0.0±0.8	0.89	0.03	-0.1±0.5	0.42	-0.14	0.5	0.18	0.97	0.01
**Symptoms**	**BPRS Total**	-0.1±5.4	0.97	0.01	-0.9±4.5	0.23	-0.19	-2.1±3.9	**<0.01**	**-0.41**	0.35	-0.21	**0.04**	**-0.44**
	**BPRS Positive**	0.3±2.3	0.41	0.04	-0.6±2.9	0.30	-0.2	-0.4±2.3	0.14	-0.25	0.19	-0.35	*0.10*	*-0.41*
	**BPRS Negative**	0.43±1.8	**0.03**	**0.66**	-0.2±0.5	0.29	-0.36	-0.1±0.9	0.65	-0.14	**0.03**	**-1.1**	*0.07*	*0.83*
	**CADSS Total**	-8.0±13.5	**<0.001**	**-0.41**	–5.0±9.7	**0.03**	**-0.28**	-4.8±12.2	**<0.001**	**-0.48**	0.49	0.13	0.72	-0.06

Supplemental Table 1: Observed mean changes in BOLD and Symptoms (POMA)

Abbreviations: dACC: dorsal anterior cingulate cortex; BPRS: Brief Psychiatric Rating Scale; CADSS: Clinician Administered Dissociative States Scale L=Low dose; P=placebo; H=high dose; p<0.05 in bold and p<0.1 in italics

Supplemental Table 2: Observed mean changes in BOLD and Symptoms (TS-134)

		**Within Group Screening-Day 6 Change**									**Between Group**			
		**Placebo**			**Low Dose**			**High dose**			**L-P**		**H-P**	
		**Value**	**P**	**d**	**Value**	**p**	**d**	**Value**	**P**	**d**	**P**	**D**	**P**	**d**
**DACC**	**%change (peak)**	-0.1±0.3	0.98	-0.01	-0.3±0.3	**0.004**	**-0.56**	-0.1±0.5	0.67	-0.08	0.12	-0.57	0.84	-0.07
**Symptoms**	**BPRS Total**	0.5±2.4	0.93	0.12	-1.2±3.6	*0.06*	*-0.32*	-0.2±3.4	0.92	-0.04	0.28	-0.47	0.89	-0.19
	**BPRS Positive**	0.1±1.5	0.83	0.04	-0.7±1.5	**0.02**	**-0.36**	-0.2±2.3	0.41	-0.12	0.14	-0.42	0.53	-0.17
	**BPRS Negative**	0.1±0.6	0.89	0.03	0.2±1.7	0.45	0.11	0.2±0.9	0.34	0.14	0.77	0.08	0.69	0.11
	**CADSS Total**	0.7±7.3	0.89	0.06	–2.4±8.3	0.18	-0.23	-1.0±11.1	0.68	-0.10	0.40	-0.31	0.74	-0.18

Abbreviations: dACC: dorsal anterior cingulate cortex; AI: anterior insula; BPRS: Brief Psychiatric Rating Scale; CADSS: Clinician Administered Dissociative States Scale L=Low dose; P=placebo; H=high dose; p<0.05 in bold and p<0.1 in italics

Supplemental Table 3[1]:

Cluster Index	# of Voxels	MAX	X	Y	Z	
1	249	2.6	-56	10	-22	left temporal pole
2	193	2.39	-12	24	14	left caudate
3	126	2.16	-36	36	36	right posterior cingulate/precuneus
4	113	2.17	18	-82	40	right lateral occipital cortex
5	109	2.27	2	30	30	right mid cingulate cortex
6	106	2.11	-6	-22	30	left mid cingulate cortex
7	84	1.88	-14	-20	40	left precentral gyrus
8	81	2.3	10	-44	50	right precuneus cortex
9	68	2.18	10	10	-14	ventral striatum
10	58	1.92	-6	36	32	left paracingulate gyrus
11	50	1.96	4	46	30	right paracingulate gyrus
12	46	1.93	10	-18	38	right mid cingulate cortex

1. Significant cluster in clusters in voxelwise, whole brain analysis ($p<0.05$). Coordinates listed in Z scores.

Supplemental Table 4: Drug and Ketamine levels

Drug (ng/ml)	Pomaglumetad			TS-134		
	Placebo	Low dose	High dose	Placebo	Low dose	High dose
Ketamine (screening)	65.9±45.7	69.4±23.0	74.6±48.2	69.9± 25.8	62.0±15.1	74.0±27.6
Ketamine (final)	53.6±15.4	59.9±16.8	58.6±34.9	57.3±15.8	58.8±19.2	56.1±16.5
Ketamine Difference (screening-final)	-11.7±49.0	-9.5±21.9	-16.0±59.6	-12.6±34.0	-3.2±18.82	-18.5±21.8
Day 1 LY2140023		87.8±60.3	72.1±60.3			
Day 5 LY2140023		50.1±52.2	319.8±214.0			
Day 10 - Pre-Infusion LY2140023		3.3±5.0	53.8±154.0			
Day 10 - Post-Infusion LY2140023		83.2±52.8	412.7±273.9			
Day 1 LY404039		114.6±68.2	88.1±75.5			
Day 5 LY404039		94.3±101.6	595.1±396.6			
Day 10 - Pre-Infusion LY404039		14.1±25.3	135.1±245.5			
Day 10 - Post-Infusion LY404039		211.1±108.5	828.9±465.4			
Day 6 TS-134 (MGS0008)					72.3±30.3	235.0±68.2

Ketamine levels were measured 5 minutes after the MRI scanning ended. Active POMA levels (LY404039) and prodrug (LY2140023) levels were measured on day 1 (one-hour post dose), Day 5 (random) and pre/post Day 10 pharmacoBOLD scan. TS-134 levels were measured on day 6, 3 hours post dose.
