# Supplementary material for: Proof of mechanism and target engagement of glutamatergic drugs for the treatment of schizophrenia: RCTs of pomaglumetad and TS-134 on ketamine-induced psychotic symptoms and pharmacoBOLD in healthy volunteers
Source: Neuropsychopharmacology. 2020 May 13;45(11):1842–50. doi: 10.1038/s41386-020-0706-z (PMC7608251; doi:10.1038/s41386-020-0706-z)
Supplement: Supplementary file 8 — Supplemental methods [file 41386_2020_706_MOESM8_ESM.docx]

PharmacoBOLD methods: All data was acquired on a 3T GE MR750 scanner using a 32-channel head coil. T2*-weighted EPI scans were collected for 15 min prior to and during each ketamine infusion. Each fMRI acquisition consisted of 900 volumes (TR = 1 sec, TE = 20 msec, flip angle = 77º, FOV=24cm, resolution of 64x64x23 and voxel size of 3.75 x 3.75 x 5.0 mm). Each functional scan was rigid-body motion corrected, slice time corrected, spatially smoothed (FWHM = 5 mm), and high-pass filtered (1200 sec) using FSL (FMRIB Software Library). Linear regression was performed using 8 motion parameters including 3 translations, 3 rotations, the mean relative motion, and the mean absolute motion. The model residual was then used for quantifying all ketamine-evoked responses

Linear registration to the subject’s high-resolution Spoiled Gradient (SPGR) image (6 df) and to the MNI152 standard space template (12 df) was performed to generate spatial transforms. A region of interest (ROI) analysis was performed using the dorsal anterior cingulate cortex. The ROI mask was created by manually drawing a mask around the peak ketamine response in the frontal cortex from Javitt et al (2018). The center of the mask was MNI: 0, 28,32 extending 32mm anteriorly and 26mm posteriorly. The mask was then transformed to the individual subject’s functional space and multiplied by a gray matter mask to minimize the contribution of non-gray voxels. For each subject, the BOLD time series was extracted from the ROI and fit to a four-parameter gamma model:

$$y\left( t \right) = {a\left( \frac{t_{k\ldots n}}{t_{max}} \right)}^{b\cdot t_{max}}e^{\left( t_{max}-t \right)\cdot b}$$

where *t_max_* is time of maximum response, *a* is amplitude of the response, *b* is the shape parameter of the gamma function, and *t* = *k* to *n* seconds where *k* is the response onset time and *n* is the total acquisition time of 900 seconds.
