# Supplementary material for: Proof of mechanism and target engagement of glutamatergic drugs for the treatment of schizophrenia: RCTs of pomaglumetad and TS-134 on ketamine-induced psychotic symptoms and pharmacoBOLD in healthy volunteers
Source: Neuropsychopharmacology. 2020 May 13;45(11):1842–50. doi: 10.1038/s41386-020-0706-z (PMC7608251; doi:10.1038/s41386-020-0706-z)
Supplement: Supplementary file 9 — POMA protocol [file 41386_2020_706_MOESM9_ESM.docx]

**CLINICAL STUDY PROTOCOL**

**Biomarker Assessment of Pomaglumetad on Glutamate Targets: Proof of Clinical Mechanism of Action (POCM)**

Abbreviated Title: Pomaglumetad Effects on Glutamate Biomarkers

Version #: 5.1

Version Date: 4/5/17

Contract Title: New Experimental Medicine Studies: Fast-Fail Trials in Psychotic Spectrum Disorders (FAST-PS)

Contract #: HHSN271201200007I

Contractor: Research Foundation for Mental Hygiene (RFMH)

Contract PI: Jeffrey A. Lieberman, MD

Lawrence C. Kolb Professor and Chairman of Psychiatry, Columbia University College of Physicians and Surgeons,

Director, New York State Psychiatric Institute

Psychiatrist-in-Chief, New York Presbyterian Hospital-Columbia University Medical Center

1051 Riverside Drive - Unit #4

New York, NY, 10032

Phone (646) 774-5300

Fax (646-774-5316)

Email: [jlieberman@columbia.edu](mailto:jlieberman@columbia.edu)

Task Order #: HHSN27100003

Overall Study PI: Jeffrey A. Lieberman

Sponsor: Jeffrey A. Lieberman

Funded by: National Institute of Mental Health

Abbreviated Study Title: Pomaglumetad Effects on Glutamate Biomarkers

Contract #: HHSN271201200007I

Task Order #: HHSN27100003

Coordinating Center [CC]: Research Foundation for Mental Hygiene (RFMH)

Data Management Center [DMC]: Nathan Kline Institute (NKI)

Site Principal Investigator(s) & Clinical Site(s):

| **Site PI** | **Site/Institution** | **Location** | **Phone** | **E-mail** |
| --- | --- | --- | --- | --- |
| Daniel C. Javitt | Columbia University (CU) | NY | 646-774-5404 | [dcj2113@columbia.edu](mailto:dcj2113@columbia.edu) |
| Adrienne Lahti | University of Alabama, Birmingham (UAB) | AL | 205-996-6776 | [alahti@uab.edu](mailto:alahti@uab.edu) |
| Donald Goff | New York University (NYU) | NY | 646-754-4843 | [Donald.Goff@nyumc.org](mailto:Donald.Goff@nyumc.org) |
| Stephen Marder | University of California Los Angeles (UCLA) | CA | 310-268-3647 | [marder@ucla.edu](mailto:marder@ucla.edu) |

Study Phase of clinical investigation:

Phase 1 ☐ No X Yes

Phase 1-2 ☐ No ☐ Yes

Phase 2 ☐ No ☐ Yes

Phase 3 ☐ No ☐ Yes

Target enrollment:

100 Healthy Volunteers across 4 sites (100 randomized for 81 completers); (25 subjects randomized per site for 20-21 completers per site)

FDA Approval(s)

IND/IDE ☐ No X Yes

Drug/Device: LY2140023 (Pomaglumetad Methionil, “POMA”)

IND/IDE #: 131215

Drug/Device supplied by: Lilly/DeNovo

IRB Approval(s):

Multi-Institutional Project ☐ No X Yes

| **Site/Institution** | **Date of IRB approval** |
| --- | --- |
| NYSPI (Central) |  |
| NYU |  |

NIMH Data and Safety Monitoring Board ☐ No X Yes

Samples are being stored ☐ No X Yes

Flesch-Kincaid reading level of consent form: 8th grade not including boiler plate language.

**PRÉCIS**

*Objective:* To evaluate the effects of the mGluR2/3 partial agonist LY2140023 (Pomaglumetad Methionil, “POMA”) at selected doses on ketamine-stimulated glutamate release in prefrontal cortex, as measured by pharmacoBOLD fMRI (also termed resting BOLD fMRI). Ketamine-induced glutamate release is hypothesized to simulate the synaptic dysregulation that occurs endogenously in the pathogenesis of schizophrenia. This study will determine the degree to which the POMA dose utilized in recent failed clinical trials (40 mg BID) is sufficient to engage the primary target (mGlu2/3 receptors) and whether a higher dose (160 mg BID) successfully engages the mGluR2/3 receptor. Both treatment arms will be compared only to placebo.

*Study population:* 100 healthy volunteers (100 randomized, for 81 completers)

*Design:* 3-arm randomized (1:1:1) double blind administration of 1) low dose (40 mg BID) POMA, 2) high dose (160 mg BID) POMA, or 3) placebo

*Outcome measures:* Ketamine-induced prefrontal glutamate activity as measured by PharmacoBOLD (primary).

Table of Contents

[1. INTRODUCTION/BACKGROUND/SIGNIFICANCE 10](#_Toc477339786)

[*1.1.* Introduction 10](#_Toc477339787)

[*1.2.* Background and Significance 11](#_Toc477339788)

[1.2.1. Preclinical/clinical support for use of mGluR2/3 agonists 11](#_Toc477339789)

[1.2.2. Rationale for PharmacoBOLD Methodology 13](#_Toc477339790)

[1.2.3. Ketamine Infusion Considerations 14](#_Toc477339791)

[2. STUDY OBJECTIVES 17](#_Toc477339792)

[*2.1.* Specific Aims 17](#_Toc477339793)

[3. SUBJECTS 17](#_Toc477339794)

[*3.1.* Description of Study Populations 17](#_Toc477339795)

[*3.2.* Inclusion Criteria 17](#_Toc477339796)

[*3.3.* Exclusion Criteria 18](#_Toc477339797)

[4. STUDY DESIGN AND PROCEDURES 19](#_Toc477339798)

[*4.1.* Study Overview 19](#_Toc477339799)

[*4.2.* Ketamine challenge protocol 19](#_Toc477339800)

[*4.3.* Biomarkers 19](#_Toc477339801)

[*4.4.* Recruitment 19](#_Toc477339802)

[*4.5.* Screening 20](#_Toc477339803)

[*4.6.* Study Day -10 (Visit 2) 20](#_Toc477339804)

[*4.7.* Study Procedures 20](#_Toc477339805)

[4.7.1. Investigational Product 22](#_Toc477339806)

[4.7.2. Pharmacokinetic Testing 23](#_Toc477339807)

[4.7.3. Study Day 1 (Visit 3) 23](#_Toc477339808)

[4.7.4. Study Day 5 (Visit 4) 23](#_Toc477339809)

[4.7.5. Study Day 10 (Visit 5) 23](#_Toc477339810)

[4.7.6. Pilot subjects 23](#_Toc477339811)

[*4.8.* Randomization Procedures 23](#_Toc477339812)

[*4.9.* Safety and Side Effects Measures 24](#_Toc477339813)

[*4.10.* Ketamine Methods 24](#_Toc477339814)

[4.10.1. Ketamine ratings 25](#_Toc477339815)

[4.10.2. Plasma ketamine/norketamine levels 25](#_Toc477339816)

[4.10.3. RUCDR genetic collection 25](#_Toc477339817)

[*4.11.* PharmacoBOLD Methods 25](#_Toc477339818)

[*4.12.* End of Participation and Follow-Up 26](#_Toc477339819)

[*4.13.* Early Termination 26](#_Toc477339820)

[5. STORAGE OF DATA AND SAMPLES 26](#_Toc477339821)

[*5.1.* Submission of Data to NIMH Data Archives 26](#_Toc477339822)

[6. ADDITIONAL CONSIDERATIONS 26](#_Toc477339823)

[*6.1.* Research with Investigational Drugs 26](#_Toc477339824)

[7. OUTCOME MEASURES 27](#_Toc477339825)

[*7.1.* Primary Outcome Measures 27](#_Toc477339826)

[*7.2.* Secondary Outcome and Safety Measures 27](#_Toc477339827)

[8. STATISTICAL ANALYSIS 27](#_Toc477339828)

[*8.1.* Analysis of Data/Study Outcomes 27](#_Toc477339829)

[*8.2.* Analyses by Specific Aims 28](#_Toc477339830)

[8.2.1. Primary Aim 28](#_Toc477339831)

[8.2.2. Secondary Aims 29](#_Toc477339832)

[*8.3.* Power Analysis 29](#_Toc477339833)

[9. HUMAN SUBJECTS PROTECTION 29](#_Toc477339834)

[*9.1.* Human Subjects Involvement, Characteristics, and Design 29](#_Toc477339835)

[9.1.1. Screening Methods 30](#_Toc477339836)

[9.1.2. Inclusion/Exclusion Criteria Rationale 30](#_Toc477339837)

[10. QUALIFICATIONS OF INVESTIGATORS 30](#_Toc477339838)

[*10.1.* Principal Investigator 30](#_Toc477339839)

[*10.2.* Site Principal Investigators 30](#_Toc477339840)

[*10.3.* Data Management Center 31](#_Toc477339841)

[11. ANTICIPATED BENEFIT 32](#_Toc477339842)

[12. RISKS AND DISCOMFORTS 32](#_Toc477339843)

[*12.1.* Potential Risks and Protections Against Risks 32](#_Toc477339844)

[12.1.1. Risks and Discomforts Associated with POMA Administration 32](#_Toc477339845)

[12.1.2. Risks and Discomforts Associated with Compound LY404039 in Humans 37](#_Toc477339846)

[12.1.3. Risks and Discomforts Associated with POMA in Animals 37](#_Toc477339847)

[12.1.4. Ketamine Administration 37](#_Toc477339848)

[12.1.5. MRI (PharmacoBOLD) Scans 39](#_Toc477339849)

[12.1.6. Venous Blood Sampling 43](#_Toc477339850)

[12.1.7. Intravenous Catheter 43](#_Toc477339851)

[12.1.8. Interviews and Behavioral and Safety Assessments 43](#_Toc477339852)

[12.1.9. Pregnant or Nursing Females 43](#_Toc477339853)

[12.1.10. Emergencies 44](#_Toc477339854)

[12.1.11. Safety Measures Upon Termination 44](#_Toc477339855)

[12.1.12. Genetic blood draw 44](#_Toc477339856)

[12.1.13. Recruitment and Informed Consent 45](#_Toc477339857)

[13. CLASSIFICATION OF RISK (FOR THE STUDY AS A WHOLE) 47](#_Toc477339858)

[*13.1.* Overall Risk and Benefit Consideration 47](#_Toc477339859)

[*13.2.* Children 47](#_Toc477339860)

[14. ALTERNATIVES TO PARTICIPATION OR ALTERNATIVE THERAPIES 47](#_Toc477339861)

[15. CONSENT DOCUMENTS AND PROCESS 47](#_Toc477339862)

[*15.1.* Designation of Those Obtaining Consent 47](#_Toc477339863)

[*15.2.* Consent Procedures 48](#_Toc477339864)

[*15.3.* Protection Against Risk 48](#_Toc477339865)

[16. SUBJECT SAFETY MONITORING 48](#_Toc477339866)

[*16.1.* Criteria for Stopping the Study or Suspending Enrollment or Procedures 49](#_Toc477339867)

[17. ADVERSE EVENT AND UNANTICIPATED PROBLEM REPORTING 50](#_Toc477339868)

[18. DATA AND SAFETY MONITORING 51](#_Toc477339869)

[*18.1.* NIMH DSMB Reporting 51](#_Toc477339870)

[19. QUALITY ASSURANCE 51](#_Toc477339871)

[*19.1.* Data Management and Procedures 51](#_Toc477339872)

[19.1.1. Data Management 51](#_Toc477339873)

[19.1.2. Data Collection Forms 52](#_Toc477339874)

[19.1.3. Data Acquisition and Entry 52](#_Toc477339875)

[19.1.4. Data Center Responsibilities 52](#_Toc477339876)

[19.1.5. Data Editing 52](#_Toc477339877)

[19.1.6. Documentation 52](#_Toc477339878)

[19.1.7. Training 53](#_Toc477339879)

[19.1.8. Data Lock 53](#_Toc477339880)

[*19.2.* Data Sharing 53](#_Toc477339881)

[19.2.1. NDCT Data Sharing 53](#_Toc477339882)

[*19.3.* Clinical Study Monitoring 54](#_Toc477339883)

[20. CONFIDENTIALITY 54](#_Toc477339884)

[*20.1.* Research Data and Investigator Medical Records 54](#_Toc477339885)

[*20.2.* Further Protection against Risk 55](#_Toc477339886)

[21. CONFLICT OF INTEREST 55](#_Toc477339887)

[22. TECHNOLOGY TRANSFER 55](#_Toc477339888)

[23. RESEARCH AND TRAVEL COMPENSATION 55](#_Toc477339889)

[24. REFERENCES 56](#_Toc477339890)

**LIST OF ABBREVIATIONS**

ACLS: Advanced Cardiac Life Support

BPRS: Brief Psychiatric Rating Scale

CADSS: Clinician Administered Dissociative Symptom Scale

CRA: Clinical Research Associate (monitor)

CRF: Case Report Form

CSV: Clinically Significant Values

CU: Columbia University

DMC: Data Management Center

DSMB: Data Safety Monitoring Board

EDC: Electronic Data Capture

EEG: Electroencephalography

EKG: Electrocardiogram

FFT: Fast Fourier Transform

FMRIB: Functional MRI of the Brain

GABA: Gamma-aminobutyric Acid

GCP: Good Clinical Practice

GCRC: General Clinical Research Center

Glu: Glutamate

Glx: Combined Glutamate and Glutamine

GSH: Glutathione

HCG: Human Chorionic Gonadotropin

HIC: Human Investigations Committee

ICC: Intraclass Correlation

IRB: Institutional Review Board

IEC: International Electrotechnical Commission

mPFC: Medial Prefrontal Cortex

mGluR: Metabotropic Glutamate Receptor

MRRC: Magnetic Resonance Research Center

NKI: Nathan S. Kline Institute for Psychiatric Research

NMDA: N-methyl-D-aspartate

NMDAR: N-methyl-D-aspartate-type Glutamate Receptor

nOe: Nuclear Overhauser enhancement

NYSPI/RFMH/CU: New York State Psychiatric Institute/Research Foundation for Mental Hygiene/ Columbia University

NYU: New York University

PCP: Phencyclidine

pharmacoBOLD: Blood Oxygenation Level Dependent Functional Magnetic Resonance Imaging (BOLD fMRI)

POC: Proof of Concept

POCM: Proof of Clinical Mechanism

POMA: Pomaglumetad Methionil (LY2140023)

rCBV: resting Cerebral Blood Volume

RF: Radiofrequency

RISE: Relational and Item Specific Encoding Task

ROI: Region of Interest

rsfMRI: Resting State fMRI

SAFTEE: Systemic Assessment for Treatment Emergent Events

SAR: Specific Absorption Ratio

SCID: Structured Clinical Interview for DSM Disorders

SOPs: Standard Operating Procedures

SPGR: Spoiled Gradient Recalled Echo

TCA: Tricarboxylic Acid

TSH: Thyroid Stimulating Hormone

TTL: Transistor-Transistor Logic

UAB: University of Alabama Birmingham

UCLA: University of California Los Angeles

# INTRODUCTION/BACKGROUND/SIGNIFICANCE

## Introduction

All currently approved treatments for schizophrenia, including both typical and atypical antipsychotics, function by blocking dopamine D_2_ receptors. Nevertheless, these compounds are effective for only a portion of individuals with schizophrenia, suggesting the need for alternative approaches (3, 4). Recent models of schizophrenia focus on disturbances in glutamatergic neurotransmission, particularly involving abnormalities in the N-methyl-D-aspartate-type glutamate receptors (NMDAR) and their pathophysiologic consequences (5, 6).

Glutamatergic models are strongly supported by the ability of NMDAR antagonists, such as ketamine or phencyclidine (PCP) to induce psychotomimetic effects that closely resemble symptoms of schizophrenia (5, 6). These effects are mediated, at least in part, by stimulation of presynaptic glutamate release in frontal brain regions (2, 7). In animal models, both the behavioral and neurochemical effects of ketamine administration are blocked by agonists of metabotropic (mGluR2/3) glutamate receptors, which have been localized to presynaptic glutamate terminals (2, 8). This finding led to the hypothesis that mGluR2/3 receptor agonists would be therapeutically beneficial in schizophrenia without the side-effects typically associated with antipsychotic treatment.

Based upon this model, several pharmaceutical companies developed mGluR2/3 agonists. The most promising of these compounds, LY2140023 (Pomaglumetad Methionil, “POMA”) showed significant reduction in acute symptoms of schizophrenia in an initial phase II study, but ultimately failed to show significant benefit in larger-scale phase III investigations (9-13). An unknown issue is whether the drug failed because the underlying stimulation of presynaptic glutamate release is non-predictive, or simply because of inadequate dosing. The present study utilizes a human adaptation of the preclinical ketamine model in order to assess mGluR2/3 target engagement by POMA both at the dose used in the prior clinical study (80 mg/day = 40 mg BID), and at the maximum tolerated dose (320 mg/day = 160 mg BID) for human studies. Lack of target engagement by the prior clinical-trial dose of 80 mg/day may indicate that an inadequate dose was used, but leaves open the possibility that higher doses might be efficacious. By contrast, if significant target engagement is observed at the prior clinical-trial dose, it could indicate that the ketamine-induced glutamate-increase model is non-predictive, and may decrease enthusiasm for continued development of this compound.

In the preclinical model, brain glutamate levels are measured directly through a dialysis probe implanted in target brain regions (7). Because this approach is not possible clinically, the present study uses ketamine-induced increases in prefrontal blood flow, as measured by functional MRI (resting BOLD fMRI or “pharmacoBOLD”) as a surrogate marker for local glutamate release. Prior rodent studies have demonstrated that ketamine-induced increases in glutamate are associated with increased local cerebral blood flow, which serve as an additional index of glutamate effect (14). In the clinical adaptation, increases in pharmacoBOLD response, which reflect local blood flow, are used instead of direct glutamate measures (reviewed in (15)).

Prior to initiation of the FAST-PS project, several studies had already evaluated ketamine-concentrations needed to elicit significant psychosis in humans and had ketamine-induced increases in human frontal cortex (reviewed in (15)). In the initial, “biomarker validation” stage of this project, we replicated these findings and demonstrated their feasibility and utility for cross-site investigation. In the present study, we will utilize the ketamine-induced increase in ongoing BOLD response – alternately termed “resting BOLD fMRI” or “pharmacoBOLD” – to evaluate the degree of mGluR2/3 target engagement – measured as inhibition of ketamine-induced changes in pharmacoBOLD – obtained during sustained exposure to POMA at 40 BID and 160 BID.

## Background and Significance

Antagonists of NMDAR such as ketamine or PCP induce clinical symptoms that closely resemble schizophrenia, suggesting first that endogenous dysfunction of NMDAR may contribute significantly to the pathophysiology of schizophrenia, and, second, that agents capable of enhancing NMDAR-mediated neurotransmission may significantly ameliorate clinical symptoms (16-20). Despite this strong preclinical model, however, novel medications based upon glutamatergic models of schizophrenia, including mGluR2/3 agonists, have so far failed in phase III clinical trials. Whether or not adequate doses were used in prior clinical trials, however, remains to be determined. The present study utilizes ketamine-induced pharmacoBOLD to investigate whether doses of POMA used in a recent negative phase III clinical trial (i.e., 40 mg BID) were adequate to inhibit presynaptic glutamate release as predicted by the preclinical model and whether a substantially higher dose (i.e., 160 mg BID) might show superior target engagement. Demonstration of such an effect would suggest that prior trials might have failed because of inadequate dosing, and that compounds with higher levels of target engagement might show improved efficacy.

### Preclinical/clinical support for use of mGluR2/3 agonists

Group II metabotropic receptors (mGluR2/3) regulate presynaptic glutamate release and postsynaptic sensitivity by limiting glutamate release during conditions of glutamate spillover from the synaptic cleft (21). The use of mGluR2/3 agonists in schizophrenia is based upon the hypothesis that increased cortical glutamate levels may be pathophysiological, and that compounds such as mGluR2/3 agonists might be capable of reducing abnormal glutamate levels in schizophrenia and might therefore be therapeutically beneficial (2, 7, 22, 23). For example, in one early study the compound LY354740 (the active metabolite of POMA) was found to fully reverse PCP-induced glutamate increases in rodent prefrontal cortex (**Fig. 1**), along with behavioral manifestations of PCP effect (e.g., locomotor hyperactivity). mGluR2/3 agonists similarly are reported to attenuate the disruptive effects of ketamine on working memory in humans (24). Among brain regions mGluR2/3 receptors are located extensively in the prefrontal cortex, suggesting a particular impact on cognition and positive symptoms as reviewed in (25).

**Fig. 1**. Effect of pretreatment with the group II mGluR agonist LY354740 (10 mg/kg ip injection) (filled circles; n = 6) or vehicle (open circles; n = 7) on stimulation of glutamate efflux by PCP (5 mg/kg ip injection) in the prefrontal cortex of freely moving rats. PCP injection after vehicle (water) produced a significantly greater effect on extracellular glutamate concentrations in the prefrontal cortex (P <0.05) than in animals pretreated with LY354740 (2).

Clinical support for the mGluR2/3 agonist approach was provided initially by a 4 week phase II study with POMA used at a dose of 40 mg BID (26). However, these initial positive results were not replicated in subsequent 6 week studies using doses of up to 80 mg BID (27), leading to discontinuation of clinical development. Other mGluR2/3 agonist compounds such as AZD8529 (Astra/Zeneca) or ADX71149 (mGluR2-PAM; Addex/Janssen) have also been found to be well tolerated during repeated treatment, but also failed to show significant beneficial effect when used clinically. As with POMA, the degree to which these compounds engaged presynaptic mGluR2/3 receptors remains unknown.

In rodents, the increase in glutamate release can also be observed indirectly via modulation of regional cerebral blood flow using high field imaging. Moreover, effects of PCP on regional cerebral blood volume (rCBV) are reversed by the mGluR2/3 agonist LY354540 (the active metabolite of POMA), but not by the D_2_ antagonist raclopride, suggesting relevance to antipsychotic-resistant symptoms of schizophrenia. Clozapine partially reverses the PCP effect although to a lesser extent than LY354740, further supporting potential clinical relevance of the glutamate model (**Fig. 2**).


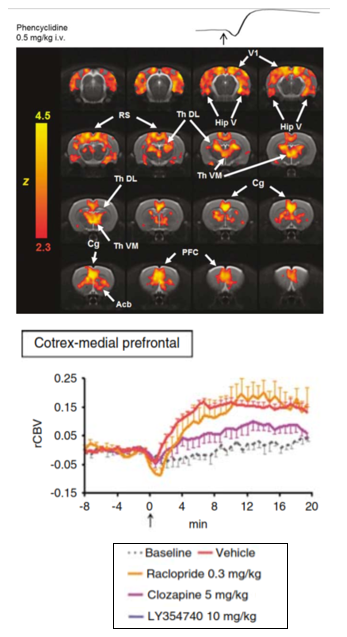


**Fig. 2**: *Top*: Effect of the NMDAR antagonist PCP on regional cerebral blood volume (rCBV) showing increases in regions of relevance to the present project, including prefrontal cortex (PFC) and cingulum (Cg). *Bottom:* Time course plot showing reversal of the PCP effect on rCBV by the mGluR2/3 agonist LY354740 (active metabolite of POMA). By contrast, neither vehicle nor the D2 antagonist raclopride significantly affected rCBV (1).

Based upon the above preclinical data, increases in glutamate release/metabolism have been considered a potential pathophysiological process in schizophrenia, and therefore a potential target for drug development. In the present study, we use ketamine-induced pharmacoBOLD in the prefrontal cortex in healthy volunteers as a direct human analog of the rodent findings and as a bridge to potential clinical trials in schizophrenia. The goal of the study is to determine whether POMA at the doses used in prior clinical studies was sufficient to modulate prefrontal glutamate levels, as measured by pharmacoBOLD, and (if not) whether significant target engagement would be observed at higher levels.

In the current protocol, we therefore evaluate the effects of two doses of POMA: 1) 40 mg BID and 2) 160 mg BID vs. placebo. POMA itself was chosen due to its availability, and validated mGlu2/3 agonism and safety (see Section 11.1). The 40 mg BID dose was chosen to reflect the dose primarily used in the phase III clinical investigations. The 160 mg BID dose is higher than that used in clinical studies, and is less than the maximum tolerated daily dose based upon phase I safety investigations, in accordance with the POMA investigators brochure (Section **6.2.1.1**). Prior mechanistic experiments conducted by Lilly (Adam Schwartz, personal communication, IB section 6.1.2) showed that 80 mg BID may reverse ketamine induced changes in PharmacoBOLD acutely. An exposure of 10 days was chosen to allow for an assessment of the effects of repeated, sustained dosing across 10 days to better recreate the clinical trial conditions. Specifically, the 10-day limit is consistent with phase 1 studies at 160 BID (IB Section 6.2.1.1).

Although definitive interpretation of a single target engagement study is always difficult, nevertheless we consider the following study outcomes and interpretations as most relevant to future decisions with regard to future development of POMA or other high affinity mGluR2/3 agonists.

| **Table 1: Effects of POMA at indicated dose on ketamine-induced pharmacoBOLD increase, and interpretation of effect** | | |  |
| --- | --- | --- | --- |
| **Low dose POMA** | **High dose POMA** | **Interpretation** | |
| No significant effect | No significant effect | No target engagement even with maximum tolerated dose of POMA. Compound had no demonstrable effect. Unclear if this is because 1) rodent model is non-predictive in humans, or 2) the drug produces insufficient central receptor occupancy in humans even at maximum tolerated dose. | |
| No significant effect | Significant Effect | Dose used in prior clinical investigations was probably inadequate. Future clinical studies with high dose POMA or other high affinity mGluR2/3 agonists may be warranted. | |
| Significant Effect | No significant effect | Dose used in prior clinical studies was adequate to significantly engage the mGluR2/3 but nevertheless failed to produce significant beneficial effects. Either 1) the mechanism is not predictive of significant beneficial effect (“fail mechanism”) or 2) the clinical population selected was not enriched in the primary pathophysiological process (increased glutamate levels), in which case future studies targeted only at individuals with increased brain glutamate levels may be warranted. | |
| Significant Effect | Significant Effect |  |  |

### Rationale for PharmacoBOLD Methodology

Biomarkers for the present study were based upon results of the preceding FAST-PS Biomarker Validation study, which demonstrated feasibility of use of the ketamine-induced pharmacoBOLD in multicenter clinical investigations.


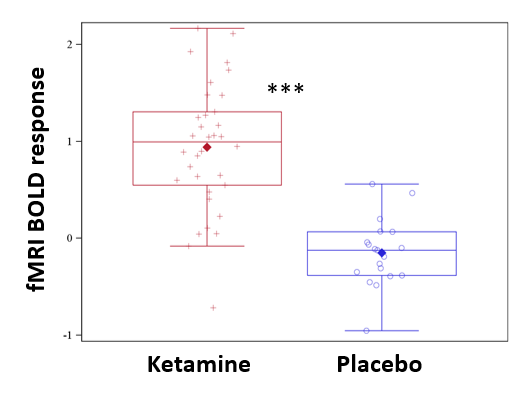


**Fig. 3:** Change in pharmaco BOLD fMRI with ketamine in recently completed FAST-PS Biomarker Validation study. *** p<.001 vs. placebo

In healthy volunteers, ketamine induces significant increases in PharmacoBOLD, particularly in frontal, cingulate, and temporal regions that correlate with its acute psychotomimetic activity (28). This measure, moreover, likely reflects underlying glutamatergic modulation and corresponds closely to resting Cerebral Blood Volume (rCBV) effects observed in rodents (1, 14). These data suggest that the ketamine/PharmacoBOLD method may be useful as a target engagement biomarker for either mGluR2/3 agents. Therefore, in this study, we will evaluate effects of POMA on ketamine-induced changes in PharmacoBOLD (29).


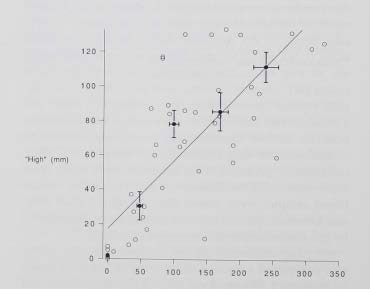


**Fig. 5**: Dose-response relationship of plasma ketamine levels vs. drug induced “high”, showing moderate-high level psychotomimetic effects induced by levels (150 ng/ml) produced by the proposed dosage regimen [55].

**Fig. 4**: Plasma ketamine levels obtained the using proposed administration protocol. [54]


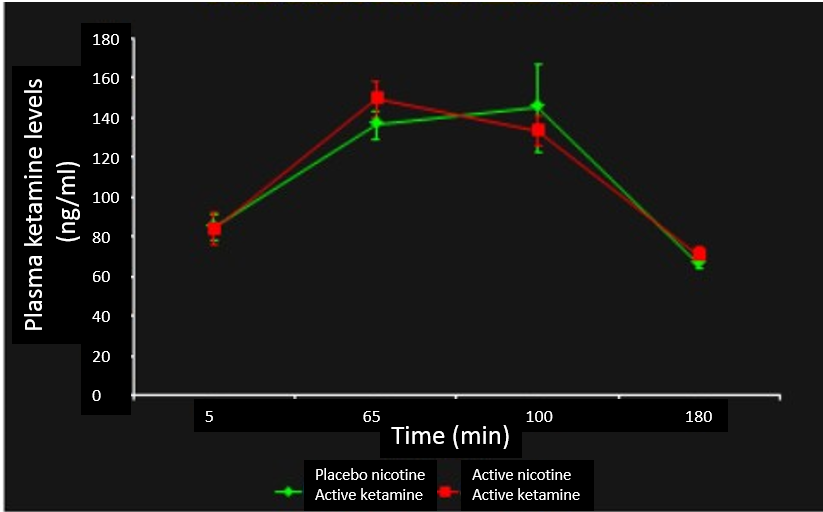


Our recently completed FAST-PS Biomarker Validation study provided an estimate of effect size of the PharmacoBOLD response to ketamine, permitting implementation in the present project. Preliminary results demonstrate highly significant change in PharmacoBOLD (t_49_=6.61, p<0.0001; **Fig. 3**), with an effect size of 3.1 between active and placebo exposure. Furthermore, the response X site interaction was non-significant, reflecting a similar effect across sites. The large effect-size suggests that there is significant magnitude of response to detect reversal of the effect by a test agent (i.e., POMA). Modest correlation to symptoms was observed across the placebo and ketamine groups (r=0.31, p=.002), suggesting relevance to clinical symptoms of schizophrenia, although the effects were not independently significant within the ketamine group alone (r=0.04, p=0.8).

### Ketamine Infusion Considerations

Ketamine infusion is widely used in both mechanistic research for schizophrenia and clinical treatment for depression. In initial studies with ketamine in the early 1960s, it was noted that ketamine produced what would now be considered positive, negative and cognitive symptoms of schizophrenia (5). Symptomatic effects of NMDA blockade were better classified starting in the early 1990s in a series of ketamine challenge studies conducted in both normal volunteers and schizophrenia patients. In normal volunteers, significant increases in positive, negative, and cognitive symptoms were observed in similar proportions as in schizophrenia (6).

As previously reviewed (21), ketamine also reproduces core neuropsychological abnormalities of schizophrenia including executive functioning, attention/vigilance, verbal fluency, and visual and verbal working memory. Moreover, in monkeys treated with ketamine, characteristic, schizophrenia like deficits in a task-switching paradigm are reproduced. Ketamine infusion also reproduces both the severity and type of thought disorder seen in schizophrenia with both, for example, being associated with high levels of poverty of speech, circumstantiality and loss of goal, and relatively low levels of distractive or stilted speech or paraphasias.

Although many groups are currently conducting ketamine research, the precise protocols used have varied across groups and studies within groups, and no consensus presently exists regarding optimal dose. The present study will utilize a modified version of the ketamine dose regimen as used in the recently completed FAST-PS Biomarker validation study, which provided preliminary results for the present investigation. The dose was selected to provide a robust change in pharmacoBOLD response, in order to optimize power for detecting reversal by potential test agents, while remaining low enough to minimize adverse effects and premature terminations.

| **Table 2** | **Mean [SD] (min-max) plasma concentration (ng/mL)** | |
| --- | --- | --- |
| **Experimental group** | **Ketamine** | **Norketamine** |
| vehicle | 3.76 [6.93] (BQL -21.36) | 4.08 [12.23] (BQL -37.76) |
| 5 mg/kg | 282.33 [164.52] (131.99-637.59) | 305.03 [172.06] (139.46-659.58) |
| 10 mg/kg | 601.81 [181.83] (391.56-893.39) | 527.72 [259.08] (329.13-1065.74) |
| 25 mg/kg | 1693.87 [475.92] (922.1-2516.49) | 1333.01 [585.78] (800.88-2377.84) |

Our prior study utilized a 0.23 mg/kg bolus over 1 minute followed by 0.58 mg/kg/hr over 30 minutes then 0.29 mg/kg/hr over 29 minutes (30, 31), which is the highest dose currently in use by research groups pursuing ketamine research. As noted above, this dose and length of administration has been safely used by the current investigators (30, 31), including in the preceding FAST-PS Biomarker Validation project, and allows for a steady state dose and the collection of a full imaging paradigm (**Fig. 4**). Moreover, this infusion paradigm produces ketamine levels that are known to be associated with moderate-high level psychotomimetic effects (**Fig. 5**), without excessive sedation, agitation, or other features that would preclude its routine use. Finally, these levels are associated with rodent ketamine doses used for establishment of basic physiological effects (~5 mg/kg) (**Table 2)**. At much higher doses (>25 mg/kg), it is known that effects of ketamine on glutamate functioning may be lost. However, plasma levels achieved at these high doses are much greater than the levels observed during the proposed clinical infusion schedule. In accordance with the FDA, the present project, we will limit our ketamine dose to a 0.23 mg/kg bolus over 1 minute, for a maximum of 20 mg per infusion.

*Safety*: Although persistent psychosis related to ketamine exposure is a theoretical concern, ketamine has been extensively used to date and risk of persistent psychosis appears low in well-screened individuals (6, 32). A greater issue is acute nausea/vomiting, which can be induced especially by bolus infusion. Outside of the scanner, nausea can be managed relatively easily. The present study, however, will utilize infusion within the scanner, with nausea potentially requiring cessation of the scan and loss of data. For this study, we will use a 0.23 mg/kg bolus over 1 minute, which is associated with low risks of nausea/vomiting.

Extensive experience suggests that ketamine administration is not associated with long-term toxicity. The doses of ketamine administered produce blood levels 1/3 - 1/6 those associated with blood levels achieved when ketamine is used as a primary surgical anesthetic. Since 1989, members of the study team have successfully administered ketamine on over 800 occasions to over 300 subjects. We have extensive experience using ketamine in psychiatric research. While not exhaustive, **Table 3** reviews some of the ketamine studies that we have conducted over the years in healthy subjects. Studies have been conducted in the context of research on depression, alcoholism and schizophrenia, among other conditions.

| **Table 3** | | | | | |
| --- | --- | --- | --- | --- | --- |
| **Ketamine study** | **Infused dose of ketamine** | | **Subjects,**  **N** | **Time**  **point of measuring (min)b** | **Publication** |
|  | **Bolus** | **Continuous**  **infusion** |  |  |  |
| Dose-response  and genetic studies of ketamine | 1) 0.5mg/kg over  40 min |  | 38 | 10 | Partly  reported in (6) |
|  | 2) 0.1mg/kg over  40 min |  | 38 |  |  |
| Interaction with lorazepam | 1-min 0.26  mg/kg | 0.65 mg/kg over 60  min | 23 | 5 | (33) |
| Interaction with haloperidol | 1-min 0.26  mg/kg | 0.65 mg/kg over 60  min | 20 | 5 | (34) |
| Interaction with lamotrigine | 1-min  0.26mg/kg | 0.65mg/kg over 90  min | 17 | 5 | (35) |
| Interaction with amphetamine | 1-min 0.23  mg/kg | 0.5 mg/kg over 60  min | 32 | 1 | (35) |
| Interaction with naltrexone | 1) 0.081mg/kg  over 10 min | 0.00675mg/kg/min  over 60min | 24 | 10 | (36) |
|  | 2) 0.081mg/kg  over 10 min | 0.04 mg/kg/hr over  60min | 18 |  |  |
| Interaction with LY354740 | 1-min 0.26mg/kg | 0.65mg/kg/hr over  100 min | 19 | 5 | (24) |
| Interaction with nicotine | 1-min  0.26mg/kg | 0.65mg/kg/hr over  120 min | 4 | 1 | (30) |

Ketamine produces transient perceptual and cognitive changes that resolve by the end of each test day. More recently we have begun to follow subjects 1, 3 and 6 months after study participation; these follow up data do NOT reveal ANY long-term adverse effects of ketamine (32). The dose of ketamine that will be utilized in this study is well within the range of ketamine doses that we have shown to be safe. In addition, ketamine has been found to not produce sensitization, and repeated administration has been safely done in the preceding FAST-PS Biomarker Validation study.

# STUDY OBJECTIVES

This study evaluates the effects of POMA at selected doses on a ketamine-stimulated glutamate increase in the prefrontal cortex hypothesized to simulate the synaptic dysregulation that occurs endogenously in the pathogenesis of schizophrenia. The purpose of this is: 1) to determine whether the low dose (40 mg BID) utilized in recent (failed) clinical trials is sufficient to engage the primary target and 2) whether a higher (160 mg BID) dose, representing the maximum tolerated dose of POMA, engages the target to a greater degree. In addition, this study will further validate PharmacoBOLD as a biomarker of mGluR2/3 target engagement, permitting its future use in other glutamatergic drug development programs. See Section 8.2. for full description of primary and secondary specific aims.

## Specific Aims

Primary Aim – Specific Aim 1: To assess the degree of target engagement obtained following *sustained* exposure with low (40 mg BID) or high (160 mg BID) doses of POMA vs. placebo. “Sustained” exposure effect is defined as the effect observed on Day 10 of blinded exposure. Target engagement will be assessed by the drug’s ability to inhibit ketamine-induced prefrontal Glu release, as evaluated by PharmacoBOLD. To determine this, the degree of ketamine-induced Glu release observed during the Day 10 challenge within each active arm will be compared to the degree of release observed within the placebo group, and secondarily, to the degree of release observed within the other active arm.

# SUBJECTS

## Description of Study Populations

81 healthy volunteers (27 per arm) are expected to complete Day 10 study procedures across the 4 study sites. We will randomize up to 100 subjects to obtain 81 completers. Withdrawals/dropouts will be replaced, if feasible. Additionally, up to 3 pilot subjects per site will complete screening and Visit 2 procedures only. These subjects will not receive POMA, and will be used to demonstrate familiarity with procedures and cross-site consistency before enrolling subjects into the main study protocol. (Pilots are optional.) Pilots may be enrolled in the main study after 30 days (exclusion criteria 3).

## Inclusion Criteria

1. Males or females between 18 to 55 years old at the screening visit
2. Medically healthy, as assessed by study physician
3. Capable of understanding the study procedures and able to provide informed consent
4. Eligible men and women must agree to use a reliable method of birth control (for example, use of oral contraceptives or Norplant®; a reliable barrier method of birth control diaphragms with contraceptive jelly; cervical caps with contraceptive jelly; condoms with contraceptive foam; intrauterine devices; partner with vasectomy; or abstinence) during the study. Women who are post-menopausal or otherwise not of childbearing potential are also eligible.

## Exclusion Criteria

1. Current or past Axis I psychiatric history (including Substance Use Disorder/Alcohol Use Disorder, with the exception of nicotine use disorder)
2. Positive urine toxicology
3. History of recreational ketamine use, recreational PCP use, or an adverse reaction to ketamine. Subjects who have participated in prior research ketamine studies will be eligible. Subjects can have infusions not more frequently than biweekly, and not more than 1/month on average, therefore subjects entering the study will need to wait one month if they had a single infusion and 6 weeks if they have had two closely spaced infusions.
4. History of first-degree relative with schizophrenia
5. History of violence, including any history of using a gun, knife, or other weapon with intent to harm someone, as well as a more than one physical fight without a weapon after the age of 18 years old (not including fights that happen during sports competition).
6. Presence or positive history of significant medical illness, including renal problems (GFR<60), high blood pressure (defined as systolic blood pressure (SBP) > 140 or diastolic blood pressure (DBP) > 90), low blood pressure (SBP < 100, DBP < 60), orthostatic blood pressure at baseline (change in mean arterial pressure [1/3 systolic + 2/3 diastolic] of > 20%), cardiac illness, or clinically significant abnormal screening labs, as determined by the site physician.
7. Presence or positive history of neurological illness, including seizures, mental retardation or any other disease/procedure/accident/intervention associated with significant injury to or malfunction of the central nervous system (CNS), or history of significant head injury.
8. Pregnancy or breast-feeding. This exclusion criterion applies only to females of child-bearing potential (not surgically sterilized and between menarche and 1 year postmenopausal). Must test negative for pregnancy at the time of screening based on a serum pregnancy test.
9. Metal implants, pacemaker, other metal (e.g., shrapnel or surgical prostheses) or paramagnetic objects contained within the body which may present a risk to the subject or interfere with the MR scan.
10. Medicinal patch, unless removed prior to the MR scan
11. Currently taking any psychotropic medication, including antidepressant medications, benzodiazepines, antipsychotic medications, mood stabilizers, anti-epileptic medications, and stimulants. We will exclude any subject who requires treatment with any psychotropic medication from one of these classes.
12. Claustrophobia
13. Subjects with suicidal ideation with intent or plan (indicated by affirmative answers to items 4 or 5 of the Suicidal Ideation section of the baseline C-SSRS) in the 6 months prior to screening or subjects who represent a significant risk of suicide in the opinion of the investigator.
14. Weight >86.95 kg (191.6 pounds)
15. Subthreshold PhamacoBOLD response (defined as 0.5% peak ketamine response) during screening ketamine infusion.

# STUDY DESIGN AND PROCEDURES

## Study Overview

This is a POCM study of the effects of POMA on ketamine challenge induced glutamate activity, utilizing PharmacoBOLD (primary) activity in healthy volunteers. Subjects will be randomized to 1 of 3 arms consisting of:

1. Low dose POMA (40 mg BID)
2. High dose POMA (160 mg BID)
3. Placebo

We will randomize 100 subjects total to obtain 27 completers per arm (81 total). All subjects will undergo a 10-day exposure of POMA or placebo with ketamine challenge investigation to be conducted following final (Day 10) study compound (POMA or placebo) administration, as outlined below in **Table 4**. Safety assessments will be obtained weekly throughout the study (**Table 4**).

## Ketamine challenge protocol

The ketamine challenge protocol [0.23 mg/kg bolus over 1 minute] was modified from the methods used in our preceding FAST-PS Biomarker Validation study to comply with new FDA requirements to limit total ketamine dose to 20 mg. See Sections 1.2.3. and 4.9. for further discussion of dosing considerations.

## Biomarkers

The primary outcome measure is ketamine-induced increase in PharmacoBOLD activity (pharmacoBOLD response) obtained at time points selected for maximal sensitivity. See Section 1.2.2. for discussion of rationale.

## Recruitment

The enrollment sites have substantial experience in recruitment and maintain registries of well-screened healthy volunteers who can be selected based on a database containing relevant demographic and medical information; this approach is the preferred approach since subjects are pre-screened and a representative sample can be selected based on demographic information. Additional subjects will be recruited through advertisements in local newspapers, on research websites, or on sites such as Craig’s List. “Umbrella” approaches, such as listing this study on ClinicalTrials.gov, will also be used. All recruitment materials will be approved by the relevant institutional review boards (IRBs) prior to distribution. Recruitment procedures will be performed by study physicians and investigators, assisted by research staff.

## Screening

*Screening/Informed consent*: Informed consent will be obtained before any study procedures, including screening procedures, are initiated. After providing informed consent, subjects will undergo full medical screening (medical history, physical examination, vital signs, laboratories for basic chemistries, blood counts, liver function tests, urinalysis, urine toxicology, serum pregnancy test for women, and thyroid tests, electrocardiogram [EKG]) and psychiatric screening (i.e., Structured Clinical Interview for DSM V Axis I Disorders (SCID) to confirm eligibility.

Screening will occur over an up to 31-day screening period, including the screening ketamine MRI (**Table 4: V2**).

## Study Day -10 (Visit 2)

After receiving medical and psychiatric screening, eligible subjects will return to the study center to undergo the screening ketamine challenge and assessments. Upon arrival, they will provide urine for drug and pregnancy (females only) testing, and receive an intravenous catheter for challenge administration, as well as ketamine/norketamine blood level. They will receive ketamine [0.23 mg/kg bolus over 1 minute] as well as PharmacoBOLD imaging assessments. All subjects will be monitored by EKG and Advanced Cardiac Life Support (ACLS) - certified physician or RN and study staff. An MD will be present for the duration of the ketamine infusion. Vital signs will be assessed and side effects/adverse events will be queried. All subjects will also be monitored using the BPRS and CADSS pre and post ketamine infusion. Subjects will be allowed to leave and go home after clearance by the study physician. Although we expect all subjects to tolerate the ketamine challenge, in cases where a subject does not tolerate it well, all sites have the capability of holding the subject for further observation and, if needed, admitting a subject to an inpatient unit for extended monitoring and treatment as necessary. The site study physician will call subjects one day after their screening ketamine infusion. Subjects with a subthreshold PhamacoBOLD response during screening ketamine infusion will not be eligible for randomization.

## Study Procedures

After screening procedures, eligible subjects will begin a 10-day trial of blinded POMA vs. placebo*.* Ketamine-induced prefrontal glutamate activity will be measured by PharmacoBOLD (primary) on Day 10 of exposure (POMA or placebo).

**Table 4. Study Procedures**

|  | **Evaluation/**  **Screening** | **Ketamine**  **Screening** | **Follow-up**  **Phone call** | **Study**  **Day 1** | **Follow-up**  **Phone call** | **Study**  **Day 5** | **Follow-up**  **Phone call** | **Study**  **Day 10** | **Follow-up**  **Phone call(s)** |
| --- | --- | --- | --- | --- | --- | --- | --- | --- | --- |
|  | Visit 1  (V1) | Visit 2  (V2) | *In person as needed, see Section 4.11. | Visit 3  (V3) | *In person as needed, see Section 4.11. | Visit 4  (V4) | *In person as needed, see Section 4.11. | Visit 5  (V5) | *In person as needed, see Section 4.11. |
|  | Day (-31 to -11) | Day -10±2 | Day -9 | Day 1 | Day 2-4 | Day 5±1 | Day 6-9 | Day 10±1 | One day, one week, and one month after V4 |
| Subject Registration | X |  |  |  |  |  |  |  |  |
| Structured Clinical Interview | X |  |  |  |  |  |  |  |  |
| Phone Call Check In |  |  | X |  | X |  | X |  | X |
| Visit Form | X | X | X* | X | X* | X | X* | X | X* |
| Inclusion/Exclusion Checklist | X |  |  |  |  |  |  |  |  |
| Brief Psychiatric Ratings Scale (BPRS) |  | X |  |  |  |  |  | X |  |
| Clinician Administered Dissociative Symptom Scale (CADSS) |  | X |  |  |  |  |  | X |  |
| Medical History | X |  |  |  |  |  |  |  |  |
| Physical Exam | X |  |  |  |  | X |  | X |  |
| Demographics | X |  |  |  |  |  |  |  |  |
| Complete Blood Count | X |  | X* |  | X* | X | X* | X | X* |
| Blood Chemistry (including liver function, TSH) | X |  | X* |  | X* | X | X* | X | X* |
| Urinalysis | X |  | X* |  | X* | X | X* | X | X* |
| Urine Drug Screen | X | X | X* | X | X* | X | X* | X | X* |
| Pregnancy Test (Serum/Urine) | S | U |  | U |  | U |  | U |  |
| Medication packet dispensation |  |  |  | X |  | X |  |  |  |
| Medication packet assessment |  |  |  |  |  | X |  | X |  |
| RUCDR genetic collection, by consent (tiered) |  |  |  | X |  |  |  |  |  |
| Electrocardiogram | X | X | X* | X | X* | X | X* | X | X* |
| Vital Signs  (including height and weight) | X | X | X* | X | X* | X | X* | X | X* |
| SAFTEE (side effects) |  | X |  | X |  | X |  | X |  |
| Ketamine Challenge |  | X |  |  |  |  |  | X |  |
| POMA levels |  |  |  | X |  | X |  | X |  |
| Ketamine Levels |  | X |  |  |  |  |  | X |  |
| MRI PharmacoBOLD |  | X |  |  |  |  |  | X |  |
| Prior and Concomitant Medications | X | X | X* | X | X* | X | X* | X | X* |
| Adverse Event Form |  | X | X | X | X | X | X | X | X |
| C-SSRS | X | X | X | X | X | X | X | X | X |
| Study Completion/Early Termination |  |  | X* |  | X* |  | X* |  | X* |
| ***Note that CRF forms will only be completed if an in person visit is required** | | | | | | | | | |

### Investigational Product

All subjects will begin 10-day sustained exposure to POMA vs. placebo according to their randomized assignment to 1 of 3 dosing groups consisting of 1) 40 mg BID, 2) 160 mg BID, or 3) placebo. Study drug compliance will be ensured by pill counts. These values will be entered into the study database. While compliance (pill counts) will not be considered for the intent to treat analysis, compliance will be considered in the per protocol population analysis. For per protocol analyses, only subjects who are >80% medication-compliant will be included.

Exposure will be double-blind, with matching active and placebo tablets provided by Denovo. All subjects will receive four tablets BID (8 tablets total, **Table 5**), starting on Day 1.

Subjects will undergo an initial 5-day dose titration before 5±1 days of a fixed dose. Subjects will be expected to titrate up to a full dose by Day 5, but the titration schedule may be adjusted at the site study physician’s discretion. All subjects must reach full dose by Day 6 or will be discontinued from the study. To maintain the double-blind, all subjects will receive 4 active/placebo tablets BID. The titration is being done to reduce the risk of nausea and vomiting.

**Table 5. Study drug dosing schedule**

| Day | High dose | Low dose | Placebo |
| --- | --- | --- | --- |
| 1 | 40 mg in AM | 40 mg in AM | Placebo |
| 2 | 40 mg BID | 40 mg BID | Placebo |
| 3 | 80 mg BID | 40 mg BID | Placebo |
| 4 | 120 mg BID | 40 mg BID | Placebo |
| 5-10±1 | 160 mg BID | 40 mg BID | Placebo |

### Pharmacokinetic Testing

Venous blood samples (10 mL each) will be drawn before and 180 minutes after taking the morning dose of POMA or placebo on Days 1, 5, and 10 and will be stored for future analysis.

### Study Day 1 (Visit 3)

After receiving medical and psychiatric screening to confirm eligibility, eligible subjects will be randomized to POMA or placebo. They will return to the study center for their first study drug administration. Upon arrival they will provide urine for drug and pregnancy (females only) testing, and receive their Day 1 dose, as well as POMA blood level sampling and RUCDR whole blood collection. Departing subjects will be given a 6-day supply of study drug medications (5d + 1d “cushion” in case of missed visit). The site study physician will call subjects daily, and adjust the titration schedule at their discretion. Modifications to the titration schedule may involve an additional visit to the clinic.

### Study Day 5 (Visit 4)

Subjects will return for a medical checkup, including blood draw, urine sample, vitals, EKG, physical examination, medication packet assessment, C-SSRS, discussion of medication use in the prior week, POMA levels, and SAFTEE (including assessment of any skin rashes). Women of child bearing age will undergo urine pregnancy testing. If the subject is tolerating the medication, they will receive an additional 6-day supply of study drug. The site study physician will call subjects daily, and adjust the titration schedule at their discretion through Day 6.

### Study Day 10 (Visit 5)

Subjects who successfully complete the 10 day POMA or placebo course will return for Day 10 procedures, including a physical examination. If the subject is tolerating the medication and is compliant (assessment of medication packet), subjects will receive their last dose of study drug prior to their second dose of ketamine (i.e., they will receive the same drug/infusion sequence they received during Day -10 – Visit 2 Ketamine screening). At the end of this session we will repeat a full laboratory evaluation (i.e., basic chemistries, liver function tests, complete blood count, thyroid stimulating hormone (TSH), and urinalysis), and SAFTEE (including assessment of any skin rashes). Women of child bearing age will undergo urine pregnancy testing. Subjects will only be asked to return for a follow-up visit if abnormalities are found on the Day 10 labs. A detailed breakdown of visit-by-visit procedures can be found in **Table 4.**

### Pilot subjects

In order to pilot procedures, up to three subjects at each site will complete screening (Visit 1) and Study Day -10 (Visit 2) procedures. These subjects will not receive POMA. (Pilots are optional.)

## Randomization Procedures

Subjects will be randomized to 1 of 3 arms consisting of:

1. Low dose POMA (40 mg BID)
2. High dose POMA (160 mg BID)
3. Placebo

We will randomize 100 subjects total to obtain 27 completers per arm (81 total), using blocks of three, with stratification by site and screening ketamine pharmacoBOLD response. The DMC will provide randomization lists to the individual pharmacies. Dropouts after Day 1 will be replaced by another patient with the same group assignment. Subjects and study staff will remain blinded to group assignment until database is locked, unless a medical condition in a subject necessitates unblinding.

## Safety and Side Effects Measures

A full laboratory evaluation (i.e., basic chemistries, liver function tests, complete blood count, thyroid stimulating hormone (TSH), and urinalysis) and a physical examination will be completed at screening, Day 5 and Day 10. All subjects will receive constant electrocardiogram (EKG) monitoring during the study procedures on Days -10 and 10. The SAFTEE (37) will be used to assess general side effects and will be performed after study procedures on Days -10, 1, 5, and 10, as well as vital signs assessment. If necessary, subjects will be asked to return one week after completion of the 10-day study to follow up on any abnormalities in EKG, physical examination, or laboratories. Women of child bearing potential will undergo pregnancy testing on every study visit. Vital signs, including orthostatic blood pressure monitoring, will also occur on the study procedure days. Site-specific ranges for lab values will be utilized to determine inclusion/exclusion, accept for those values specifically referenced in the inclusion/exclusion criteria. All laboratory values will be reviewed by a study physician.

**Clinical significant values (CSV):** Abnormal lab values deemed clinically significant by study physician may be handled in the following ways (based on clinical judgment). For clinically significant lab values at Day 10, subject will be notified and scheduled for repeat blood testing within 7-10 days of Day 10 visit. If clinically indicated, subjects will be referred for follow up. If immediate medical attention is needed, subjects will be instructed to go to Emergency Department.

## Ketamine Methods

The Day -10 and Day 10 ketamine procedures will consist of a racemic ketamine hydrochloride (Ketalar; Parke Davis, Morris Plains, NJ) intravenous constant infusion while subjects are in the MRI scanner. Subjects will receive ketamine (0.23 mg/kg bolus over 1 minute). Because this protocol is designed to specifically evaluate POMA effects on ketamine-induced glutamate activity, the study does not employ a placebo for the ketamine challenge (only for POMA exposure). Total ketamine dose will be limited to 20 mg per infusion.

Studies will be performed in the presence of an Advanced Cardiac Life Support (ACLS)-certified MD or RN, and subjects will be under constant monitoring by ECG and pulse oximeter, as well as by frequent (every 5 minutes) blood pressure measurements. An MD will be present for the duration of the ketamine infusion. To reduce the risk of vomiting in the scanner, subjects will be queried about nausea prior to entering the scanner and immediately prior to the ketamine infusion. Subjects, while in the MRI scanner, will have constant communication access with staff through a sound pipe as well as through an emergency “squeeze bulb” communication system. Participants will be evaluated by a study physician to ensure that the effects of the ketamine have subsided and the subject is stable and has returned to their baseline mental and physical status prior to their discharge. If needed they will be admitted to a hospital research unit for care until cleared for discharge home.

### Ketamine ratings

Subjects will be rated with the Brief Psychiatric Rating Scale (BPRS) (38) and Clinician Administered Dissociative Symptom Scale (CADSS) (6) instruments at baseline and immediately after the scanning period. The site clinical rater staff will administer these measures. See Section 7.2. for details on these rating measures.

### Plasma ketamine/norketamine levels

Venous blood samples (10 mL per blood draw) will be drawn after the ketamine infusion for assay at the Analytical Psychopharmacology laboratory.

### RUCDR genetic collection

On the day of the 1^st^ POMA dose, 22 mL of whole blood will be collected and submitted to RUCDR for potential future genetic analyses. All sites have experience in the collection, labeling and transmission of data for biomaterials and genetics, and have previously worked directly with the NIMH Center for Collaborative Genetic Studies and the Rutgers University Cell and DNA Repository (RUCDR) in the collection, labeling and storing of data for biomaterials. Following RUCDR procedures, we plan to ship out samples on the day of collection, but will store at room temperature if there are delays in shipment.

## PharmacoBOLD Methods

PharmacoBOLD data will be analyzed for ketamine-induced changes in resting state in the medial prefrontal cortex (anterior cingulate cortex). The pharmacological BOLD response (pharmacoBOLD MRI) to ketamine infusion is assessed during rest immediately before (15 minutes) and after (15 minutes) compound administration (39). The data is acquired using a standard echo planar imaging sequence. The acquisition parameters are as follows: repetition time of 2 seconds, 34 4 mm slices, in-plane resolution of 3.4 x 3.4 mm, 300 volumes acquired before and after the bolus. Images preprocessing steps include slice timing correction, realignment, and normalization to a standard space MNI template using FSL. Raw signal time series within each region of interest are smoothed using a loess spline and a scalar is calculated to align the pre- and post-bolus time series based on signal intensity at the end of the pre-bolus and start of the post-bolus scans. The pre-bolus scan is used to estimate a baseline reference value, which can be subsequently used to normalize the post-bolus time series prior to pharmacological model fitting. The temporal phMRI response, a low-frequency signal, is quantified in the general linear model by constructing a regressor that has been previously demonstrated to accurately model ketamine effects on the brain (28, 40). The pharmacological term is constructed to be zero before the bolus infusion and then can be described by an empirical function reaching a maximum at tmax and a shape parameter b: (t/tmax)^(b tmax) exp[(tmax -t)b] after bolus onset. Typical values are b = .01 and tmax = 240 seconds. In additional to the pharmacological regressor, the full model also includes a linear term describing scanner drift and one regressor describing subject motion (41). The subject motion regressor is obtained by taking the first singular value decomposition component from 6 motion parameters (x, y, and z translations and rotations). Summary measures that reflect the amplitude of the pharmacological response for each subject are generated. Data analysis focuses on regions of interest in anterior cingulate cortex. Cross-site calibration will be conducted during the pilot, with the requirement for scans to be within the 95% confidence interval of biomarker validation. All images will be analyzed centrally by the coordinating center.

## End of Participation and Follow-Up

The site study physician will call subjects one day, one week, and one month after their last ketamine infusion in order to check in on their general medical status and to ensure that the subjects are experiencing no sequelae from the ketamine infusion or procedures. In the case that there is some residual or after effect(s), the study physician may ask the subject to return to the study center for medical screening. Subjects will also be informed should clinical reading of their MRI scans show unanticipated abnormalities that require medical follow-up, or if there are other unexpected findings in their routine laboratory assessments. Otherwise, no research information will be shared with subjects.

## Early Termination

Subjects who do not complete the study will be asked to immediately return any unused study drug, and will also be called one day, one week, and one month after termination in order to check in on their medical status.

# STORAGE OF DATA AND SAMPLES

The Research Foundation for Mental Hygiene (RFMH) at Nathan S. Kline Institute for Psychiatric Research (NKI) Data Management Center (DMC) will conduct the data management and data quality assurance. The RFMH/NKI DMC will develop all study Case Report Forms (CRFs) to standardize data collection. A comprehensive web-based data acquisition and management system (Acquire Electronic Data Capture system) will be developed to process, edit, and store all study data in a centralized database. The DMC will review and monitor the completeness and accuracy of data throughout the duration of the study. Final data cleanup will be completed shortly after the last subject visit and the study database will be locked and provided to the study statistician for analyses.

## Submission of Data to NIMH Data Archives

Submission to NIMH National Database for Clinical Trials (NDCT) will be conducted as detailed in Section 19.3.2.

# ADDITIONAL CONSIDERATIONS

## Research with Investigational Drugs

RFMH will obtain an IND from the FDA for POMA and ketamine administration to healthy control subjects before engaging in any study procedures. An academic IND will be obtained, cross-referencing safety data from Lilly and Denovo.

# OUTCOME MEASURES

## Primary Outcome Measures

The primary outcome measure is PharmacoBOLD change, as summarized by the Amplitude (AMP) measure (41). See Section 8.2. for details.

## Secondary Outcome and Safety Measures

The following clinical rating scales assessments and rating scales will be utilized as secondary outcome and Safety measures:

**Outcome Measures**

- 1. **Brief Psychiatric Rating Scale (BPRS)** – The BPRS is a clinician administered rating scale of 18 items that assesses common psychiatric symptoms such as depression, anxiety, and psychotic symptoms. It takes approximately 20-30 minutes to administer (38).

1. **Clinician Administered Dissociative States Scale (CADSS)** – The CADSS rates dissociative symptoms with a 27-item scale, rated 0-4, 19 subjective, 8 observer items and takes approximately 5 minutes to administer (6).

**Safety Measures**

- 1. **Structured Clinical Interview for DSM V Axis I Disorders (SCID)** – The SCID is a standard, clinician administered, diagnostic interview based on the DSM. It takes approximately 30-45 minutes to administer to a healthy control subject, and is used during screening to exclude individuals with Axis I disorders from the study.

1. **Systemic Assessment for Treatment Emergent Events (SAFTEE)** – The SAFTEE monitors common side effects of psychotropic medications on a 0-4 scale (37). It is administered in 5 minutes.
2. **Columbia Suicide Severity Rating Scale (C-SSRS)** – FDA approved scale for the assessment of suicidal ideation and behavior (<http://www.fda.gov/downloads/Drugs/.../Guidances/UCM225130.pdf>)

# STATISTICAL ANALYSIS

## Analysis of Data/Study Outcomes

This study will randomize up to 100 healthy subjects for 81 completers across 4 sites (20-21 completers per site) in a 3-arm randomized (1:1:1) double blind administration of 1) high dose POMA, 2) low dose POMA, or 3) placebo. Assessments will be done using PharmacoBOLD on two days: Screening (Ketamine Screening Visit 2) and Day 10 to test sustained exposure to high or low dose POMA or placebo after ketamine administration. Before the specific statistical techniques are applied, the data management group will examine all variables at all time points for illegitimate values, outliers, and inconsistencies. These values will be queried prior to database lock and corrected, censored, or treated as missing, as appropriate based on the query. Note, data analysis will not include the 6 subjects scanned under the previously approved (Version 4.0) infusion rate. These subjects will be excluded and replaced. Our initial power analysis (protocol section 8.3) was based on 81 completers out of 100 randomized subjects. The excluded subjects will be counted as randomized subjects, allowing us 94 more randomizations to reach 81 completers. We therefore plan to retain our initial analysis plan, and initial power analysis based on 81 completers.

The distribution of demographic variables and baseline clinical characteristics will be examined and described in terms of means, standard deviations, minima, and maxima for continuous variables, and proportions for categorical variables. The complete block design of each arm being administered at each site allows for direct control of difference due to different scanner brands and slight variations in protocols across the sites. All analyses will be intent-to-treat based upon the entire randomized sample, regardless of adherence, which will include up to 100 subjects. Tests will be two-sided and p-values of < 0.05 will be considered statistically significant.

## Analyses by Specific Aims

### Primary Aim

#### Specific Aim 1

*Assess sustained (10 days of exposure) target engagement of high and low doses of POMA vs. placebo.*

Target engagement will be assessed by ability to inhibit the “glutamate surge (Glu)” from acute ketamine administration using PharmacoBOLD as summarized by the change in Amplitude (AMP) measure (41) from baseline (Ketamine Screening) to Day 10 of sustained exposure. Primary hypothesis: Sustained (10 days of exposure) high dose POMA will lead to statistically significant lower increases (i.e., negative change from baseline) in AMP after ketamine administration in anterior cingulate cortex as compared to placebo. Secondary hypothesis: Increases in AMP after ketamine administration in the low dose POMA group will fall in-between placebo and high dose POMA group. Anterior cingulate cortex boundaries will be defined centrally post scan, but pre-database lock.

To evaluate the treatment effect of POMA on ketamine-induced BOLD signals in pre-specified ROIs (ACC and anterior insula) in resting fMRI, analysis of covariance (ANCOVA) with change from baseline (Ketamine screening) to Day 10 in Amplitude (AMP) as the outcome predicted by baseline (Ketamine screening) AMP and treatment in 3-treatment groups (high dose POMA group, low dose POMA group, and placebo group) will be performed, and the mean and 95% confidence interval (CI) will be estimated. Tests of treatment effect will be made within each treatment group, between each active treatment group (high dose POMA group and low dose POMA group) versus placebo group, and also between the two active treatment groups from the ANCOVA. A statistically significant negative change of AMP found in the active treatment groups compared to placebo, and a significant negative change of AMP in high dose as compared to low dose group would be consistent with the hypotheses. Cohen’s d effect sizes will be reported using the standard deviation of baseline BOLD. Differences in effects across site will be tested via a group by site interaction and if found to be significant, results will be additionally reported separately by site.

### Secondary Aims

*Behavioral and safety measures:* Secondary clinical and safety outcome measures (BPRS, CADSS, SAFTEE) will be analyzed similarly to the primary outcome of PharmacoBOLD. Clinical measures will be assessed after ketamine administration. The following contrasts will be tested: 1) high dose POMA versus placebo, 2) low dose POMA versus placebo, and 3) low dose POMA versus high dose POMA. Primary hypothesis: High dose POMA will lead to a statistically significant change from baseline (Ketamine screening) to Day 10 in behavioral measures after ketamine administration compared to placebo. Secondary hypothesis: The change in behavioral measures after ketamine administration in the Low dose POMA group will fall in-between placebo and high dose POMA group.

## Power Analysis

*Specific Aim 1:* This study is powered for the primary outcome. We based our power analysis on an effect size of 0.8 found in a prior similar study, at Eli Lilly (Adam Schwarz, personal communication), between POMA (80 mg BID) versus placebo. In the present study, the low and high dose POMA are half and twice as large as the prior study. Hence, assuming conservatively there is no increased dose response relationship, such that the effect size in the high dose is also 0.8, we have >80% (specifically 89.5%) power to detect a significant difference from placebo in the high dose POMA. The low dose POMA is expected to achieve a smaller effect size, and although we cannot make firm assumptions about what the expected effect size will be, compared to either the high dose POMA or placebo, nevertheless, the smallest effect size in the low dose group that would be detectable in a test versus placebo or high dose POMA with 80% power is 0.69 and with 70% is 0.60. Power analysis is based on 81 completers. In order to achieve 81 completers, we anticipate randomizing up to 100 subjects, replacing post Day 1 dropouts with an equivalent arm assignment.

# HUMAN SUBJECTS PROTECTION

## Human Subjects Involvement, Characteristics, and Design

During this project, we will randomize up to 100 healthy subjects to obtain 81 completers across the 4 sites. We will include subjects of both genders in approximately equal numbers. The overall ethnic composition of the group will reflect the ethnic composition of previous studies we have completed using a healthy adult population (42% Caucasian, 22% Hispanic, 24% African-American, 12% Asian or other). Approval from the central Institutional Review Board (IRB) will be obtained for Columbia, UCLA and UAB, along with local IRB approval for NYU. All staff members involved in this project are required to receive training in the protection of human subjects. Subjects will be recruited through advertisements in newspapers, flyers, and the Internet using advertisements approved by the relevant IRBs.

All subjects will receive medical and psychiatric screening to confirm eligibility. Eligible subjects will complete two experimental sessions consisting of PharmacoBOLD procedures. All subjects will receive 10 days of study drug (randomized in a 1:1:1 ratio). All work with human subjects will be conducted by the Principal Investigators or their study physicians and staff at their respective sites. Only subjects who are considered capable of providing informed consent will be included. Consents will be obtained in accordance with the respective state guidelines. No waiver of informed consent for participation in this study will be requested. Because of the potential burden to subjects, subjects will be compensated for this study (Section 23.). Special vulnerable populations such as fetuses, neonates, pregnant women, prisoners, and institutionalized individuals will not be included in this study. Children over the age of 18 will be included (see Section 12.2.). The screening methods, inclusion and exclusion criteria, their rationale, and potential risks are described in Sections 9.1.1. and 9.1.2.

### Screening Methods

After providing informed consent, subjects will have full medical screening (i.e., medical history, physical examination, laboratories for basic chemistries, blood counts, liver function tests, urinalysis, urine toxicology, serum pregnancy test for women, and thyroid tests, electrocardiogram [EKG]) and psychiatric screening (i.e., Structured Clinical Interview for DSM V Axis I Disorders [SCID]) to confirm eligibility.

### Inclusion/Exclusion Criteria Rationale

- 1. *Exclusion of Individuals with Significant Medical or Neurological Illness including high blood pressure, low blood pressure, seizure or cardiac illness*: These individuals were excluded to minimize the potential medical risks from receiving ketamine and POMA.
  2. *Exclusion of Individuals with History of Significant Violent Behavior*: These individuals were excluded to minimize the potential psychiatric risks from receiving ketamine.
  3. *Exclusion of Individuals with Metallic Objects in Body or Claustrophobia*: These individuals will be excluded to decrease potential risks from MR scanning.
  4. *Exclusion of Individuals with body weight greater than 86.95 kg:* Weight limit of 86.95 kg is to allow for a <20 mg ketamine infusion under the FDA limits.
  5. *Exclusion of subjects with subthreshold pharmacoBOLD response.* This is to ensure only ketamine responders will be entered.

# QUALIFICATIONS OF INVESTIGATORS

## Principal Investigator

*Jeffrey Lieberman, M.D.*, is the Director of NYSPI and Chair of the Department of Psychiatry at Columbia University (CU), and will serve as overall Principal Investigator. Dr. Lieberman served as overall Principal Investigator for the recently completed FAST-PS Biomarker Validation study and thus has the breadth and experience required to efficiently oversee this multi-site clinical trial study.

## Site Principal Investigators

*Daniel Javitt, M.D., Ph.D.,* is the Director of the Division of Experimental Therapeutics at CU and Director of Schizophrenia Research at NKI, and will serve as site-PI of the CU site. Dr. Javitt served as a site PI for the preceding FAST-PS Biomarker Validation study and thus has the breadth and experience required to efficiently conduct this multi-site clinical trial study.

*Adrienne Lahti, M.D.,* is the Patrick H. Linton Professor of Psychiatry at the University of Alabama at Birmingham (UAB) and the Director of the Division of Behavioral Neurobiology and the Co-Director, Alabama Advanced Imaging Consortium, and will serve as the site-PI for the UAB site. She has extensive experience in the Use of multimodal brain imaging techniques (PET, fMRI, MR Spectroscopy) to study the neuropathology of schizophrenia and to evaluate the effects of psychotropic drugs on brain function and biochemistry. Dr. Lahti therefore has the breadth and experience required to efficiently conduct this multi-site clinical trial study.

*Stephen Marder, M.D.,* is a Professor and the Director of Section on Psychosis in the Department of Psychiatry at University of California Los Angeles (UCLA), and will serve as the site-PI for the UCLA site. Dr. Marder and UCLA were the PI and primary site for TURNS, and Dr. Marder was the PI for the MATRICS contract and the Director of a VA Mental Illness Research Education and Clinical Center (MIRECC) that supports research, educational, and clinical activities throughout all of Southern California. As such, Dr. Marder and UCLA have the experience and expertise to conduct this multi-site clinical trial study. Dr. Marder also served on the Advisory Board of PORT, and has published some of the most seminal papers in the psychopharmacologic treatment of schizophrenia.

*Donald Goff, M.D.,* is the Vice Chair for Research in the Department of Psychiatry at the New York University Langone Medical Center (NYULMC) and the Director of the Nathan Kline Institute for Psychiatric Research (NKI), and will serve as the site-PI for the NYU site. Dr. Goff was a site-PI in the TURNS initiative and has spent his career using clinical trials and brain imaging to development experimental medications for schizophrenia. In addition, Dr. Goff’s position as Vice-Chair at NYULMC and Director of the Nathan Kline Institute will ensure that this study can utilize the great resources available at NYULMC.

## Data Management Center

*James Robinson, M. Ed.,* is the Director of the Information Sciences Division for the Research Foundation for Mental Hygiene at the Nathan S. Kline Institute and the Director of Clinical Research Informatics and Data Management at the Center for Health Informatics and Bioinformatics, New York University Langone Medical Center, and will serve as the Director of the Data Management Center. Mr. Robinson is uniquely qualified to lead the clinical research informatics and data management for this study. He possesses all of the requisite skills and expertise. Mr. Robinson has spent his entire career providing clinical research support services. He has over thirty-five years of experience in clinical research data management, informatics, quality assurance, and study monitoring. Mr. Robinson is currently the Director of Data Management Centers for five multisite clinical research studies including the 35-site Feinstein Recovery after Initial Schizophrenic Episode (RAISE) study funded by the National Institute of Mental Health.

# ANTICIPATED BENEFIT

The potential direct benefits are that participants will receive medical and psychiatric assessments by skilled, experienced clinicians. The indirect benefits are more considerable; however, as we believe that this study will help us determine the feasibility of continuing to develop medications that target metabotropic glutamate receptors for schizophrenia. This indirect benefit may lead to the more effective development of novel therapeutic agents and therefore have great implications for society. Therefore, we believe the mild risks of this study are far outweighed by the potential benefits to patients suffering from schizophrenia. The medications currently available for schizophrenia, while very effective, have well-known drawbacks, such as their limited efficacy against negative symptoms and cognitive deficits, as well as their side effects. Therefore, it is warranted to pursue every possible lead to develop a better understanding of treatment effects on the brain that will in turn help us to more effectively develop agents that can help to treat persistent symptoms while minimizing side effects.

While financial compensation will be provided for the time and effort that subjects provide, this is not considered a benefit of study participation.

# RISKS AND DISCOMFORTS

## Potential Risks and Protections Against Risks

Risks associated with the study are related to: a) POMA administration; b) Ketamine administration; c) MRI (PharmacoBOLD) scan; d) venous blood sampling; e) intravenous catheter; and f) interviews and clinical assessments.

### Risks and Discomforts Associated with POMA Administration

In clinical studies as of 31 January 2012, POMA has been given to 460 healthy people and approximately 2355 patients with schizophrenia or schizoaffective disorder.

The most common AEs (≥10%) in clinical pharmacology studies conducted in healthy subjects were: nausea, headache, vomiting, and dizziness. The following SAEs have been reported in healthy subjects: infected sebaceous cyst (1), deep vein thrombosis (1), pulmonary embolism (2), and syncope due to vasovagal event (1). With the exception of syncope due to vasovagal event, none of these SAEs were reported by the investigator as related to administration of POMA.

In less than 1% of patients with schizophrenia treated with POMA, events of uncontrollable and rapid shaking (convulsions or possible seizures) have been reported. These convulsions appear to have been associated with POMA. This risk may be five times higher than the risk of seizures with other antipsychotics.

**Table 6** provides a summary of treatment emergent adverse events (TEAEs), which occurred in ≥2% of schizophrenia patients who were assigned to take POMA in completed clinical studies (6-8 weeks). For the TEAEs displayed in the table, a comparison with patients assigned to take placebo is shown. **Table 7** provides a summary of TEAEs which occurred in ≥2% of patients who were assigned to take POMA in the long-term (~1 year) open label completed clinical study. For the TEAEs displayed in the table, a comparison with patients assigned to standard-of-care (SOC) treatment is shown.

In the 6-8 week study (26), no significant treatment-emergent dyskinesia, akathisia, or parkinsonism effects were observed. Furthermore, changes from baseline in Barnes Akathisia Scale (BAS) showed statistically significant symptom reduction in the POMA group compared to placebo after 4 weeks of treatment. The Simpson-Angus Scale (SAS) and Abnormal Involuntary Movement Scale (AIMS) Items 1-7 showed no significant difference between treatment groups.

Similarly, the results of the analysis of treatment-emergent EPS symptoms in (27) showed no statistically significant treatment group differences. On the SAS and on the BAS Global score, statistically significant baseline-to-endpoint within-treatment group improvement was observed at Week 4 for all treatment groups.

In the completed long-term open label study, the following TEAEs occurred with an incidence of ≥2% of patients who received POMA and with a statistically greater frequency among POMA-treated patients compared with SOC-treated patients: insomnia, vomiting, agitation, nausea, dyspepsia, bronchitis, and weight decreased (**Table 7:** Table adapted from Investigator Brochure). Other TEAEs which occurred with greater frequency among POMA -treated patients (incidence ≥5%, but not statistically significant compared to SOC) included: anxiety, headache, schizophrenia, increased creatine phosphokinase, and nasopharyngitis.

**Table 6** Treatment-Emergent Adverse Events (≥2% in POMA) in Completed Clinical Studies in Patients with Schizophrenia who were Assigned to Take POMA or Placebo

| **MedDRA Preferred Term** | **POMA**  **(N = 583)** | | **Placebo**  **(N = 185)** | |
| --- | --- | --- | --- | --- |
|  | N | % | N | % |
| Insomnia | 85 | 14.1 | 20 | 10.8 |
| Eosinophilia (including eosinophil count and eosinophil count increased) | 71 | 12.2 | 7 | 3.8 |
| Anxiety | 36 | 6.2 | 11 | 5.9 |
| Nausea | 27 | 4.6 | 5 | 2.7 |
| Headache | 23 | 3.9 | 4 | 2.2 |
| Agitation | 22 | 3.8 | 9 | 4.9 |
| Vomiting | 17 | 2.9 | 1 | 0.5 |
| Blood creatine phosphokinase increased | 16 | 2.7 | 3 | 1.6 |
| Schizophrenia (exacerbation) | 15 | 2.6 | 3 | 1.6 |

Abbreviations: MedDRA = Medical Dictionary for Regulatory Activities; N = total number of patients in Studies HBBD and HBBI in each treatment group; n = number of patients who experience treatment-emergent adverse event.

Source: H8Y-MC-HBBI Abbreviated Study Report (Table HBBI.11.29); H8Y-BD-HBBD Abbreviated Study Report (Appendix 11.2.2).

Table adapted from Investigator Brochure.


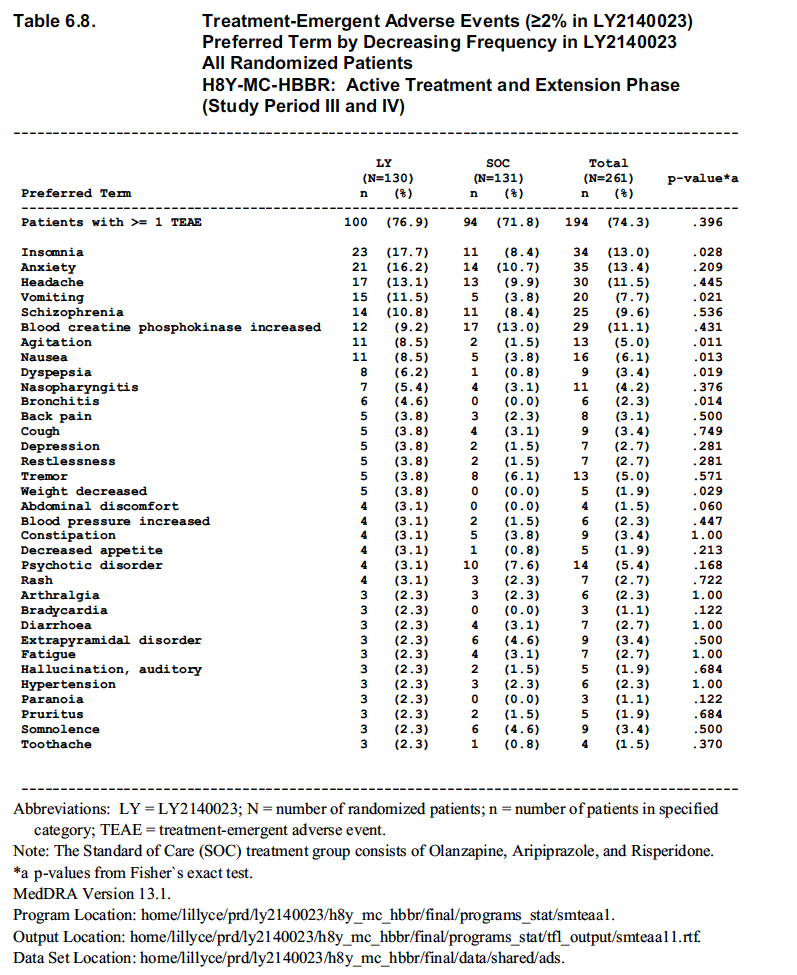


Table 7.

In other studies, people who took compounds similar to POMA have experienced rash, skin itching, or skin peeling. Because of these experiences, rash, itching of the skin or skin peeling will be closely monitored. Subjects will be asked at every visit about these skin conditions.

Of the 762 patients diagnosed with schizophrenia who took POMA in clinical trials, 52 patients stopped taking part in the studies because of uncontrollable and rapid shaking (possible seizures), nervousness, restlessness, inability to sleep, worsening of schizophrenia symptoms, chest pain, vomiting, stomach pain, soft or watery stools, rash, abnormal heart function, feeling anxious or apprehensive, suicidal thoughts, excessive excitement, high blood pressure, ulcer in the small intestine, pneumonia, acting angry or aggressive, or increase of muscle enzymes in the blood.

These potential side effects will be monitored for using the SAFTEE and scheduled laboratory tests. Any noted side effects will be evaluated by the site study physician.

#### Deaths

There were 12 deaths reported in completed studies and ongoing studies. In these 12 death cases, 8 patients were assigned to take POMA, 1 patient was assigned to take placebo, 1 patient did not take any study drug, and 2 patients received study medication that remains blinded at the time of this report. Also, of these 12 death cases, 6 were completed suicides with 4 of those patients assigned to take POMA. Of the 8 patients assigned to take POMA, 4 of those deaths occurred after the study drug had been held or discontinued. Also, of the 8 patients assigned to take POMA, 5 of those deaths were considered to be not related to the study drug treatment by the investigator while all 8 of those deaths were considered to be not related to the study drug treatment by the sponsor. A listing of death cases for completed and ongoing studies is presented in Table 6.1 in the investigators brochure and described below for seven cases. Full details are unavailable for five deaths.

A death due to congestive cardiomyopathy was reported 10 hours after a patient with schizophrenia received the first dose of randomized placebo treatment. Laboratory findings revealed after the death included elevated creatine kinase, white blood cells, and absolute neutrophils. The autopsy findings included a concomitant disease of cancer of the right lung, with the final report indicating the cause of death as dilation cardiomyopathy with acute cardiac insufficiency. The investigator indicated that the event was not related to study drug administration or study procedures.

A death due to asphyxia was reported. A patient with schizophrenia was discontinued from study drug due to agitation after 8 days of 40 mg BID POMA treatment. The patient subsequently received oral risperidone, with the severity of agitation lessening but not resolved prior to patient’s death, per the coinvestigator. The patient began receiving trihexyphenidyl hydrochloride and lorazepam after extrapyramidal side effects reappeared. While in the hospital, the patient choked on food during dinner and, despite resuscitation attempts, died from accidental asphyxia. The patient’s mental status at the time prior to death was unknown. The investigator indicated that the event was not related to study drug administration or study procedures.

A death due to completed suicide was reported in a 28-year-old male patient with schizophrenia. Past medical history was significant for a diagnosis of paranoid schizophrenia, a questionable history of previous suicide attempt, and cannabis use. The patient had no suicidal ideation or plans at the time of screening for study participation. On the seventh day of study drug administration (POMA 80 mg BID), the patient was noted to be anxious, insecure, with paranoid delusions and guilt delusions, but with no evidence of suicidal intent. On the following day, his mood was noted to have improved. He had left the hospital ward in the morning and later that afternoon committed suicide by jumping in front of a commuter train. The investigator indicated that the event was not related to study drug administration or to study procedures.

A death due to ischemic cardiomyopathy was reported in a 46-year-old male patient with schizophrenia. Past medical history was significant for smoking (30-40 cigarettes/day for 28 years) and social drinking. He was not obese and had no family history of cardiac disease or diabetes mellitus. The patient had no complaints of weakness or chest pain at the time of study entry. On the third day of study drug administration (POMA 80 mg BID), the patient was discontinued from the study due to lack of efficacy manifested as increased anxiety, tension, and restlessness. Two weeks after being discontinued from the study, the patient experienced weakness, dizziness, anxiety, restlessness, and reduced blood pressure (90/50 mmHg). Approximately four weeks after he was discontinued from the study, his condition worsened. The patient had fallen and suffered a facial injury. He also had symptoms of bronchitis and complained of weakness and chest pain. Later that night, he fell again, at which time his blood pressure was 95/60 mmHg. He died that same night. Autopsy revealed ischemic cardiomyopathy and acute cardiac failure. The investigator indicated that the death was not related to study drug administration or study procedures.

One death due to pneumonia secondary to surgical complications was reported in a 41-year-old male patient with schizophrenia administered POMA 80 mg BID. The subject received intramuscular treatment with diclofenac on several occasions for neuralgia and was instructed to take additional non-steroidal anti-inflammatory drugs, if needed, for pain and, according to the subject’s family, he took uncontrolled amounts of diclofenac about 10 tablets daily (25 mg). In the opinion of the study investigator, the events of duodenal ulcer and pneumonia with the outcome of death were not related to study drug administration or study procedure; the subject had taken diclofenac several times.

One death due to acute renal insufficiency was reported in a 37-year-old male patient with schizophrenia administered POMA 40 mg BID. The patient died approximately one week after discontinuing treatment with POMA. In the opinion of the study investigator, the events of chronic renal failure, brain edema, acute renal insufficiency, acute hepatic insufficiency, acute viral respiratory infection, and suspicion of pneumonia were not related to study drug administration. Additional information following medical review included the investigator's opinion of cause of death as "patient probably suffered from acute viral infection that eventually worsened to cause hypotensive shock (possibly due to shock-related gastrointestinal ulceration), acute renal failure, acute hepatic failure, and ultimately death.”

One death due to possible suicide was reported in a 54-year-old female patient with schizophrenia (patient had not taken any study drug). In the opinion of the study investigator, the event of death (possible suicide) was not related to study drug administration or study procedure; the subject had not received any dose of study drug or placebo.

### Risks and Discomforts Associated with Compound LY404039 in Humans

LY2140023 monohydrate (LY2140023) is the methionine prodrug of the mGlu2/3receptor (mGlu2/3R) agonist, LY404039. LY404039 has been given to 24 healthy people in a previous study. Experiences reported by 2 or more people who have taken LY404039 were headache, feeling dizzy upon standing up, stomach pain, nausea (feeling sick to the stomach), sore throat, tiredness, or feeling sleepy.

### Risks and Discomforts Associated with POMA in Animals

POMA has been studied in animals. Rats treated with POMA showed uncoordinated movement, problems with walking, reduced body weight, uncontrollable and rapid shaking (convulsions or possible seizures), or death. In one out of 40 rats treated with very high doses of POMA for 6 months, areas of brain injury were observed. Mice treated with POMA also showed uncontrollable and rapid shaking (convulsions or possible seizures). Vomiting, soft or watery stools, and/or a severe rash were seen in monkeys given high doses of POMA for up to one year. A single monkey was euthanized due to severe skin rash on Day 235 of the 1-year study. No convulsions have been observed in monkeys. In a study of the effects of POMA on pregnant rats and their offspring, some baby rats had significant decreases in their body weight, and some baby rats died up to 14 days after birth. The levels of drug in subject’s blood will be lower than the levels of drug in monkeys at which the above bad effects were noted. Effects of convulsion observed in rats and mice were observed at blood drug levels that are similar to or lower than the levels expected in this study.

### Ketamine Administration

#### Medical Risks

Studies will be performed in the presence of an Advanced Cardiac Life Support (ACLS)-certified MD or RN, and subjects will be under constant monitoring by ECG and pulse oximeter, as well as by frequent (every 5 minutes) blood pressure measurements. An MD will be present for the duration of the ketamine infusion. To reduce the risk of vomiting in the scanner, subjects will be queried about nausea prior to entering the scanner and immediately prior to the ketamine infusion. Subjects will as usual in the MRI scanner have constant communication access to staff through a sound pipe as well as through an emergency “squeeze bulb” communication system.

Administration of subanesthetic ketamine i.v. induces a modest rise in blood pressure and pulse. The study investigators have administered subanesthetic doses of intravenous ketamine in the setting of numerous published brain imaging studies with IRB approval. Dr. Lawrence Kegeles, a co-investigator at the RFMH-NYSPI site, has published data from studies utilizing ketamine doses similar to that employed in this study. In the first study, ketamine was administered at 0.2 mg/kg intravenous bolus plus 0.4 mg/kg/hr for 4 hours, for a total dose of 1.8 mg/kg (n=8 healthy volunteers) (42). In the second study, ketamine was given as an intravenous bolus (30 sec) at a dose of 0.12 mg/kg, followed by a constant infusion over the next hour of another 0.65 mg/kg, for a total dose of 0.77 mg/kg (n= 10 healthy volunteers) (31). In the first study, the resulting effects on vital signs for a group of 6 healthy volunteers are presented as a function of time in **Table 8** below for the first 50 minutes post-injection. These modest increases all peaked and largely resolved by 50 minutes, with vitals returning to near baseline, at which time subjects were mobilized for the first scan. Administration of subanesthetic doses of ketamine can induce nausea and vomiting. Of the six subjects whose vital sign data are presented below, one withdrew because of nausea and vomiting, and another suffered from these symptoms. In a more recent, lower-dose ketamine administration protocol identical to the one utilized in this study, all subjects have been free of nausea and vomiting.

| **Table 8.** Effects of ketamine on systolic and diastolic blood pressure in a group of normal subjects (n=6). | | | |
| --- | --- | --- | --- |
| **Time**  **(min)** | **Systolic Blood**  **Pressure after** **Ketamine** | **Diastolic Blood**  **Pressure after** **Ketamine** | **Pulse after**  **Ketamine** |
|  | (mm Hg) | (mm Hg) | **(min-1)** |
| -2.5 | 115.5 | 70.5 | 71.5 |
| 0.0 | 146.0 | 81.8 | 99.8 |
| 5.0 | 150.8 | 87.8 | 92.2 |
| 10.0 | 150.6 | 94.6 | 89.6 |
| 15.0 | 142.0 | 81.3 | 82.5 |
| 20.0 | 146.8 | 80.8 | 73.8 |
| 25.0 | 142.0 | 67.0 | 86.0 |
| 30.0 | 153.3 | 85.0 | 88.3 |
| 40.0 | 133.0 | 77.3 | 80.3 |
| 50.0 | 125.0 | 72.7 | 78.3 |

These adverse effects will be minimized by excluding participants with a history of cardiac illness, by conducting a baseline physical examination to evaluate possible hypertension, by obtaining a baseline EKG, and by monitoring EKG, pulse, oxygenation, and blood pressure during ketamine administration.

#### Psychiatric or Behavioral Risks

Ketamine is an FDA-approved dissociative anesthetic. Ketamine exposure at the subanesthetic dose to be used in this study can be associated with a moderate dissociative state, which is well tolerated in the majority of cases and spontaneously reversible (6). There is extensive clinical experience with ketamine used at anesthetic doses, and no long-term detrimental effects of ketamine exposure have been reported. It is possible that ketamine administration will increase the risk of psychosis, even in normal subjects. Ketamine is a street drug of abuse, sometimes called ‘special k.’ As such; it poses the risk that exposure during this study may predispose subjects to subsequent abuse of this drug.

Medical and psychiatric screening will minimize initial risk during the screening/consent procedure as described above. The experiment will be carried out in the presence of at least one psychiatrist and an ACLS-certified physician or RN. Medical risk will be monitored and minimized by use of continual EKG, heart rate and pulse oximetry monitoring as well as frequent blood pressure readings. The risks of exposing healthy subjects to a drug of abuse potential will be minimized by explaining these risks to prospective subjects, and by excluding from the study any subjects with documented or suspected prior substance or alcohol abuse history. In addition, all subjects will, if not cleared for discharge by a study physician the afternoon after the study, be admitted to the research unit at the respective site if needed for observation and supportive treatment. If admitted, subjects will undergo assessment for clearance for discharge, at the earliest the morning following the study. Subjects will be followed up by telephone by research staff one day, one week and one month later, for any possible adverse outcomes related to administration of ketamine. Finally, the Data and Safety Monitoring Plan and Board will have as a central focus the risks and protections against risk of administration of ketamine (see Section 15. Data and Safety Monitoring).

Additionally, the study investigators have prior experience with multiple i.v. infusions of subanesthetic doses of ketamine and demonstrated its safety, as well as its lack of additional effect on neurochemical response (31). In addition, serial ketamine infusions have been used without adverse effects in multiple depression studies (43, 44). Thus, our plan to have up to 2 infusions per subject, have documented safety.

### MRI (PharmacoBOLD) Scans

All MRI scans will occur on 3T scanners at each site.

#### MRI Safety: 3T system

There are no known long-term biological risks from the use of MRI scanners per se, including the 3T scanners for use in this proposal. The 3T systems proposed in this study are nonsignificant risk devices (per FDA website, U.S. Food and Drug Administration, Center for Devices and Radiological Health, "Guidance for the Submission of Premarket Notifications for Magnetic Resonance Diagnostic Devices", <http://www.fda.gov/cdrh/ode/95.html>, Nov., 1998). Human studies have been carried out under IRB approval in the US since 1990 under this same evaluation at a variety of institutions throughout the US. More recently, field strengths of 7T and 8T have been approved for research studies in humans at the University of Minnesota and Ohio State University. All MRI studies follow guidelines set by the FDA with regard to specific absorption ratio (SAR), limits on gradient slew rate (dB/dt), and noise.

At the same time, possible risks associated with MRI scanning can be classified into one of six areas: a) Acoustic Noise Levels; b) physical discomfort; c) fetal exposure; d) static magnetic fields (leading to the attraction of ferromagnetic metal objects); e) Gradient or Time-Varying Magnetic Fields (leading to the possible stimulation of peripheral nerves); and f) Radiofrequency (RF) Magnetic Fields (leading to the possible risk of tissue heating).

1. *Acoustic Noise Levels*: The acoustic noise associated with MR imaging is related to the mechanical movement of the gradient coils during the scanning process.
   *FDA Guidelines:* "The acoustic noise levels associated with the device must be shown to be below the level of concern established by pertinent Federal Regulatory or other recognized standards setting organizations. If the acoustic noise is not below the level of concern, the sponsor must recommend steps to reduce or alleviate the noise perceived by the patient." Current FDA guidelines follow the regulations of the International Electrotechnical Commission (IEC) Standard 601-2-33, which stipulate that for MR equipment used in medicine, hearing protection is required when the system can produce acoustic sound levels above 99 dBA (maximum A weighted r.m.s.) and that the protection should be able to reduce noise levels to below 99 dBA. The FDA has approved systems for which noise levels have been quantified, ranging up to 105 dB RMS for scanners operating at field strengths of 1.5 Tesla. It is important to note that the static magnetic field strength is only one factor, and not necessarily the most important one, in determining acoustic noise. Among the factors listed above, the design and construction of the gradient coils plays a major role in the noise level that MRI scanning produces. Therefore, noise levels are not necessarily greater when scanning at 3.0 T compared with 1.5 T field strengths. It is nevertheless possible that, in some circumstances, our systems could produce noise levels higher than 99 dB, as do many clinical systems operating at lower field strengths.
   *Summary:* The acoustic noise levels perceived by human subjects when undergoing MRI examination in our 3.0 Tesla magnets constitutes a non-significant risk; specifically, our systems will not be operated in a way that will present more noise to human subjects than is recommended by the FDA.
2. *Physical Discomfort*: The physical confinement and isolation produced by the scanner could cause mild to moderate emotional distress, although subjects generally tolerate the procedures remarkably well.
3. *Fetal Exposure:* The risk of MR imaging to the fetus is unknown.
4. *Static Magnetic Fields*: The possible risks of static magnetic fields have received much attention in the lay press, but scientific consensus on these risks has yet to be fully reached. The FDA has deemed that systems operating at 8.0 Tesla or less do not pose a significant risk. Moreover, experience with thousands of clinical studies over the past decade, and with multiple human investigations carried out at higher field strengths over this period, have not revealed risks of exposure to higher static magnetic fields. The most significant risk associated with static magnetic fields is that ferromagnetic objects, such as aneurysm clips or heart valves, can interact with the magnetic field of an MRI scanner, causing the device to malfunction or to move, and injuring the subject.
   *FDA Guidelines:* “Studies conducted at 8T or less are not considered significant risk" (FDA Center for Devices and Radiological Health, memorandum 7-14-03).
   *Summary:* This category of risk applies to work conducted around superconducting magnets of any kind (including standard clinical diagnostic MRI units). It is not unique to our 3.0 Tesla facilities, which will maintain safety policies to safeguard subjects and staff members from these incidental risks. Systems with static magnetic fields less than 8 Tesla have been considered to represent a nonsignificant risk by the FDA. The static magnetic fields of systems (3.0 Tesla) are therefore to be classified as posing nonsignificant risk to human subjects.
5. *Time-Varying Magnetic Fields*: The concern about the time-varying magnetic fields used in MRI is that these can, in some instances, induce stimulation of peripheral nerves, thereby producing sensations such as 'twitching' or 'tingling'. In very rare instances, this nerve stimulation can be painful. Nerve stimulation is particularly likely when subjects are physically positioned in a way that increases the likelihood of inducing stimulation, such as with hands clasped or arms folded. It should be noted that the parameter of interest here, dB/dt (the rate of change in the magnetic field per unit time), is not a function of the strength of the static magnetic field, so evaluating risk in a 3T MRI scanner involves the same considerations as evaluating other MRI systems operating at lower magnetic field strengths (i.e., the same issues apply to all the commercially available, FDA-approved scanning systems). Thus, it is the *gradient system only* that needs to be evaluated to determine the risk of producing nerve stimulation.
   *FDA Guidelines:* The FDA Guidance of 1995 was developed specifically to consider the fact that many clinical systems were capable of exceeding levels of dB/dt that could produce nerve stimulation. It was originally considered that a warning level should be implemented to guard against peripheral nerve stimulation, but the FDA finally concluded that: *'... this warning level is not considered critical since there are no harmful effects associated with mild peripheral nerve stimulation’.* The current guidelines therefore include monitoring procedures to help avoid painful peripheral nerve stimulation, and without specific dB/dt limitations*.*
   *Summary:* The gradients used in our 3.0 Tesla MRI systems will typically be operated at levels below those considered to be negligible according to FDA guidelines. Our systems, like most commercially available, FDA-approved systems, have the capacity to exceed this level, but we will include the same safeguards that are included in other FDA- approved clinical systems. Furthermore, policies and procedures will be implemented according to FDA guidelines to avoid the possibility of painful peripheral nerve stimulation. Therefore, in all circumstances the system will be operated in a way that poses nonsignificant risk to the participant.
6. *Specific Absorption Rate (SAR):* MRI scanning induces some heating of body tissues. This specific absorption rate (SAR) that determines heating is the amount of radiofrequency (RF) energy deposited (typically by a coil or “helmet”-like apparatus placed over the subject’s head) per unit volume of tissue per unit time. The SAR for RF radiation is primarily related to the amplitude of RF power, duration of the RF pulse, type of RF coil, frequency of RF radiation, resistivity of the tissue, configuration of the anatomical region, and several other parameters.
   *FDA Guidelines:* "The following are levels of concern: A) If SAR ≤ 0.4 W/kg whole body, and if SAR ≤ 8.0 W/kg spatial peak W/kg averaged over the head: **below level of concern,** or B) If exposure to radiofrequency magnetic fields is insufficient to produce a core temperature increase in excess of 1°C and localized heating to greater than 38°C in the head, 39°C in the trunk and 40°C in the extremities: **below level of concern**. The parameter SAR cited above must be shown to fall below either of the two levels of concern by presentation of valid scientific measurement or calculation evidence sufficient to demonstrate that SAR is of no concern."
   This guideline is based on the calculation of a system that has no thermoregulatory response, and thus it is a very conservative estimate compared with the temperature change that would be experienced in any living subject. Normal diurnal temperature variations in humans, for example, are about +/-1°C from the normal set point 37°C, and healthy people with normal thermoregulatory responses can easily dissipate any excess (or, in this instance, deposited) heat by increasing their peripheral blood flow or sweat rate. Thus, the heating effect of MRI with the SARs used in accord with these guidelines is extraordinarily unlikely to cause any acute effects in healthy human subjects. Furthermore, our scanner consoles calculate SAR based on the subject’s body weight before running any pulse sequence and prohibits running of the sequence if exceeds the FDA-approved limit.
   *Summary:* Because all experiments performed on the 3.0 Tesla systems will comply with FDA guidelines with regard to SAR, and because appropriate RF power safety checks are in place, this criterion for classification of NSR is satisfied.

#### Minimizing Specific Risks Posed by MRI scanning

The 3.0 Tesla scanners satisfy FDA criteria for nonsignificant risk in all risk categories. The following steps are taken to minimize risk:

1. Acoustic Noise*:* As suggested by the FDA, we take steps to reduce the noise levels experienced by subjects. The easiest and most reliable means of preventing hearing loss is to use disposable earplugs, which we will do for all scans. We will also be using acoustically shielded headsets, which further attenuate noise.
2. Physical Discomfort: All subjects will be able to communicate directly with technologists and study staff to inform them of any emotional or physical distress during the scanning procedure. If they wish, the scan will be terminated immediately and the subject will be removed from the scanner.
3. Fetal Exposure: While there is no known risk of MR scans to the fetus, it is standard practice to exclude women who are pregnant from research MR scans. Therefore, to implement this exclusion a pregnancy blood test is performed at screening and, in addition, a urine pregnancy test is performed on the day of each MR scan for all female participants.
4. Static Magnetic Fields: These risks are the same as in other commercially available clinical systems. Like clinical MRI centers, our facilities have a complete range of procedures to assure security of the restricted access area, careful screening of potential subjects before they enter the restricted access area and a metal detector positioned at the doorway leading into the magnet room within the MRI suite. In addition, access is tightly controlled, allowing only those personnel and research subjects who have legitimate reason to be there. Doors to the unit will be securely locked, with only MR technologists, physicists, or physicians controlling entry of ferromagnetic and other materials that could possibly cause harm to research subjects, personnel, or equipment. In addition, entry- ways to the unit will be labeled with clear visible signs warning of the presence of the magnetic field and the exclusion from entry by individuals with implanted metal objects such as prostheses, pins, clips, IUD’s, etc.
5. Nerve Stimulation*:* The consent form will provide information about this risk. A record of dB/dt value will also be included with the imaging data to help in analysis of levels of peripheral nerve stimulation possibly perceived by subjects. In addition, we will conduct detailed calculations of the changes in magnetic field over time that our gradient system is capable of, and conservative values will be selected as limits that will be used to determine when special additional monitoring is indicated. In these cases, we will use the monitoring procedures recommended by the FDA. The gradient switching times and strengths will also be monitored together with the routine assessment of all electrical components of the system, as described previously.
   In addition, MR technologists receive special training to prevent peripheral nerve stimulation. Before any scanning procedure that might stimulate peripheral nerves, a technologist will: inform the subject that peripheral nerve stimulation may occur; describe the nature of the sensation to the subject; instruct subjects not to clasp their hands, since this may create a conductive loop which will increase the possibility of stimulation; maintain constant verbal contact with the subject; instruct subjects to inform the MR technologist if they experience discomfort or pain; terminate the scan if the subject complains of discomfort or pain; complete a report of any incidents involving severe discomfort or pain, including describing the associated circumstances (imaging parameters, dB/dt value, level of pain, etc.), and submit this report immediately to the IRB.
6. SAR Absorption: The magnitude of temperature increase during MRI scanning is minimal. Increases are always within FDA guidelines, which include core temperature increases less than 1°C, as well as localized heating to less than 38°C in the head, 39°C in the trunk, and 40°C in the extremities. Our 3.0 Tesla systems have in place a means to monitor RF power levels and ensure that energy deposition is sufficiently low to stay well within these guidelines for temperature increases. First, a "system security" unit is employed to integrate the output of the RF amplifiers. This integration takes into account the amplitudes and duty cycle of the transmitter. If system security detects an output that might exceed the guidelines noted above, it automatically shuts down the entire RFpower system. Secondly, all pulse sequences are evaluated, based on calculations and sound scientific measurements, to ensure that SAR remains within FDA-approved guidelines, prior to their use in humans. Any experiment performed on our 3.0 Tesla system will comply with all FDA guidelines with regard to RF power deposition. Proper and routine monitoring of all RF electronics (e.g., coils, transmitters, system security, etc.) will be performed on a regular basis. All pulse sequences will be evaluated (by calculation and by valid scientific measurement) prior to use in humans.

### Venous Blood Sampling

Blood sampling in the amount of 30 mL or 2 tablespoons for safety laboratory tests and an additional 22 mL for RUCDR genetics, and ~40 mL for ketamine/norketamine/POMA levels is at a minimal level of risk, which includes slight pain, the possibility of bruising, and the possibility of feeling faint. Subjects will be advised of these risks.

### Intravenous Catheter

There is a small risk of infection and bleeding associated with intravenous catheters, which are prevented by proper techniques. Placement of IVs will be by a physician or nurse trained and certified in aseptic technique for catheter placement to minimize this risk.

### Interviews and Behavioral and Safety Assessments

Interviews and neuropsychological assessments are associated with minimal risk. Some participants may find the interviews and assessments tiring or distressing. Risks are to be minimized by allowing as much flexibility in the interview process (e.g., doing the interview in several meetings, giving breaks) as possible. If subjects have emotional responses, appropriate psychological support is given. Most patients find the interviews and assessments helpful.

### Pregnant or Nursing Females

Ketamine and POMA should not be used in pregnant females (ketamine only if absolutely medically necessary). For these reasons, pregnant females are excluded from the study. All female patients will demonstrate a negative serum human chorionic gonadotropin (HCG) test prior to study entry. Urine pregnancy tests will also be conducted on the scan days before administration of study drug to confirm that the patient has not become pregnant since the screening evaluation.

Any subject who becomes pregnant during the study will be discontinued immediately.

Females in the study who are capable of becoming pregnant must either be abstaining from sexual intercourse for the duration of the study or be using a medically acceptable form of contraception. Reliable methods of preventing pregnancy are hormonal contraceptives (the pill), double barrier methods (condom and spermicide), intra-uterine devices, and tubal ligation. Men must also agree to use a medically acceptable form of contraception.

Potential study participants who cannot agree to consistently practice effective birth control, or who the investigator judges to be unreliable in practicing birth control will not be included in the study.

If a study participant becomes pregnant while participating in the study, she will be discontinued from the study drug immediately. Pregnancies in female study participants and female partners of male study participants should be handled in the same manner. The investigator will follow the pregnancy until completion or until pregnancy termination.

Because the effects of MRI and ketamine on young children are also unknown, nursing females are also excluded from this study.

### Emergencies

If, during the course of study procedures, study staff identifies a condition that mandates immediate clinical intervention or official reporting (e.g., homicidality/suicidality), all necessary steps will be taken, and the emergency procedures at the respective institutions will be followed. In the case that staff determines that the participant is at significant risk for self-or-other-destructive behavior, one of the PIs will be contacted and necessary treatment steps will be taken (e.g., hospitalization, referral to a care provider). All subjects will be given numbers to call in the event of an emergency during treatment. If the site-PI, a covering MD in the subject’s clinic, or the Doctor-on-Call is immediately unavailable, subjects will be instructed to not delay and to go to their nearest Emergency Room.

### Safety Measures Upon Termination

Full laboratory evaluation (i.e., basic chemistries, liver function tests, complete blood count, thyroid stimulating hormone, urinalysis), EKG, and physical examination will be completed on Day 10. The SAFTEE (37) will be used to assess general side effects and will be performed after study procedures, as will vital signs assessment. If necessary, subjects will be asked to return one week after completion of the active phase of the study to follow up on any abnormalities in EKG, physical examination, or laboratories.

### Genetic blood draw

Subjects will have the option of participating in a genetic blood draw. While we will use many safety measures to protect subject privacy, we cannot guarantee a subject’s identity will never become known. While neither the public nor the controlled-access databases developed for this project will contain information that is traditionally used to identify a subject, such as their name, address, telephone number, or social security number, people may develop ways in the future that would allow someone to link a subjects genetic or medical information in our databases back to the subject. For example, someone could compare information in our databases with information from a subject (or a blood relative) in another database and be able to identify a subject (or your blood relative). It also is possible that there could be violations to the security of the computer systems used to store the codes linking the genetic and medical information to the subject.

Since some genetic variations can help to predict the future health problems of you and your relatives, this information might be of interest to health providers, life insurance companies, and others. Patterns of genetic variation also can be used by law enforcement agencies to identify a person or his/her blood relatives. Therefore, a subject’s genetic information potentially could be used in ways that could cause a subject or their family distress, such as by revealing that the subject (or a blood relative) carry a genetic disease.

There also may be other privacy risks that we have not foreseen.

There are state and federal laws that protect against genetic discrimination. There is a new federal law called the Genetic Information Nondiscrimination Act (GINA). In general, this law makes it illegal for health insurance companies, group health plans, and most employers to discriminate against anyone based on their genetic information. However, it does not protect someone against discrimination by companies that sell life insurance, disability insurance, or long-term care insurance.

### Recruitment and Informed Consent

Recruitment procedures have been described elsewhere. Subjects will be obtained through: (a) word-of-mouth, (b) publicity about the study, including articles in local newspapers and magazines, appearances on local radio and television shows, etc., leading to self-referral of prospective subjects, and (c) IRB-approved advertisements placed in local media/Internet.

Written informed consent, approved by the central and local IRB, will be obtained from each participant prior to entering the study. The informed consent document will explain in simple terms, before the subject is entered into the study, the risks and benefits to the subject. The informed consent document will contain a statement that the consent is freely given, that the subject is aware of the risks and benefits of entering the study, and that the subject is free to withdraw from the study at any time. The nature of the procedures and the alternatives to study participation will be discussed with each subject prior to obtaining written informed consent. Subjects will be informed that the information they provide will be kept confidential except within the research team and how that confidentiality will be assured. They will be told that their records are filed by number, not by name, and that all records are kept in locked files accessible only to research personnel. Consent will be obtained after a thorough explanation of the study by the **study physician** ~~site-PIs~~ and an opportunity for the participant to ask questions about the study. The consent form will be signed and dated by the subject and **study physician** ~~site-PIs~~.

It will be the responsibility of the site-PIs to ensure that an informed consent form is obtained from each participant and to obtain the appropriate signatures and dates on the informed consent document prior to the performance of any protocol procedures and in accordance with current state and federal regulations. The signed informed consent document will be retained with study records. Each participant will be given a copy of his or her signed informed consent.

Protection Against Risk: Subjects are safeguarded from undue risk by procedures to obtain informed consent, ensure confidentiality, and minimize possible risks associated with the study. Each is described below.

1. *Informed Consent*: In the consent form and in the consent discussion, subjects will be advised fully of the study procedures, the amount of time required of them, the possible risks and benefits, the voluntary nature of their participation, their right to refuse participation without prejudice, their right to terminate participation at any moment without prejudice, and the name and telephone number of the Principal Investigators.
2. *Confidentiality*: In the informed consent form, subjects will be told that the information they provide and all findings will be kept strictly confidential, with access limited to the research staff, with one exception: state or federal regulatory personnel and legal advocacy organizations authorized by law will have access to review records. Data collected with identifying information will be stored in locked cabinets or in password- protected computer files. Subject identity will not be revealed in the presentation or publication of any results. All staff working on the project will be educated about the importance of strictly respecting patient confidentiality.
3. *Research Procedures*: Described above are the potential risks of the research procedures and specific measures to minimize each of those risks. Below are general safeguards that will be used to minimize risks. These include exclusion of subjects at highest risk, the monitoring of any side effects, and the termination of subjects from research participation if it is believed that such participation endangers their welfare.
   1. Careful medical and psychiatric screening to identify subject whose risk for potential adverse effects would be elevated with study procedures. Such subjects will be excluded from the study. As an example, an actively suicidal person would be excluded from study participation and referred for appropriate treatment.
   2. Careful monitoring of subjects during the assessment and study period by experienced clinicians. Staff psychiatrists will be available 24 hours per day for clinical emergencies.
   3. Subjects who begin the study and experience adverse effects sufficient to require removal from the study will be referred for appropriate clinical care. The exact nature of “appropriate clinical care” will be determined by the judgment of clinicians familiar with the specific subject.
   4. As in any type of research, subjects' confidentiality must be carefully guarded and respected. All data with identifying information will be stored in locked file cabinets or password-protected computer files. Data being analyzed will be identified by subject codes and identifying information will be removed. The identity of subjects will not be revealed in the presentation or publication of any results. All individuals working on the project will be educated about the importance of strictly respecting subjects' rights to confidentiality.

# CLASSIFICATION OF RISK (FOR THE STUDY AS A WHOLE)

## Overall Risk and Benefit Consideration

The risks for all subjects are more than minimal but present a reasonable opportunity to further the understanding, prevention or alleviation of a serious problem affecting the health or welfare of individuals between the ages of 18-55.

The mild risks that this study poses to subjects are reasonable in relation to the anticipated benefits and potential heuristic value of the research. The results of this study are expected to help us determine the feasibility of continuing to develop medications that target the mGluR2/3 for schizophrenia, which will possibly lead to better treatments. Given the importance of the information to be gained, the risks to human subjects are reasonable.

## Children

We are excluding children under the age of 18 from the study proposed in this application, but will include children 18-21. In New York State, Alabama and California, subjects age 18 and over can consent on their own for research such as that described in this application. The number of subjects age 18-21 years to be enrolled in these studies is estimated based on our previous experience in recruitment and will be approximately 10% of the total sample to be studied (N≈8 healthy control subjects). Our research group has significant experience working with healthy control subjects between the ages of 18-21. Children younger than 18 will not be included because: 1) excluding those under 18 will reduce the influence of developmental differences in the processes that we aim to study, and 2) the potential benefits of these studies do not warrant exposing children under the age of 18 to the potential risks, in particular the risks related to ketamine and experimental agents.

# ALTERNATIVES TO PARTICIPATION OR ALTERNATIVE THERAPIES

Subjects do not receive any treatment in this study or need to forego any treatment in order to participate in this study. The alternative, therefore, is not to participate.

# CONSENT DOCUMENTS AND PROCESS

## Designation of Those Obtaining Consent

Study investigators designated as able to obtain consent in Section 9.2., will obtain informed consent. Assent will be obtained from minor subjects.

## Consent Procedures

Written informed consent, approved by the relevant IRBs and the NIMH DSMB, will be obtained from each participant prior to entering the study. The informed consent document will explain in simple terms (written at the 8^th^ grade reading level as assessed by the Flesch-Kincaid system), before the subject is entered into the study, the risks and benefits to the subject. The informed consent document will contain a statement that the consent is freely given, that the subject is aware of the risks and benefits of entering the study, and that the subject is free to withdraw from the study at any time.

The nature of the procedures and the alternatives to study participation will be discussed with each subject prior to obtaining written informed consent by the site study physician. Subjects will be informed that the information they provide will be kept confidential except within the research team and how that confidentiality will be assured. They will be told that their records are filed by number, not by name, and that all records are kept in locked files accessible only to research personnel. Consent will be obtained after a thorough explanation of the study by the site-**study physician or site-PI** and an opportunity for the participant to ask questions about the study. The consent form will be signed and dated by the subject and **study physician or site-PI**.

It will be the responsibility of the Site-PIs to ensure that an informed consent form is obtained from each participant and to obtain the appropriate signatures and dates on the informed consent document prior to the performance of any protocol procedures and in accordance with current state and federal regulations. The signed informed consent document will be retained with study records. Each participant will be given a copy of his or her signed informed consent.

Subjects will be informed that they will have the option to participate in genetic blood draws, and that they may decline this option while still participating in the main study.

## Protection Against Risk

In the consent form and in the consent discussion, subjects will be advised fully of the study procedures, the amount of time required of them, the possible risks and benefits, the voluntary nature of their participation, their right to refuse participation without prejudice, their right to terminate participation at any moment without prejudice, and the name and telephone number of the site-PIs.

# SUBJECT SAFETY MONITORING

Full laboratory evaluation (i.e., basic chemistries, liver function tests, complete blood count, thyroid stimulating hormone, urinalysis) and physical examination will be completed at baseline and on Day 10. All subjects will receive constant EKG monitoring during the study procedures on Day -10 and 10. The SAFTEE (60) will be used to assess general side effects and will be performed after study procedures on Days -10, 1, 5, and 10, as will vital signs assessment. If necessary, subjects will be asked to return one week after completion of the active phase of the study to follow up on any abnormalities in EKG, physical examination, or laboratories. Vital signs, including orthostatic blood pressure monitoring, will also occur on the study procedure days. Site-specific ranges for lab values will be utilized to determine inclusion/exclusion, accept for those values specifically referenced in the inclusion/exclusion criteria. All laboratory values will be reviewed by a study physician.

## Criteria for Stopping the Study or Suspending Enrollment or Procedures

For individual subjects, the stopping criteria are:

- Withdrawal of consent and/or patient decision;
- A seizure
- Pregnancy or loss to follow up;
- Any evidence of suicidality/homicidality (as assessed by the by a clinical interview by the study psychiatrist on every visit);
- Treatment with a new medication other than the use of appropriate medications for the treatment of AEs under direction of the investigator;
- Subject noncompliance, defined as refusal or inability to adhere to the trial schedule or procedures. Although Medication compliance will be monitored and recorded for use in statistical analysis (e.g. “per protocol” analysis), medication non-compliance alone will not lead to study discontinuation;
- Systolic BP increases to > 180 mm Hg and remains > 180 mm Hg for more than 2 minutes, or diastolic BP increases to > 110 mm Hg and remains > 110 mm Hg for more than 2 minutes;
- Clinically significant nausea or vomiting within 48 hours of a scheduled MRI will prompt an evaluation to terminate the subject.
- Adverse reaction to ketamine including emergence of psychosis or vomiting during the MRI;
- Clinically significant adverse events, which would be inconsistent with continuation in the study;
- Clinical judgment of the investigator or at request of subject, sponsor, or regulatory authority.

If necessary, subjects may be unblinded by contacting the local site pharmacy, who will keep a sealed envelope on blind assignment for each subject for use in an emergency.

For the whole study, stopping criteria are:

- a pattern of adverse events that are unexpected and potentially dangerous and potentially related to the study procedures;
- a pattern of adverse events that are expected and serious and occurring at a rate higher than expected and are potentially dangerous to subjects.
- Any seizure-like event will be presented to the study medical monitor within 24 hours for adjudication. If the event is determined not to be a seizure, the study will continue. If the nature of the event is unclear or if it is determined to be a seizure, the study will be stopped. A second seizure-like event will lead to immediate discontinuation of study medication (POMA and/or ketamine) dosing. All seizures or suspected seizures will be documented as an SAE.

If whole study stopping criteria are met, the study PI, site-PIs, and NIMH program staff will perform an investigation into the events that led to stopping of the trial. The trial may resume if an acceptable corrective action plan is possible and approved by the coordinating PI, site PIs, NIMH program staff, and NIMH DSMB.

# ADVERSE EVENT AND UNANTICIPATED PROBLEM REPORTING

An Adverse Event (AE) is any unwanted experience or event occurring in the course of a study or a clinical trial. An AE is defined as unexpected whenever its nature and severity is not consistent with the known product information. For abnormal lab values, the clinical significance will be determined by the study physician with the site principal investigator having ultimate authority on adverse event designations.

All AEs, serious and non-serious, expected and unexpected, related and unrelated to the study, will be documented and reported by the site principal investigator and site study physicians.

Information to be collected on AEs includes but is not limited to date of onset, assessment of severity, and relationship to study treatment, date of resolution of event, seriousness, any required treatment or evaluations, and outcome. The Site PI will be in charge of reporting all Serious Adverse Events (SAEs), in writing, to the Coordinating center (CC) who reports to the NIMH Data and Safety Monitoring Board (DSMB). All AEs, whether serious or non-serious, will be followed to resolution or until the AE is determined by the site-PI not to be clinically significant.

In addition, unanticipated problems that are not AEs will also be reported to the Central IRB (Columbia, UCLA, UAB) and to the local IRB (NYU) (if applicable) by the site principal investigator and site study physicians. Unanticipated problems are any event that is unexpected, related or possibly related to the research, and places either the subject or others at greater risk of harm. Throughout the study, notification of all SAEs, as well as any investigator-initiated changes in the protocol will be submitted to the NIMH Contractor Officer’s Representative (COR), NIMH CTOBB staff, NIMH DSMB, central IRB (Columbia, UCLA, UAB) and local IRB (NYU).

A SAE is defined as follows: death, life-threatening adverse event, inpatient hospitalization, persistent or significant disability/incapacity, congenital anomaly, and medically significant event (an event that requires medical or surgical intervention to prevent death, life- threatening adverse event, inpatient hospitalization, persistent or significant disability/incapacity or a congenital anomaly). Actions taken by one entity in response to adverse event reports will be reported to the other entities.

SAEs that are unexpected and likely related to the intervention will be reported to the NIMH DSMB on an expedited basis in writing within 10 business days. Serious, unexpected (defined in the Investigator Brochure), and related (defined by either investigator or sponsor) events will reported to the sponsor within 24 hrs and to the FDA within 7 calendar days for death or life-threatening events and 15 calendar days for non-fatal events. All other SAEs, expected or non-serious adverse events, will be reported to the DSMB, and relevant IRB’s in the quarterly DSMB data reports or at the time of continuing IRB review of the study.

# DATA AND SAFETY MONITORING

This study will be monitored by the study-PIs, study coordinators, NIMH Data and Safety Monitoring Board (DSMB), coordinating center, and data management center.

## NIMH DSMB Reporting

The main responsibilities of the NIMH DSMB include, but are not limited to the following: Review of protocols, consent procedures, consent forms, and safety plans prior to initiation of the study; Monitoring of the progress of the study, including recruitment and retention of participants, adverse events, serious adverse events (SAEs), reasons for participant withdrawal, adherence to the time line of the study, quality of data, and protocol violations; Making directives about the continuation, modification, or termination of the study, based on the balance of adverse events and beneficial outcomes. Throughout the study, notification of any Serious Adverse Events (SAEs) as well as any proposed investigator-initiated changes in the protocol will be submitted to the NIMH DSMB. Based on its review of the protocol, the NIMH DSMB will identify the data parameters and format of the information to be regularly reported. The NIMH DSMB may at any time request additional information from the Principal Investigators.

All SAEs and adverse events (AEs will only be reported to the NIMH DSMB annually) will be tabulated and submitted to the central and local IRBs and NIMH DSMB in the quarterly DSMB data reports or at the time of continuing review, IRB review of the study, although the NIMH DSMB can determine that more frequent meetings are indicated. Based on review of safety data, the NIMH DSMB will issue directives concerning the conduct of the study. Recommendation/directives made by the DSMB may include amending safety monitoring procedures, modifying the protocol or consent, terminating the study, or continuing the study as designed.

# QUALITY ASSURANCE

All studies will be reviewed on a regular basis by the Quality Assurance Staff of respective institutions, and will be overseen by the Data Management Center as described below.

## Data Management and Procedures

### Data Management

This protocol will utilize the Data Management Center (DMC) of NKI/RFMH. This centralized data management center will be responsible for development of the case report forms (CRFs), development and validation of the clinical study database, ensuring data integrity, and training clinical site and other protocol staff on applicable data management procedures and in the Acquire Electronic Data Capture (EDC) system, which is a web-based distributed data entry system. The Acquire EDC system meets all applicable guidelines and regulations surrounding the use of computerized systems in clinical trials.

### Data Collection Forms

Data will be collected at the study sites on paper CRFs. The DMC will provide sites with a final set of standardized forms, which are to be completed on an ongoing basis during the study. Forms should be completed within 48 hours after data collection discussed during training. The investigator is responsible for maintaining accurate, complete and up-to-date records and for tracking the completion of CRFs for each research participant. The investigator is also responsible for maintaining any source documentation related to the study, including any films, tracings, computer discs, or tapes.

### Data Acquisition and Entry

All paper CRFs must be completed legibly with black ballpoint pen. A correction should be made by striking through the incorrect entry with a single line and entering the correct information adjacent to the incorrect entry. Corrections to paper CRFs must be initialed and dated by the person making the correction. Data entered into electronic CRFs shall only be performed by authorized individuals. Corrections to electronic CRFs shall be tracked electronically with time, date, individual making the change, and what was changed. Selected CRFs also may require the investigators written signature or electronic signature, as appropriate. CRFs will be monitored for completeness, accuracy, legibility and attention to detail during the study. The investigator must retain a copy of all CRFs.

### Data Center Responsibilities

The DMC will 1) develop a data management plan and will conduct data management activities, 2) provide final CRFs for the collection of all data required by the study, 3) develop data dictionaries for each CRF that will comprehensively define each data element, 4) conduct ongoing data monitoring activities on study data from all participating clinical sites, 5) monitor any preliminary analysis data clean-up activities, 6) monitor final study data cleanup activities, 7) lock the study database, and 8) provide the final, locked study database to designated organizations and personnel.

### Data Editing

Completed forms/electronic data will be entered into the Acquire EDC system database. On-line, real time data editing will be conducted. If incomplete or inaccurate data are found, a data clarification or Query will be sent to the site through the Acquire EDC system. Sites are expected to resolve queries within 7 days as will be noted during training.

### Documentation

Study documentation includes all case report forms, data correction forms, electronic data files, workbooks, source documents, monitoring logs, appointment schedules, sponsor-investigator correspondence and regulatory documents (e.g., signed protocol and amendments, IRB correspondence and approved consent form and signed patient consent forms, Statement of Investigator form, clinical supplies receipt and distribution records).

Source documents include all recordings of observations or notations of clinical activities and all reports and records necessary for the evaluation and reconstruction of the clinical research study. Source documents include, but are not limited to: laboratory reports, ECG tracings, X-rays, radiologist reports, patient diaries, ultrasound photographs, patient progress notes, hospital charts, pharmacy records, and any other similar reports or records of any procedure performed in accordance with the protocol.

Whenever possible, the original recording of an observation should be retained as the source document; however, a photocopy is acceptable provided that it is a clear, legible, and exact duplication of the original document.

### Training

The DMC will provide comprehensive training on the Acquire EDC system and related issues, such as transfer of data from paper CRFs to the EDC. Training is Web-based and mandatory for any staff entering data into the Acquire EDC system. Used IDs will be distributed following training and may not be shared with anyone else.

### Data Lock

After review by the data manager and the resolution of any validation issue, a study form may be locked. Forms are locked throughout the on-going study. Sites are locked after last subject last data is entered, reviewed by the data manager and all validation issues resolved. When all sites are locked, the study is locked per DMC Standard Operating Procedures (SOPs) and data is sent to study statistician. When sites are locked, permissions are moved to “read-only” status and remain available to view for six months following study closure. Studies are archived for a minimum of 10 years following study closure per DMC SOPs.

## Data Sharing

### NDCT Data Sharing

NIH requires that data from this study are made available to the research community through its National Database for Clinical Trials (NDCT). To support the submission of the study data to the NDCT, the DMC will assist the study sites in creating Global Unique Identifiers (GUIDs) for all subjects. A form with the required personal information will be completed shortly after the subject is registered and will be used by the DMC to generate a GUID. If the required information is not available, a pseudo-GUID will be generated. All data submitted to the NDCT data repository must have a GUID or pseudo-GUID identifier. Once the GUID is generated, the database form with the required personal information will no longer be viewable nor will it be exported.

The DMC will do the following prior to the expected submission date:

1. Submit a signed Data Sharing Agreement to NDCT
2. Generate a list of all data expected for this study
3. Generate and send data dictionaries to NDCT
4. Respond to any changes requested by the NDCT
5. Prepare data for submission
6. Submit raw and descriptive data on schedule with biannual NDCT’s submission schedule
7. Submit analyzed data upon completion of the study
8. Notify NDCT when related publications have been accepted
9. Respond to any post-submission queries

## Clinical Study Monitoring

The RFMH/NKI DMC will provide clinical trial monitoring services to ensure site compliance with International Committee on Harmonization (ICH), Good Clinical Practice (GCP) guidelines, Food and Drug Administration (FDA) regulations, human subject protection and safety, and protocol adherence. The Clinical Research Associate (CRA) will visit each site for a Site Initiation Visit, 3 interim visits and a closeout visit. All scheduling will be done with the site-PI through the Study Project Manager and each visit will consist of a review of all signed Informed Consents, a source documentation verification (SDV) of a select number of randomized subjects, and a review of the Regulatory Binder. The monitor will meet with the Site-PI at each visit and review findings. All findings will be communicated with the site-PI and the site coordinator in a follow-up letter. An Interim Monitoring Report and a Closeout Monitoring Report will be submitted to the Contract PI, Study Project Manager, and NIMH staff.

# CONFIDENTIALITY

## Research Data and Investigator Medical Records

Blood and urine samples, behavioral assessments, MR acquisitions, EEG data, and all other clinical/neuropsychological data will be obtained from the subjects for specific research purposes. Data will include self-report information, observer records, and physiological and behavioral information collected during test sessions. To insure database security, each subject is assigned a unique ID and all data related to that subject is entered at the site by the site data entry personnel. The database files are stored on an encrypted, dedicated SQL Server, which has no access to the Internet. This database server is fully secured, requiring a secondary username and password to access any of the data located within the database server. NKI employs two Cisco 5520x Firewalls in an Active/Active scenario to protect the internal network and all servers from unauthorized access. Each Cisco firewall utilized AES-256 encryption algorithms to further protect the internal servers.

Hard copies of all information will be kept locked in confidential files at each site. Electronic transmission of information regarding a subject will only use the assigned identifier for that subject.

Data from medical records may also be used on occasion with appropriate permission signed release from patient. The information gathered, excluding identifying information, will be shared with co- investigators and staff involved in this study; otherwise the information remains confidential and will be used only for research purposes and in accordance with IRB regulations. The data manager will perform random data audits to further ensure the integrity of the data. Server backups of all study databases are performed nightly and encrypted using our backup software to LTO-5 tapes. Every morning, these tape backups are removed from the Computing Center and relocated to a separate building (in a secured room) located on the NKI campus. For archival purposes, database copies are encrypted and stored on DVDs and stored separately in a fireproof, secured cabinet. NKI also employs an off-site, third party backup provider to store our encrypted tape backups in their climate-controlled, secured vault for seven years.

De-identified results from clinical trials will be posted on ClinicalTrials.gov by the sponsor.

## Further Protection against Risk

In the informed consent form, subjects will be told that the information they provide and all findings will be kept strictly confidential, with access limited to the research staff, with one exception: state or federal regulatory personnel and legal advocacy organizations authorized by law will have access to review records. Data collected with identifying information will be stored in locked cabinets or in password-protected computer files. Subject identity will not be revealed in the presentation or publication of any results. All staff working on the project will be educated about the importance of strictly respecting patient confidentiality.

# CONFLICT OF INTEREST

NIH guidelines on conflict of interest have been distributed to all investigators. All investigators will abide by the conflict-of-interest policies of their own institutions. DeNovo and Lilly will donate POMA for use in this study. No personal identifiers of participants will be shared with the DeNovo and Lilly. In addition, they will have no other role in the design, conduct, or interpretation of this trial.

# TECHNOLOGY TRANSFER

We do not anticipate any intellectual properties generated from this project.

# RESEARCH AND TRAVEL COMPENSATION

Volunteers will be compensated for time and research-related inconveniences:

- $50 for the screening visit 1 and visit 3 and 4
- $100 for screening ketamine MRI (Visit 2)
- $300 for completion of MRI Day 10 (5)
- If they are admitted for an overnight stay after a ketamine infusion, they will be compensated an additional $50.
- $50 every time you come as a standby subject
- $50 dollars for the genetics blood draw

Compensation will be pro-rated for parts completed if subjects do not complete the study. Subjects will be paid in either check or cash. If they receive a check, the check may be sent as late as after their participation in the study is complete. We will compensate for reasonable local travel expenses.

# REFERENCES

1. Gozzi A, Herdon H, Schwarz A, Bertani S, Crestan V, Turrini G, et al. Pharmacological stimulation of NMDA receptors via co-agonist site suppresses fMRI response to phencyclidine in the rat. Psychopharmacology. 2008;201(2):273-84.

2. Moghaddam B, Adams BW. Reversal of phencyclidine effects by a group II metabotropic glutamate receptor agonist in rats. Science (New York, NY). 1998;281(5381):1349-52.

3. Kahn RS, Fleischhacker WW, Boter H, Davidson M, Vergouwe Y, Keet IP, et al. Effectiveness of antipsychotic drugs in first-episode schizophrenia and schizophreniform disorder: an open randomised clinical trial. Lancet. 2008;371(9618):1085-97.

4. Lieberman JA, Stroup TS, McEvoy JP, Swartz MS, Rosenheck RA, Perkins DO, et al. Effectiveness of Antipsychotic Drugs in Patients with Chronic Schizophrenia. New England Journal of Medicine. 2005;353(12):1209-53.

5. Javitt DC, Zukin SR. Recent advances in the phencyclidine model of schizophrenia. The American journal of psychiatry. 1991;148(10):1301-8.

6. Krystal JH, Karper LP, Seibyl JP, Freeman GK, Delaney R, Bremner JD, et al. Subanesthetic effects of the noncompetitive NMDA antagonist, ketamine, in humans. Psychotomimetic, perceptual, cognitive, and neuroendocrine responses. Archives of general psychiatry. 1994;51(3):199-214.

7. Moghaddam B, Adams B, Verma A, Daly D. Activation of glutamatergic neurotransmission by ketamine: a novel step in the pathway from NMDA receptor blockade to dopaminergic and cognitive disruptions associated with the prefrontal cortex. The Journal of neuroscience : the official journal of the Society for Neuroscience. 1997;17(8):2921-7.

8. Moghaddam B, Krystal JH. Capturing the angel in "angel dust": twenty years of translational neuroscience studies of NMDA receptor antagonists in animals and humans. Schizophrenia bulletin. 2012;38(5):942-9.

9. Stauffer VL, Millen BA, Andersen S, Kinon BJ, Lagrandeur L, Lindenmayer JP, et al. Pomaglumetad methionil: no significant difference as an adjunctive treatment for patients with prominent negative symptoms of schizophrenia compared to placebo. Schizophrenia research. 2013;150(2-3):434-41.

10. Arango C, Nasrallah AT, Lawrie S, Lohmann TO, Zhu JL, Garibaldi G, et al. Efﬁcacy and safety of adjunctive bitopertin (5 and 10 mg) versus placebo in subjects with persistent predominant negative symptoms of schizophrenia treated with antipsychotics — Results from the phase III DayLyte study. Schiz Res. 2014;158(e1).

11. Blaettler T, Bugarski-Kirola D, Fleischhacker WW, Bressan R, Arango C, Abi-Sabb D, et al. Efﬁcacy and safety of adjunctive bitopertin (10 and 20 mg) versus placebo in subjects with persistent predominant negative symptoms of schizophrenia treated with antipsychotics — Results from the Phase III FlashLyte Study. Schiz Res. 2014;158(e2-e3).

12. Kinon BJ, Adams DH, Baygani S, Millen B, Velona I, Kollack-Walker S. A long-term, phase 2, safety study of LY2140023 monohydrate vs. Atypical antipsychotic standard of care in schizophrenia. Schizophrenia bulletin. 2011;37(Suppl 1):311.

13. Kinon BJ, Gomez JC. Clinical development of pomaglumetad methionil: a non-dopaminergic treatment for schizophrenia. Neuropharmacology. 2013;66:82-6.

14. Gozzi A, Large CH, Schwarz A, Bertani S, Crestan V, Bifone A. Differential effects of antipsychotic and glutamatergic agents on the phMRI response to phencyclidine. Neuropsychopharmacology : official publication of the American College of Neuropsychopharmacology. 2008;33(7):1690-703.

15. Zeiler FA, Sader N, Gillman LM, Teitelbaum J, West M, Kazina CJ. The Cerebrovascular Response to Ketamine: A Systematic Review of the Animal and Human Literature. J Neurosurg Anesthesiol. 2015.

16. Bartha R, Williamson PC, Drost DJ, Malla A, Carr TJ, Cortese L, et al. Measurement of glutamate and glutamine in the medial prefrontal cortex of never-treated schizophrenic patients and healthy controls by proton magnetic resonance spectroscopy. Archives of general psychiatry. 1997;54(10):959-65.

17. Theberge J, Bartha R, Drost DJ, Menon RS, Malla A, Takhar J, et al. Glutamate and glutamine measured with 4.0 T proton MRS in never-treated patients with schizophrenia and healthy volunteers. The American journal of psychiatry. 2002;159(11):1944-6.

18. de la Fuente-Sandoval C, Leon-Ortiz P, Favila R, Stephano S, Mamo D, Ramirez-Bermudez J, et al. Higher levels of glutamate in the associative-striatum of subjects with prodromal symptoms of schizophrenia and patients with first-episode psychosis. Neuropsychopharmacology : official publication of the American College of Neuropsychopharmacology. 2011;36(9):1781-91.

19. Theberge J, Williamson KE, Aoyama N, Drost DJ, Manchanda R, Malla AK, et al. Longitudinal grey-matter and glutamatergic losses in first-episode schizophrenia. The British journal of psychiatry : the journal of mental science. 2007;191:325-34.

20. Kegeles LS, Mao X, Stanford AD, Girgis R, Ojeil N, Xu X, et al. Elevated prefrontal cortex gamma-aminobutyric acid and glutamate-glutamine levels in schizophrenia measured in vivo with proton magnetic resonance spectroscopy. Archives of general psychiatry. 2012;69(5):449-59.

21. Kantrowitz JT, Javitt DC. Glutamatergic Approaches to the Conceptualization and Treatment of Schizophrenia. In: Javitt DC, Kantrowitz JT, editors. Handbook of Neurochemistry and Molecular Neurobiology 3rd Edition. New York City: Springer; 2009.

22. Baker DA, Madayag A, Kristiansen LV, Meador-Woodruff JH, Haroutunian V, Raju I. Contribution of cystine-glutamate antiporters to the psychotomimetic effects of phencyclidine. Neuropsychopharmacology : official publication of the American College of Neuropsychopharmacology. 2008;33(7):1760-72.

23. Lorrain DS, Baccei CS, Bristow LJ, Anderson JJ, Varney MA. Effects of ketamine and N-methyl-D-aspartate on glutamate and dopamine release in the rat prefrontal cortex: modulation by a group II selective metabotropic glutamate receptor agonist LY379268. Neuroscience. 2003;117(3):697-706.

24. Krystal JH, Abi-Saab W, Perry E, D'Souza DC, Liu N, Gueorguieva R, et al. Preliminary evidence of attenuation of the disruptive effects of the NMDA glutamate receptor antagonist, ketamine, on working memory by pretreatment with the group II metabotropic glutamate receptor agonist, LY354740, in healthy human subjects. Psychopharmacology. 2005;179(1):303-9.

25. Herman EJ, Bubser M, Conn PJ, Jones CK. Metabotropic glutamate receptors for new treatments in schizophrenia. Handb Exp Pharmacol. 2012(213):297-365.

26. Patil ST, Zhang L, Martenyi F, Lowe SL, Jackson KA, Andreev BV, et al. Activation of mGlu2/3 receptors as a new approach to treat schizophrenia: a randomized Phase 2 clinical trial. Nat Med. 2007;13(9):1102-7.

27. Kinon BJ, Zhang L, Millen BA, Osuntokun OO, Williams JE, Kollack-Walker S, et al. A multicenter, inpatient, phase 2, double-blind, placebo-controlled dose-ranging study of LY2140023 monohydrate in patients with DSM-IV schizophrenia. Journal of clinical psychopharmacology. 2011;31(3):349-55.

28. Deakin JF, Lees J, McKie S, Hallak JE, Williams SR, Dursun SM. Glutamate and the neural basis of the subjective effects of ketamine: a pharmaco-magnetic resonance imaging study. Archives of general psychiatry. 2008;65(2):154-64.

29. Driesen NR, McCarthy G, Bhagwagar Z, Bloch M, Calhoun V, D'Souza DC, et al. Relationship of resting brain hyperconnectivity and schizophrenia-like symptoms produced by the NMDA receptor antagonist ketamine in humans. Molecular psychiatry. 2013;18(11):1199-204.

30. D'Souza DC, Ahn K, Bhakta S, Elander J, Singh N, Nadim H, et al. Nicotine fails to attenuate ketamine-induced cognitive deficits and negative and positive symptoms in humans: implications for schizophrenia. Biological psychiatry. 2012;72(9):785-94.

31. Kegeles LS, Martinez D, Kochan LD, Hwang DR, Huang Y, Mawlawi O, et al. NMDA antagonist effects on striatal dopamine release: positron emission tomography studies in humans. Synapse (New York, NY). 2002;43(1):19-29.

32. Perry EB, Jr., Cramer JA, Cho HS, Petrakis IL, Karper LP, Genovese A, et al. Psychiatric safety of ketamine in psychopharmacology research. Psychopharmacology. 2007;192(2):253-60.

33. Krystal JH, Karper LP, Bennett A, D'Souza DC, Abi-Dargham A, Morrissey K, et al. Interactive effects of subanesthetic ketamine and subhypnotic lorazepam in humans. Psychopharmacology. 1998;135(3):213-29.

34. Krystal JH, D'Souza DC, Karper LP, Bennett A, Abi-Dargham A, Abi-Saab D, et al. Interactive effects of subanesthetic ketamine and haloperidol in healthy humans. Psychopharmacology. 1999;145(2):193-204.

35. Anand A, Charney DS, Oren DA, Berman RM, Hu XS, Cappiello A, et al. Attenuation of the neuropsychiatric effects of ketamine with lamotrigine: support for hyperglutamatergic effects of N-methyl-D- aspartate receptor antagonists. Archives of general psychiatry. 2000;57(3):270-6.

36. Krystal JH, Madonick S, Perry E, Gueorguieva R, Brush L, Wray Y, et al. Potentiation of low dose ketamine effects by naltrexone: potential implications for the pharmacotherapy of alcoholism. Neuropsychopharmacology : official publication of the American College of Neuropsychopharmacology. 2006;31(8):1793-800.

37. Levine J, Schooler NR. SAFTEE: a technique for the systematic assessment of side effects in clinical trials. Psychopharmacol Bull. 1986;22(2):343-81.

38. Overall JE, Gorham DE. The Brief Psychiatric Rating Scale. Psychol Reports. 1961;10:799-812.

39. Shah YB, Marsden CA. The application of functional magnetic resonance imaging to neuropharmacology. Current opinion in pharmacology. 2004;4(5):517-21.

40. Steward CA, Marsden CA, Prior MJ, Morris PG, Shah YB. Methodological considerations in rat brain BOLD contrast pharmacological MRI. Psychopharmacology. 2005;180(4):687-704.

41. De Simoni S, Schwarz AJ, O'Daly OG, Marquand AF, Brittain C, Gonzales C, et al. Test-retest reliability of the BOLD pharmacological MRI response to ketamine in healthy volunteers. NeuroImage. 2013;64:75-90.

42. Kegeles LS, Abi-Dargham A, Zea-Ponce Y, Rodenhiser-Hill J, Mann JJ, Van Heertum RL, et al. Modulation of amphetamine-induced striatal dopamine release by ketamine in humans: implications for schizophrenia. Biological psychiatry. 2000;48(7):627-40.

43. Diamond PR, Farmery AD, Atkinson S, Haldar J, Williams N, Cowen PJ, et al. Ketamine infusions for treatment resistant depression: a series of 28 patients treated weekly or twice weekly in an ECT clinic. Journal of psychopharmacology (Oxford, England). 2014;28(6):536-44.

44. Murrough JW, Perez AM, Pillemer S, Stern J, Parides MK, aan het Rot M, et al. Rapid and longer-term antidepressant effects of repeated ketamine infusions in treatment-resistant major depression. Biological psychiatry. 2013;74(4):250-6.
