# Supplementary material for: Proof of mechanism and target engagement of glutamatergic drugs for the treatment of schizophrenia: RCTs of pomaglumetad and TS-134 on ketamine-induced psychotic symptoms and pharmacoBOLD in healthy volunteers
Source: Neuropsychopharmacology. 2020 May 13;45(11):1842–50. doi: 10.1038/s41386-020-0706-z (PMC7608251; doi:10.1038/s41386-020-0706-z)
Supplement: Supplementary file 10 — Taisho Protocol [file 41386_2020_706_MOESM10_ESM.pdf]

Protocol Number: TS134-US103

A Randomized, Single-blind, Parallel-group Study to Evaluate the Effects of TS-134 on Ketamine-induced BOLD Signals in Resting fMRI in Healthy Adult Subjects

Investigational Product: TS-134

Sponsor: Taisho Pharmaceutical R&D Inc.
350 Mt. Kemble Avenue
Morristown, NJ 07960
Tel: 1-973-285-0870
Fax: 1-973-285-1665

Version: Amendment 1

Document Dates: 19-JULY-2017

Original Protocol: Final, 13-MARCH-2017

Confidentiality Statement

The information in this document is confidential and is not to be disclosed without the written consent of Taisho Pharmaceutical R&D Inc. except to the extent that disclosure would be required by law and for the purpose of evaluating and/or conducting a clinical study for Taisho Pharmaceutical R&D Inc. You are allowed to disclose the contents of this document only to your Institutional Review Board (IRB) and study personnel directly involved with conducting this protocol. Persons to whom the information is disclosed must be informed that the information is confidential and proprietary to Taisho Pharmaceutical R&D Inc. and that it may not be further disclosed to third parties.

PROTOCOL SIGNATURE SHEET

A Randomized, Single-blind, Parallel-group Study
to Evaluate the Effects of TS-134 on Ketamine-induced BOLD Signal
in Resting fMRI in Healthy Adult Subjects

Protocol Number: TS134-US103

Protocol Version: Amendment 1

By my signature below, I approve this protocol.

Approval Signatures:

Sponsor Company: ______________________________ ________________

[redacted] Date

Taisho Pharmaceutical R&D Inc.
350 Mt. Kemble Avenue
Morristown, NJ 07960
Phone [redacted] Fax: [redacted]
Email [redacted]

Medical Monitor: ______________________________ ________________

[redacted] Date

Consulting Medical Monitor
[redacted]
Phone: [redacted]
Email [redacted]

INVESTIGATOR SIGNATURE SHEET

A Randomized, Single-blind, Parallel-group Study
to Evaluate the Effects of TS-134 on Ketamine-induced BOLD Signal
in Resting fMRI in Healthy Adult Subjects

Protocol Number: TS134-US103

Protocol Version: Amendment 1

By signing below, I attest that I have read, understood, and agree to abide by all conditions, instructions, and restrictions contained in this protocol. I will not initiate this study without approval from the appropriate Institutional Review Board (IRB) and I understand that any changes in the protocol must be approved in writing by the Sponsor and the IRB before they can be implemented, except where necessary to eliminate immediate hazards to the subject.

Approval Signature:

Principal Investigator: ______________________________ ____________

Jeffrey A. Lieberman, M.D. Date

____

Name of Facility

____

Address

____

City, State, Zip Code

____

Phone Number

____

Fax Number

STUDY CONTACT PAGE

Sponsor Contact:	[redacted]<br>Taisho Pharmaceutical R&D Inc.<br>350 Mount Kemble Avenue<br>Mount Kemble Corporate Center<br>Morristown, NJ 07960<br>Phone: [redacted]<br>Fax: [redacted]<br>Email: [redacted]
Research Coordinator Contact:	Marlene M. Carlson, MPH<br>Research Coordinator<br>New York State Psychiatric Institute<br>1051 Riverside Drive, Unit 4 Room 6708<br>New York, NY 10032<br>Phone: 646-774-8436<br>Fax: 646-774-5316<br>Email: mc157@columbia.edu

1 TABLE OF CONTENTS

PROTOCOL SIGNATURE SHEET 2
INVESTIGATOR SIGNATURE SHEET 3
STUDY CONTACT PAGE 4
1 TABLE OF CONTENTS 5
2 LIST OF ABBREVIATIONS 10
3 SYNOPSIS 12
4 BACKGROUND INFORMATION 14
4.1 Name and Description of Investigational Product 15
4.2 Summary of Non-Clinical and Clinical Findings 16
4.2.1 Summary of Non-Clinical Findings 16
4.2.2 Summary of Clinical Findings 16
4.3 Study Rationale 16
5 STUDY OBJECTIVES 18
5.1 Primary Objective 18
5.2 Secondary Objectives 18
6 STUDY DESIGN AND EVALUATION 19
6.1 Study Design 19
6.2 Study Endpoints 20
6.2.1 Primary Endpoint 20
6.2.2 Secondary Endpoints 20
7 STUDY POPULATION 21
7.1 Inclusion Criteria 21
7.2 Exclusion Criteria 21
8 TREATMENT OF SUBJECTS 24
8.1 Investigational Product 24
8.1.1 Packaging and Labeling 24

8.1.2 Handling and Dispensing of Investigational Product 24
8.1.3 Randomization Procedure 25
8.1.4 Blinding 25
8.1.5 Treatment Compliance 25
8.1.6 Investigational Product Accountability 26
8.1.7 Return and Destruction of Investigational Product 26
8.2 Concomitant Drug Therapy 26
8.3 Duration of Subject Participation 27
8.4 Discontinuation Criteria and Procedures 27
9 STUDY PROCEDURES AND OBSERVATIONS 28
9.1 Events Schedule 28
9.2 Procedures by Visit 30
9.2.1 Screening Period (Day -28 to Day -2) 30
9.2.1.1 Initial Screening Visit(s) 30
9.2.1.2 Final Screening Visit (First Ketamine Session) 31
9.2.2 Treatment Period (Day -1 to Day 7) 32
9.2.2.1 Day -1 (Admission) 32
9.2.2.2 Day 1 to Day 5 33
9.2.2.3 Day 6 (End of Treatment: Second Ketamine Session) 33
9.2.2.4 Day 7 (Discharge) / Early Termination (ET) 34
9.2.3 Phone Call Follow-Up (Day 14) 35
9.2.4 Early Termination (ET) Phone Call Follow-Up 35
9.3 Study Procedures 35
9.3.1 Study Materials 35
9.3.2 Subject Background Assessments 35
9.3.2.1 Demographics & Medical/ Psychiatric History 35
9.3.2.2 Structured Clinical Interview for DSM-5 Disorders – Clinical Trials Version 35
(SCID-5-CT) 35

9.3.2.3 Height 36
9.3.2.4 Body Weight 36
9.3.2.5 Body Mass Index (BMI) 36
9.3.3 Safety Assessments 36
9.3.3.1 Physical Examination (including a neurological examination) 36
9.3.3.2 Vital Signs 37
9.3.3.3 Electrocardiogram 37
9.3.3.4 Columbia - Suicide Severity Rating Scale (C-SSRS) 38
9.3.4 Clinical Laboratory Tests 38
9.3.4.1 Serology Screen 39
9.3.4.2 Urine Cotinine Screen 39
9.3.4.3 Saliva Alcohol Screen 39
9.3.4.4 Urine Drug Screen 39
9.3.4.5 Serum/Urine Pregnancy 40
9.3.4.6 Hematology and Biochemistry 40
9.3.4.7 Urinalysis 40
9.3.5 Pharmacodynamics (PD) Assessments 40
9.3.5.1 MRI Assessments with Ketamine Infusion 40
9.3.5.2 Behavioral Assessments 42
9.3.6 Assessments of Pharmacokinetics (PK) 43
9.3.6.1 MGS0008 43
9.3.6.2 Ketamine 44
9.3.7 Pharmacogenomic (PGx) Blood Sampling 44
9.3.8 Blood Sample Volume for the Study 45
9.4 Time Tolerances and Windows 45
10 STATISTICAL METHODS 46
10.1 Sample Size Determination 46
10.2 Analysis Populations 46

10.3 General Analysis Conventions 46
10.4 Demographic and Baseline Characteristics 46
10.5 Pharmacodynamics (PD) Analysis 47
10.5.1 Primary Endpoint 47
10.5.2 Secondary Endpoint 47
10.5.3 Interim Data Transfer 47
10.6 Safety Analysis 47
10.6.1 Adverse Events 47
10.6.2 Laboratory Values 48
10.6.3 Vital Signs 48
10.6.4 Electrocardiogram 48
10.6.5 C-SSRS 48
10.7 Pharmacokinetics (PK) 49
10.7.1 MGS0008 49
10.7.2 Ketamine 49
10.8 Pharmacogenomic (PGx) Analysis 49
11 ADVERSE EVENT REPORTING 50
11.1 Definitions, Grading & Relationship, Outcome and Follow-up 50
11.1.1 Definitions 50
11.1.2 Grading and Relationship 51
11.1.3 Outcome 54
11.1.4 AE Follow-Up 54
11.2 Serious Adverse Event Reporting 54
11.2.1 Definition of Serious Adverse Event 54
11.2.2 Serious Adverse Event Reporting Procedure 55
11.2.3 SAE Follow-Up 56
11.3 Pregnancy Reporting 56
12 KNOWN AND POTENTIAL RISKS AND BENEFITS TO HUMAN SUBJECTS 57

12.1 Benefits to Human Subjects 57
12.2 Risks Associated with Administration of TS-134 57
12.3 Risks Associated with Ketamine Administration 57
12.4 Risks Associated with MRI Scanning 58
13 INVESTIGATOR OBLIGATIONS 60
13.1 Ethical Considerations 60
13.2 Institutional Review Board (IRB) Approval 60
13.3 Informed Consent 60
13.3.1 Health Insurance Portability and Accountability (HIPAA) Authorization 61
13.3.1.1 Authorization Core Elements 61
13.3.1.2 Authorization Required Statements 62
13.4 Subject Confidentiality 62
13.5 Subject's Financial Responsibilities During the Study 62
14 DOCUMENTATION, RECORD KEEPING, AND DATA MANAGEMENT 63
14.1 Source Data/Documents 63
14.2 Records Retention 64
14.3 Data Management 65
15 CHANGES TO THE PROTOCOL AND STUDY TERMINATION 65
15.1 Study Design/ Procedure Modifications Permitted Within Protocol Parameters 65
15.2 Protocol Amendment 66
15.3 Protocol Termination 66
16 STUDY MONITORING 66
16.1 Clinical Monitoring 66
16.2 Auditing Procedures 67
17 REFERENCES 68

2 LIST OF ABBREVIATIONS

Term	Definition
**β-HCG**	Beta-Human Chorionic Gonadotropin
**2-DG**	2-deoxyglucose
**ACC**	Anterior Cingular Cortex
**AE**	Adverse Event
**ALP**	Alkaline Phosphatase
**ANCOVA**	Analysis of Covariance
**BMI**	Body Mass Index
**BOLD**	Blood Oxygen Level Dependent
**BP**	Blood Pressure
**bpm**	Beats Per Minute
**BPRS**	Brief Psychiatric Rating Scale
**BUN**	Blood Urea Nitrogen
**CADSS**	Clinician Administered Dissociative States Scale
**CFR**	Code of Federal Regulations
**CI**	Confidence Interval
**Cl**	Chloride
**cm**	Centimeters
**CNS**	Central Nervous System
**CPK**	Creatinine Phosphokinase
**CRA**	Clinical Research Associate
**CRF**	Case Report Form
**CSF**	Cerebrospinal Fluid
**C-SSRS**	Columbia- Suicide Severity Rating Scale
**CV**	Curriculum Vitae
**DBP**	Diastolic Blood Pressure
**DMP**	Data Management Plan
**DNA**	Deoxyribonucleic Acid
**ECG**	Electrocardiogram
**EPI**	Echo-Planar Imaging
**ET**	Early Termination
**FDA**	Food and Drug Administration
**fMRI**	Functional MRI

Term	Definition
**FOV**	Field of View
**g**	Grams
**GABA**	Gamma-aminobutyric acid
**GCP**	Good Clinical Practices
**GGT**	Gamma Glutamyl Transpeptidase
**h**	Hour
**HbsAg**	Hepatitis B Surface Antigen
**HCV**	Hepatitis C Virus
**HDL**	High-density Lipoprotein
**HIPAA**	Health Insurance Portability and Accountability Act
**HIV**	Human Immunodeficiency Virus
**HP-β-CD**	Hydroxypropyl-β-cyclodextrin
**ICF**	Informed Consent Form
**ICH**	International Conference on Harmonization
**IRB**	Institutional Review Board
**IUD**	Intrauterine Device
**K**	Potassium
**kg**	Kilograms
**LC-MS/MS**	Liquid Chromatography – Tandem Mass Spectrometry
**LDH**	Lactate Dehydrogenase
**LDL**	Low-density Lipoprotein
**m**	Meters
**MAD**	Multiple Ascending Dose
**MCHC**	Mean Corpuscular Hemoglobin Concentration
**MCV**	Mean Corpuscular Volume
**MDMA**	3,4-methylenedioxy-N-methylamphetamine
**MedDRA**	Medical Dictionary for Regulatory Activities
**MEM**	Memantine
**mg**	Milligrams
**Mg**	Magnesium

**mGlu**	Metabotropic glutamate
**min**	Minutes
**mL**	Milliliter
**mmHg**	Millimeters of Mercury
**msec**	Milliseconds
**MR**	Magnetic Resonance
**MRI**	Magnetic Resonance Imaging
**Na**	Sodium
**NDA**	New Drug Application
**NF**	National Formulary
**NKI**	Nathan S. Kline Institute
**NMDA**	*N*-methyl-$_{D}$-aspartate
**ºC**	Degrees Celsius
**PCP**	Phencyclidine
**PD**	Pharmacodynamics
**PGx**	pharmacogenomic
**pH**	Hydrogen Ion Concentration
**PHI**	Protected Health Information
**PK**	Pharmacokinetics
**POM**	Proof of Mechanism
**QTcF**	QT time interval corrected for heart rate using Fridericia's formula
**RBC**	Red Blood Cell
**RDW**	Red Cell Distribution Width

**RF**	Radiofrequency
**ROI**	Region of Interest
**rpm**	Revolutions Per Minute
**SAD**	Single Ascending Dose
**SAE**	Serious Adverse Event
**SAP**	Statistical Analysis Plan
**SAR**	Specific Absorption Ratio
**SBP**	Systolic Blood Pressure
**SCID-5-CT**	Structured Clinical Interview for DSM-5 Disorders – Clinical Trials Version
**SGPT**	Serum Glutamic-Pyruvic Transaminase
**SGOT**	Serum Glutamic-Oxaloacetic Transaminase
**SOP**	Standard Operating Procedure
**SPGR**	Spoiled Gradient Echo
**TE**	Echo Time
**TEAE**	Treatment Emergent Adverse Event
**THC**	Tetrahydrocannabinol
**t_{max}**	Time to Maximum Plasma Concentration
**TR**	Repetition Time
**USP**	United States Pharmacopeia
**WBC**	White Blood Cell

3 SYNOPSIS

**Protocol Title**	A randomized, single-blind, parallel-group study to evaluate the effects of TS-134 on ketamine-induced BOLD signals in resting fMRI in healthy adult subjects
**Protocol Number**	TS134-US103
**Phase of Development**	1b
**Number of Study Centers**	1 (US)
**Number of Subjects**	Approximately 60 healthy males and females between 18 and 55 years of age inclusive (3 groups: 25 subjects for a TS-134 20 mg group, 25 subjects for a TS-134 60 mg group and 10 subjects for a placebo group)
**Objectives**	The primary objective of this study is:<br>• To evaluate the treatment effects of TS-134 on ketamine-induced BOLD signals in pre-specified regions of interest (ROIs) in resting fMRI<br>The secondary objectives of this study are:<br>• To evaluate the treatment effects of TS-134 on ketamine-induced BOLD signals in whole brain in resting fMRI<br>• To evaluate the treatment effects of TS-134 on scores of the Brief Psychiatric Rating Scale (BPRS) and the Clinician Administered Dissociative States Scale (CADSS)<br>• To evaluate safety of multiple dose titrations of TS-134
**Study Design**	This study employs a randomized, single-blind, parallel-group design in healthy volunteers.<br>Following an up to 27-day outpatient Screening Period, eligible subjects will undergo an 8-day inpatient Treatment Period. Subjects will be randomized to one of three treatment groups in a ratio of 5:5:2 (25 subjects for a TS-134 20 mg group, 25 subjects for a TS-134 60 mg group and 10 subjects for a placebo group). During the study, each subject will undergo a total of two ketamine sessions; a first session during a Screening Period and a second session on Day 6 of a Treatment Period. Each ketamine session will include one ketamine infusion with resting fMRI scanning from pre to post ketamine infusions. First and second ketamine sessions will be conducted at least 7 days apart. All randomized subjects will be dosed with TS-134 or placebo daily in a fed state, for 6 days during the study. TS-134 dose levels will be titrated according to the titration schedule shown below. Subjects' general health and safety status will be confirmed by a phone call one week following discharge from a Treatment Period. The overall time to participate in this study is approximately 2 to 6 weeks.

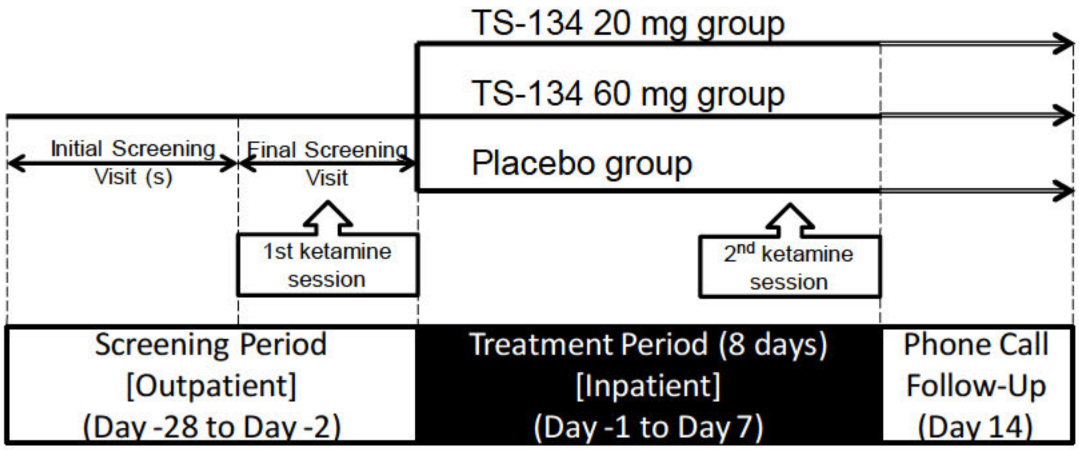

Treatment Titration Scheme:

Treatment Group	No. of Subjects	Treatment Period							
		Day -1	Day 1	Day 2	Day 3	Day 4	Day 5	Day 6	Day 7
TS-134 20 mg group	25	-	P	P	10 mg	10 mg	20 mg	20 mg	-
TS-134 60 mg group	25		10 mg	10 mg	20 mg	40 mg	60 mg	60 mg	
Placebo group	10		P	P	P	P	P	P	

P: placebo

4 BACKGROUND INFORMATION

Introduction

Schizophrenia is a chronic, severe, and devastating psychiatric disorder that affects 1% of the worldwide population. In the United States, approximately 2.2 million people have a diagnosis of schizophrenia[1]. It is ranked among the top 10 causes of disability and accounts for one-fourth of all mental health costs in the United States. The core symptoms of schizophrenia are characterized into 3 categories referred to as positive symptoms (hallucinations, delusions, agitation, disorganized thinking, etc.), negative symptoms (blunted affect, emotional withdrawal, apathetic social withdrawal, etc.), and cognitive dysfunction (poor memory, poor attention, executive dysfunction, etc.). While the positive symptoms can often be well managed using currently prescribed antipsychotics, the negative symptoms, and especially the cognitive dysfunction, remain unaffected by current medications[2]. The typical antipsychotics (first generation antipsychotics) also can induce undesirable side-effects such as extrapyramidal symptoms, including akathisia, dystonia, Parkinsonism and tardive dyskinesia. The atypical antipsychotics (second generation antipsychotics) are associated with fewer extrapyramidal symptoms, but have the risk of metabolic side-effects such as weight gain, hyperglycemia and hyperlipidemia. Thus, schizophrenia remains an unmet medical need requiring new drugs with improved efficacy and/or safety.

Mechanism of Action of mGlu2 and mGlu3 Receptor Agonists

Glutamate is an excitatory amino acid neurotransmitter in the mammalian central nervous system (CNS). Receptors for glutamate are categorized into 2 major classes: ionotropic glutamate receptors, which have an ion channel structure, and metabotropic glutamate (mGlu) receptors, which are coupled to G-proteins[3]. Among them, group II mGlu receptors have received considerable attention as possible drug targets based on their neuroanatomical localization and modulatory effects upon glutamatergic tone.

Both mGlu2 and mGlu3 receptors are abundantly distributed in the forebrain regions and limbic areas, including the cerebral cortex, hippocampus, striatum, nucleus accumbens, thalamus and amygdala, which are associated with neuropsychiatric disorders such as drug abuse, anxiety and schizophrenia[4,5,6,7]. These receptors localized on presynaptic terminals act as an autoreceptor and negatively regulate glutamate release[8]. Thus, the activation of mGlu2 and mGlu3 receptors is considered as an attractive strategy for treating the disorders associated with excessive glutamate neurotransmission.

Accumulating evidence suggests that hypofunction of prefrontal cortex and decreased NMDA receptor activation may play an important role in the pathophysiology of schizophrenia. Hypofunction of NMDA receptors located on GABAergic interneurons results in a disinhibition of pyramidal neurons and leads to an increase in glutamate release in the prefrontal cortex[9].

Since activation of mGlu2 and mGlu3 receptors presynaptically reduces the increased glutamate release, an agonist of mGlu2 and mGlu3 receptors could be a novel therapeutic target for schizophrenia.

A number of preclinical studies have indicated that modulation of glutamatergic activity in limbic and forebrain areas may have a therapeutic value as a novel mechanism for treating psychotic disorders, including schizophrenia, anxiety, depression, and addiction. The dissociative anesthetic NMDA receptor antagonists (such as PCP and ketamine) increase glutamate release in the prefrontal cortex and nucleus accumbens in rodents, producing abnormal behaviors that mimic symptoms associated with schizophrenia (such as hyperactivity, stereotypies and impaired performance on working memory tasks). Pretreatment of these animals with an mGlu2 and mGlu3 receptor agonist prevents the glutamate efflux and abolishes some PCP-induced abnormal behaviors[10]. Other potent mGlu2 and mGlu3 receptor agonists have been shown to attenuate PCP-evoked motor activity[11,12]. A clinical study reported that an mGlu2 and mGlu3 receptor agonist reversed ketamine-induced working memory deficits in healthy human subjects, indicating a potential therapeutic benefit in treating schizophrenia[13].

Such preclinical and clinical evidence suggests that mGlu2 and mGlu3 receptor agonists, with the function in reducing excessive glutamatergic neurotransmission in limbic and forebrain areas, could be useful in the treatment for schizophrenia.

4.1 Name and Description of Investigational Product

MGS0274 besylate is the active ingredient of TS-134 investigational product. MGS0274 is a prodrug, converted to the active metabolite MGS0008 upon enzymatic hydrolysis. MGS0008 is a selective agonist for mGlu2 and mGlu3 receptors. In all cases, dosed or measured mass of MGS0274 or MGS0008 are expressed as free base.

- Chemical Name: (1*S*,2*S*,3*S*,5*R*,6*S*)-2-Amino-3-fluoro-6-({(1*S*)-1-[({[(1*R*,2*S*,5*R*)-5-methyl-2-(propan-2-yl)cyclohexyl]oxy}carbonyl)oxy]ethoxy}carbonyl)bicyclo[3.1.0]hexane-2-carboxylic acid monobenzenesulfonate
- Molecular Formula: $C_{21}H_{32}FNO_7 \cdot C_6H_6O_3S$
- Molecular Weight: 587.65 (as besylate salt)

Additional details on investigational product including excipients can be found in the TS-134 Investigator's Brochure.

[redacted]

[redacted]

[redacted]

[redacted]

[redacted]

[redacted]

[redacted]

[redacted]

[redacted]

[redacted]

[redacted]

[redacted]

5 STUDY OBJECTIVES

5.1 Primary Objective

- To evaluate the treatment effects of TS-134 on ketamine-induced BOLD signals in pre-specified regions of interest (ROIs) in resting fMRI

5.2 Secondary Objectives

- To evaluate the treatment effects of TS-134 on ketamine-induced BOLD signals in whole brain in resting fMRI
- To evaluate the treatment effects of TS-134 on scores of the Brief Psychiatric Rating Scale (BPRS) and the Clinician Administered Dissociative States Scale (CADSS)
- To evaluate safety of multiple dose titrations of TS-134

6 STUDY DESIGN AND EVALUATION

6.1 Study Design

This study employs a randomized, single-blind, parallel-group design in healthy volunteers.

This study will enroll approximately 60 subjects. Following an up to 27-day outpatient Screening Period, eligible subjects will undergo an 8-day inpatient Treatment Period. Subjects will be randomized to one of three treatment groups in a ratio of 5:5:2 (25 subjects for a TS-134 20 mg group, 25 subjects for a TS-134 60 mg group and 10 subjects for a placebo group) (Figure 6-1). During the study, each subject will undergo a total of two ketamine sessions; a first session during a Screening Period and a second session on Day 6 of a Treatment Period. Each ketamine session will include one ketamine infusion with resting fMRI scanning from pre to post ketamine infusions. First and second ketamine sessions will be conducted at least 7 days apart. All randomized subjects will be dosed with TS-134 or placebo daily in a fed state, for 6 days during the study. TS-134 dose levels will be titrated according to the titration schedule shown in Table 6-1. Subjects' general health and safety status will be confirmed by a phone call one week following discharge from a Treatment Period.

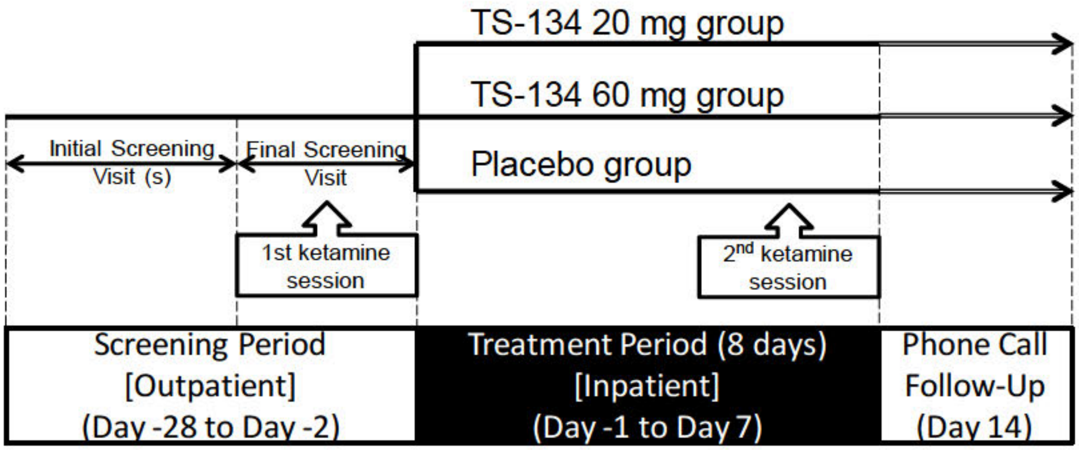

Figure 6-1 Study Design

Table 6-1 Treatment Titration Scheme

Treatment Group	No. of Subjects	Treatment Period							
		Day -1	Day 1	Day 2	Day 3	Day 4	Day 5	Day 6	Day 7
TS-134 20 mg group	25	-	P	P	10 mg	10 mg	20 mg	20 mg	-
TS-134 60 mg group	25		10 mg	10 mg	20 mg	40 mg	60 mg	60 mg	
Placebo group	10		P	P	P	P	P	P	

P = placebo

Premature Discontinuation:

Subjects may discontinue study participation at any time in this trial. All randomized subjects who prematurely discontinue participation after receiving at least one dose of investigational product will be followed-up by telephone by study staff approximately 1 week after their last dose administration (Early Termination (ET) Phone Call Follow-Up). Subjects who discontinue from the study prior to completion of the ketamine BOLD assessment on Day 6 may be replaced at the sponsor’s discretion. See Section 8.4 for further details pertaining to early discontinuation procedures.

6.2 Study Endpoints

6.2.1 Primary Endpoint

Changes in ketamine-induced BOLD fMRI signals in pre-specified ROIs (anterior cingular cortex (ACC) and anterior insula) following administrations of TS-134

6.2.2 Secondary Endpoints

- Changes in ketamine-induced BOLD fMRI signals in whole brain following administrations of TS-134
- Changes in BPRS scores following administrations of TS-134
- Changes in CADSS scores following administrations of TS-134
- Changes in safety parameters following administrations of TS-134

7 STUDY POPULATION

This study will be conducted in approximately 60 healthy male and female volunteers. These subjects are defined by the inclusion and exclusion criteria described in the following sections.

7.1 Inclusion Criteria

Subjects must meet all of the following inclusion criteria to be eligible for enrollment:

1. Healthy adult male and female subjects between 18 and 55 years of age inclusive (at the time of initial informed consent).
2. Body weight ≥ 45 and ≤ 87 kg at Screening Visits.
3. Body Mass Index (BMI) ≥ 18 and ≤ 30 kg/m^2 at Initial Screening Visit using the following formula:
   weight (kg) / $[\text{height (m)}]^2$.
4. Female subjects of child-bearing potential must agree to use one of the accepted barrier methods of contraception (listed below) during the trial (including the Screening Period), and at least 90 days after stopping the investigational product.
   a. condom (male or female) with spermicide,
   b. diaphragm or cervical cap with spermicide and condom (male),

   Note: Oral contraception, vasectomy of the partner and IUD alone are not considered sufficient contraception. One of the aforementioned methods listed above must be used. Females who are not currently sexually active must also consent to use one of these accepted methods of contraception should they become sexually active while participating in the study.

   Female subjects of non-childbearing potential must be either surgically sterile (i.e., documentation of hysterectomy or bilateral oophorectomy) or post-menopausal (absence of spontaneous menses for 12 consecutive months).
   Male subjects should refrain from fathering a child or donating sperm during the study and for at least 90 days following the last dose of TS-134.
5. The subject is able and willing to provide written informed consent and authorization for protected health information disclosure in accordance with Good Clinical Practice (GCP).

7.2 Exclusion Criteria

Subjects are to be excluded from study participation if they meet any of the following exclusion criteria:

1. Female subjects who are pregnant, intend to become pregnant (within 90 days of the last dose), or are breastfeeding. Positive serum/urine pregnancy test results at Screening Visits or Day -1 (Admission) will disqualify a subject from study participation.

2. Clinically significant abnormal values for hematology, biochemistry or urinalysis at the Initial Screening Visit. It is expected that the values will generally be within the normal range for the laboratory, however minor variances, where they are considered not to be clinically significant per the investigator, are acceptable.
3. Clinically significant abnormal physical examination, vital signs OR 12-lead electrocardiogram (ECG) at Screening Visits (except for Final Screening Visit: Discharge) or Day -1 (Admission).
4. Significant history or presence of hepatic, renal, cardiovascular, pulmonary, gastrointestinal, hematological, locomotor, immunologic, ophthalmologic, metabolic or oncological disease.
5. History or presence of psychiatric or neurologic disease or condition (including but not limited to any psychosis, schizophrenia, bipolar disorder, epilepsy, closed head trauma with unconsciousness, partial onset seizures etc.), confirmed through a Structured Clinical Interview for DSM-5 disorders – Clinical Trials Version (SCID-5-CT).
6. History of first-degree relative with schizophrenia or mood disorder with psychosis.
7. Recent history (within previous 6 months) of alcohol or drug abuse (as judged by the investigator) or has consumed > 2 alcohol drinks/day during the last 3 months prior to screening (1 glass is approximately equivalent to: beer [284 mL/ 10 ounces], wine [125 mL/ 4 ounces], or distilled spirits [25 mL/ 1 ounce]). Subjects that consume 3 glasses of alcoholic beverages per day, but less than 14 glasses per week, may be enrolled at the discretion of the investigator. Positive urine/saliva screen for alcohol or controlled substances at Screening Visits or Day -1 (Admission) will disqualify a subject from study participation.
8. History of recreational ketamine use, recreational PCP use, or an adverse reaction to ketamine. Subjects who have participated in prior research ketamine studies will be eligible providing they have participated in no more than 5 previous research ketamine infusions. Subjects can have infusions not more frequently than biweekly, and not more than 1/month on average, therefore subjects will need to wait 1 month if they had a single infusion and 6 weeks if they have had 2 closely spaced infusions prior to screening into the study.
9. Any subject who is unable to refrain from smoking throughout the study or with positive urine cotinine screen (> 400 ng/mL) at the Initial Screening Visit.
10. Any subject with a history and/or current evidence of serologic positive results for hepatitis B surface antigen, hepatitis C antibodies, or HIV antibodies 1 and 2 at the Initial Screening Visit.
11. History of violence, including any history of using a gun, knife, or other weapon with intent to harm someone, as well as a more than one physical fight without a weapon after the age of 18 years old (not including fights that happen during sports competition).

12. Presence or positive history of significant medical illness, including high blood pressure (SBP > 140 mmHg, DBP > 90 mmHg), low blood pressure (SBP < 100 mmHg, DBP < 60 mmHg), orthostatic hypotension (change in mean arterial pressure [1/3 systolic + 2/3 diastolic] of > 20%) at Screening Visits (except for Final Screening Visit: Discharge) or Day -1 (Admission).
13. Any subjects who show subthreshold ketamine BOLD response (defined as % BOLD signal change of 0.5% at peak ketamine response) in ACC at Final Screening Visit.
14. Metal implants, pacemaker, other metal (e.g. shrapnel or surgical prostheses) or paramagnetic objects contained within the body which may present a risk to the subject or interfere with the MR scan, as determined in consultation with a neuroradiologist and according to the guidelines set forth in the following reference book commonly used by neuroradiologists: *Guide to MR procedures and metallic objects, F. G. Shellock, Lippincott Williams and Wilkins Philadelphia 2001*[21].
15. Claustrophobia.
16. Donation of one or more units of blood, plasma, or acute loss of an equivalent amount of blood within 60 days prior to Initial Screening Visit. (One unit= 450mL)
17. Use of any prescription or over-the-counter medication (including medicinal patch), herbal medication, vitamins, or mineral supplements within 14 days prior to administration of the investigational product. Medicinal patch, if used during the Screening Period, must be removed prior to the MRI scan.

    Acetaminophen up to 3 g per day will be allowed, as well as non-steroidal topical preparations, eye drops for dry-eye that does not contain any active ingredient, oral contraceptives and hormonal replacement.
18. Any subject with previous exposure to the investigational product or who is known to be allergic to the investigational product or any components of the investigational product.
19. Any subject who has received treatment with a drug that has not received regulatory approval for an indication during the 30 days prior to screening.
20. Subject with any history of suicidal behavior or is currently at risk of suicide in the opinion of the investigator, or who has answered ‘YES’ to questions 1 or 2 on the Columbia - Suicide Severity Rating Scale (C-SSRS) at Final Screening Visit or Day -1 (Admission).
21. Participated in strenuous exercise within 48 hours prior to the initial dosing of the investigational product and/or are unwilling to avoid strenuous exercise at any time throughout the study.
22. Investigative site personnel or their immediate families (spouse, parent, child or sibling whether biological or legally adopted).
23. Taisho employee or their immediate families (spouse, parent, child or sibling whether biological or legally adopted).

24. Any subject not able to meet the study requirements.

8 TREATMENT OF SUBJECTS

8.1 Investigational Product

Physical characteristics of the investigational product are listed in the table below. A detailed description of the investigational product as well as a listing of excipients is included in the TS-134 Investigator's Brochure.

Table 8-1 Investigational Product Description

Investigational drug product solution *	Description
TS-134 drug product solution	Clear and colorless oral solution composed of 0.25 or 2.5 mg/mL of MGS0274 besylate (dose calculated as free base), HP-β-CD (solubilizing agent) and purified water.
TS-134 placebo solution<br>(matching placebo of TS-134 drug product solution)	Clear and colorless oral solution composed of Denatonium Benzoate (bittering agent), HP-β-CD (solubilizing agent), citric acid (pH adjustment) and purified water.

* Compounding of investigational product will be prepared at the study site. Active and placebo dosing solutions will be compounded to have a pH level no lower than 2.2.

8.1.1 Packaging and Labeling

MGS0274 besylate powder and HP-β-CD powder, USP, are supplied in bulk to be prepared as an oral solution at the study site for dosing. Each is provided in double low-density polyethylene bags, closed with a cable tie, as a primary container; a resealable, laminated-aluminum pouch is provided as a secondary container.

The study site will provide Denatonium Benzoate, NF, citric acid and Purified Water, USP.

The drug product solutions prepared at the study site will be labeled locally and stored in glass bottles with a cap.

8.1.2 Handling and Dispensing of Investigational Product

Investigational drug product components (powders) and all drug product solutions prepared at the study site should be stored at 5°C ± 3°C. Following preparation of each dose-level-specific dosing solution, a retention sample will be prepared and stored for possible shipment to the sponsor. Detailed instructions for preparing and handling TS-134 and placebo oral solutions and any retention samples will be provided in a separate pharmacy manual.

TS-134 and placebo will be administered orally as a solution, along with 240 mL of water. If subjects are not able to drink 240 mL of water completely, subjects' actual water intake will be recorded in the subjects' study records. The investigational product solution will be administered daily (every 24 hours) beginning on the morning of Day 1 and continue through Day 6, within 15 minutes after completing a standard breakfast. Water will be permitted *ad libitum* except for 1 hour before and 1 hour after each dose. A taste-masking product (e.g. Listerine PocketPaks breath strips) will be used immediately before and after daily dosing.

8.1.3 Randomization Procedure

A randomization schedule will be generated by the study site statistician to facilitate the randomization of subjects into each treatment group. Subjects will be randomly allocated to either TS-134 20 mg group, TS-134 60 mg group, or placebo group in a 5:5:2 ratio, respectively.

In addition to a screening number assigned to subjects at the time of informed consent, a unique subject number will be assigned to each subject at the time of randomization. This assigned subject number will correspond to a drug dispensed labeled with that subject number. Subjects will retain their subject number for the duration of the study.

Drug supplied by the sponsor in unblinded bulk supply containers will be dispensed by the pharmacist according to the randomization schedule and the respective titration schedule. Based on this randomization code, the unblinded pharmacist or properly trained designee will prepare the investigational product supplies by dispensing the designated investigational product for each subject into a container, which will be labeled with the appropriate study number and subject number.

8.1.4 Blinding

This study is a single-blind study and all subjects are blinded to the treatment assignment information. The investigator (except for blinded investigators described below) and the sponsor will have knowledge of the treatment assignment.

Blinded investigators:

- Investigators and/or trained designees who are responsible for administering and scoring of behavioral assessments described in Section 9.3.5.2 will be blinded from treatment assignment information until the study database is locked.
- Investigators and/or trained designees who are responsible for BOLD fMRI data scoring will be blinded from treatment assignment information until the database is locked.

8.1.5 Treatment Compliance

Responsible study personnel will dispense the investigational product. Accountability and

compliance verification should be documented in the subject's study records.

8.1.6 Investigational Product Accountability

The investigator is responsible for ensuring that all investigational products received at the site are inventoried and accounted for throughout the study. The dispensing of investigational product (TS-134 and placebo) must be documented on a drug accountability form which should include:

- Amount of dry bulk drug supplies received.
- Amount of dry bulk drug supplies currently stored.
- Label ID number or batch number.
- Amount of dry bulk drug supplies used for preparation of dosing solutions (for dosing and back-up)
- Dates and initials of person(s) responsible for each study medication inventory entry/movement.
- Amount of investigational product solutions prepared and amounts dispensed to each subject, including unique subject identifiers.
- Non-study disposition of dry bulk drug supplies and investigational drug solutions (e.g. lost, wasted, broken, unused back-up).
- Amount returned to sponsor or sponsor's authorized designee, or destroyed.

The investigator will retain and store all original containers until these containers are inventoried by the sponsor or sponsor's authorized designee.

8.1.7 Return and Destruction of Investigational Product

At the end of the study, all unused, partially used, or unopened containers of dry bulk drug supplies and retention samples of the extemporaneously-prepared investigational drug solutions will be returned to the sponsor or the sponsor's designee for destruction.

8.2 Concomitant Drug Therapy

Subjects are prohibited from taking any prescription, over-the-counter (including medicinal patch) or herbal medication, vitamins or mineral supplements within 14 days prior to administration of their investigational product, and for the duration of the study. A medicinal patch, if used during the Screening Period, must be removed prior to the MRI scan. The exception of racemic ketamine and saline used for the study procedure, acetaminophen in the amount of up to 3g per day, non-steroidal topical preparations, eye drops for dry eye that does

not contain any active ingredient, as well as oral contraceptives or hormonal replacement will be allowed.

If the subject requires any concomitant therapy for an adverse event, it must be reported in the subject's study records. Any changes to the concomitant therapy during the study must be clearly recorded and the reason for the change should be documented.

8.3 Duration of Subject Participation

This study is comprised of the following:

- Screening Period of up to 27 days (Day -28 to Day -2)
- Treatment Period of 8 days (Day -1 to Day 7)
- Phone Call Follow-up on Day 14

Subjects will make 3 visits (2 outpatient visits during a Screening Period and 1 inpatient visit for a Treatment Period) to the study site. The overall time to participate in this study is approximately 2 to 6 weeks.

8.4 Discontinuation Criteria and Procedures

In accordance with the Declaration of Helsinki, ICH GCP Guidelines, and the United States Food and Drug Administration (FDA) Regulations, a subject has the right to withdraw from the study at any time for any reason without prejudice to his/her future medical care by the physician or at the institution.

The investigator and sponsor also have the right to withdraw subjects from the study for reasons including the following:

- significant protocol violation on the part of the investigator or subject;
- unblinding of the subject, or designated unblinded investigator;
- significant noncompliance on the part of the subject;
- self-withdrawal, or refusal by the subject to continue treatment or observations;
- unacceptable toxicity;
- decision by the investigator that termination is in the subject's best medical interest and/or for safety reasons;
- unrelated medical illness or complication(s);
- sponsor decision to discontinue the study.

The sponsor may choose to terminate the study at any time for any reason. Should a subject decide to withdraw, all efforts will be made to complete and report the observations as

thoroughly as possible.

Should a subject discontinue prior to completion of the study, a final evaluation should be made at the time of the subject's withdrawal according to the procedures outlined in Sections 9.2.2.4 and 9.2.4. The reason for the withdrawal should be recorded and an Early Termination (ET) Phone Call Follow-Up evaluation approximately 1 week after the last dose of investigational product should be conducted. In the event that the subject cannot be contacted by phone, the site should make all necessary attempts to contact the subject to follow any adverse events.

9 STUDY PROCEDURES AND OBSERVATIONS

9.1 Events Schedule

Table 9-1 Schedule of Events

	Screening Period								Treatment Period											Phone Call Follow-Up (Day 14 ± 2 days) /ET Phone Call Follow-Up[e]
	Outpatient (Day -28 to Day -2)								Inpatient (Day -1 to Day 7)											
	Initial Screening Visit(s)	Final Screening Visit (First Ketamine Session)							Day -1 (Admission)	Day 1 to Day 5	Day 6 (End of Treatment) (Second Ketamine Session)								Day 7 (Discharge) /ET	
		Before behavioral assessments	-2h	-25 min	0 min	15 min	20 min	Discharge			Pre-infusion	0h	3h	6h	7h 35min	8h	8h 15min	8h 20min		
Informed Consent	X																			
SCID-5-CT	X																			
Inclusion/Exclusion Criteria	X	X							X											
Subject Demographics	X																			
Medical and Psychiatric History	X																			
Prior Medication History	X	X							X											
Height	X																			
Body Weight	X	X									X									
BMI	X																			
Serology Screen	X																			
Urine Cotinine Screen	X																			
Saliva Alcohol Screen	X	X							X											
Urine Drug Screen	X	X							X											
Serum[s]/Urine[u] Pregnancy Test (all female subjects)	X [s]	X [u]							X [u]											
Physical Examination (including a neurological examination)	X	X						X	X		X								X	
Vital Signs (BP, oral temperature)	X	X						X	X	Day 3	X								X	
12-Lead ECG	X	X						X	X	Day 3	X								X	
C-SSRS		X							X				X							
Clinical Laboratory Test (Hematology, Biochemistry, Urinalysis)	X								X										X	
PGx Sample Collection		X																		
Randomization										Day 1										
Administration of Investigational Product										X		X								
PK Sample Collection (MGS0008)													X							
Ketamine Infusion[a]					X											X				
MRI scan	(mock)[c]			X--	----	--X									X--	----	--X			
Continuous ECG, Pulse Oximeter, BP monitoring				X--	----	--X									X--	----	--X			
PK Sample Collection (ketamine)							X											X		
BPRS			X				X[d]							X				X[d]		
CADSS			X				X[d]							X				X[d]		
Phone Follow-Up																				X
AE Reporting	X--	----	----	----	----	----	----	----	----	----	----	----	----	----	----	----	----	----	----	--X
Concomitant Medication Inquiry & Reporting[b]	(X--	----	----	----	----	----	----	----	----)	X--	----	----	----	----	----	----	----	----	--X	

[a] Infused as a bolus over 1 min.

[b] All concomitant medications will be reported from the time of initial administration of the investigational product at Day 1 until discharge at Day 7.
Any concomitant therapy for an AE occuring prior to the initial administration of the investigational product will be reported from the onset of the AE.

[c] Mock scan can be arranged, if needed.

[d] Immediately following the collection of PK sample for ketamine.

[e] Approximately 1 week after the last dose.

9.2 Procedures by Visit

A schedule of the required study procedures and evaluations are outlined in Sections 9.1 and 9.2. Every effort should be made to complete the required procedures and evaluations at the designated visits and times (see Section 9.4 for allowable time tolerances).

9.2.1 Screening Period (Day -28 to Day -2)

Subjects will report to the study site for eligibility screening in between Day -28 and Day -2, inclusive. Before any study specific procedures are conducted, the subject must sign a written Informed Consent Form (ICF).

9.2.1.1 Initial Screening Visit(s)

During this visit, the following study procedures will be administered to each subject:

- Informed consent
- SCID-5-CT
- Review of inclusion and exclusion criteria
- Subject demographics
- Review of medical and psychiatric history
- Review of prior medication history
- Height (cm)
- BMI
- Body weight (kg)
- Physical examination (including a neurological examination)
- Vital signs
- 12-Lead ECG
- Serology screen (for Hepatitis B surface antigen (HBsAg), serum antibody against Hepatitis C virus (anti-HCV antibody) and Human Immunodeficiency Virus (HIV))
- Urine cotinine screen
- Saliva alcohol screen
- Urine drug screen
- Serum pregnancy test (all female subjects)
- Clinical laboratory tests (hematology, biochemistry, and urinalysis)

- Ongoing AE surveillance
- Ongoing concomitant medication for AE inquiry & reporting

A mock MRI scan session can be arranged to acclimate subjects to the MRI scan environment and to rule out subjects who are claustrophobic or uncomfortable to stay inside the MRI scanner, if needed.

9.2.1.2 Final Screening Visit (First Ketamine Session)

Following the Initial Screening Visit(s), subjects who meet the initial enrollment criteria will report to the study site for a one-day Final Screening Visit.

During this visit, the following study procedures will be administered to each subject:

- Review of inclusion and exclusion criteria
- Review of prior medication history
- Body weight (kg)
    - Before behavioral assessments
- Physical examination (including a neurological examination)
    - Before behavioral assessments, Discharge
- Vital signs
    - Before behavioral assessments, Discharge
- 12-Lead ECG
    - Before behavioral assessments, Discharge
- C-SSRS – Baseline version
    - Before behavioral assessments
- Saliva alcohol screen
    - Before behavioral assessments
- Urine drug screen
    - Before behavioral assessments
- Urine pregnancy test (all female subjects)
    - Before behavioral assessments
- Ketamine infusion
    - 0 min

- MRI scan
  - 25 min prior to initiation of ketamine infusion: a 7-minute structural scan, a 2-minute phase map, and 2 sets of 30-second BOLD top-up scans
  - 15 min prior to initiation of ketamine infusion: 30-minute BOLD fMRI scan including pre-ketamine (15 minutes) and post-ketamine (15 minutes) scan
- Continuous ECG, pulse oximeter, blood pressure (BP) monitoring
  - throughout the MRI scan
- PK sample collection (ketamine)
  - 5 min post cessation of MRI scan
- BPRS
  - pre-ketamine: 2h prior to initiation of ketamine infusion
  - post-ketamine: immediately following the collection of PK sample for ketamine
- CADSS
  - pre-ketamine: 2h prior to initiation of ketamine infusion
  - post-ketamine: immediately following the collection of PK sample for ketamine
- PGx sample collection (optional)
- Ongoing AE surveillance
- Ongoing concomitant medication for AE inquiry & reporting

Subjects will be discharged following completion of safety procedures described above.

9.2.2 Treatment Period (Day -1 to Day 7)

Following the Screening Period, subjects who meet the enrollment criteria will be admitted to the site on Day -1. Subjects will remain in the site during the Treatment Period. The following study procedures will be performed on Day -1 through Day 7.

9.2.2.1 Day -1 (Admission)

- Admission to site
- Review of inclusion and exclusion criteria
- Review of prior medication history
- Physical examination (including a neurological examination)
- Vital signs

- 12-Lead ECG
- C-SSRS – Since Last Visit version
- Saliva alcohol screen
- Urine drug screen
- Urine pregnancy test (all female subjects)
- Clinical laboratory tests (hematology, biochemistry, and urinalysis)
- Ongoing AE surveillance
- Ongoing concomitant medication for AE inquiry & reporting

9.2.2.2 Day 1 to Day 5

- Randomization (Day 1: prior to initial administration of investigational product)
- Vital signs (Day 3 only)
- 12-Lead ECG (Day 3 only)
- Administration of investigational product
- Ongoing AE surveillance
- Ongoing concomitant medication inquiry & reporting

9.2.2.3 Day 6 (End of Treatment: Second Ketamine Session)

- Body weight (kg)
  - Pre-infusion
- Physical examination (including a neurological examination)
  - Pre-infusion
- Vital signs
  - Pre-infusion
- 12-Lead ECG
  - Pre-infusion
- C-SSRS – Since Last Visit version
  - 3h post administration of investigational product
- Administration of investigational product
  - 0h

- PK sample collection (MGS0008)
  - 3h post administration of investigational product
- Ketamine infusion
  - 8h post administration of investigational product
- MRI scan
  - 7h 35 min post administration of investigational product: a 7-minute structural scan, a 2-minute phase map, and 2 sets of 30-second BOLD top-up scans
  - 7h 45 min post administration of investigational product: 30-minutes BOLD fMRI scan including pre-ketamine (15 minutes) and post-ketamine (15 minutes) scan
- Continuous ECG, pulse oximeter, BP monitoring
  - throughout the MRI scan
- PK sample collection (ketamine)
  - 5 min post cessation of MRI scan
- BPRS
  - pre-ketamine: 6h post administration of investigational product
  - post-ketamine: immediately following the collection of PK sample for ketamine
- CADSS
  - pre-ketamine: 6h post administration of investigational product
  - post-ketamine: immediately following the collection of PK sample for ketamine
- Ongoing AE surveillance
- Ongoing concomitant medication inquiry & reporting

9.2.2.4 Day 7 (Discharge) / Early Termination (ET)

Subjects will be discharged on Day 7 following completion of the procedures listed below:

- Physical examination (including a neurological examination)
- Vital signs
- 12-Lead ECG
- Clinical laboratory tests (hematology, biochemistry, and urinalysis)
- Ongoing AE surveillance

- Ongoing concomitant medication inquiry & reporting

Subjects who discontinue prematurely after receiving at least one dose of investigational product will complete the same Day 7 procedures before discharge.

9.2.3 Phone Call Follow-Up (Day 14)

Subjects will be followed up by telephone by study staff on Day 14 to confirm general health and safety status, including any possible AEs. If the investigator deems follow-up safety assessments are required considering the AE, subjects will return to the study site for additional safety assessments.

9.2.4 Early Termination (ET) Phone Call Follow-Up

Subjects who discontinue prematurely after receiving at least one dose of investigational product will be followed-up by telephone by study staff approximately 1 week after the date of last dose to confirm general health and safety status, including any possible AEs. If the investigator deems follow-up safety assessments are required considering the AE, subjects will return to the study site for additional safety assessments.

9.3 Study Procedures

9.3.1 Study Materials

Study documents (e.g. Case Report Forms (CRFs), drug accountability logs, etc.) will be provided by the sponsor, the study site and/or a contracted designee. Specimen collection supplies (e.g. collection tubes and requisitions) for clinical assessments will be provided by the site or clinical safety laboratory.

9.3.2 Subject Background Assessments

9.3.2.1 Demographics & Medical/ Psychiatric History

A complete medical and psychiatric history will be obtained from subjects at the times indicated in Sections 9.1 and 9.2. Demographic information, including date of birth, gender, race and ethnicity will be recorded as well. Any new medical conditions reported prior to initial informed consent will be reported on the Medical History section of the subject study records while new updates after initial informed consent will be captured as AE.

9.3.2.2 Structured Clinical Interview for DSM-5 Disorders – Clinical Trials Version (SCID-5-CT)

The SCID-5-CT will be administered and reviewed by a qualified clinician or rater at the Initial Screening Visit to confirm a subject’s study eligibility. Results are to be included in the subject’s

study record.

9.3.2.3 Height

Height will be measured at the Initial Screening Visit only. The height measurement should be recorded in centimeters (cm) in the subject's study records.

9.3.2.4 Body Weight

Body weight will be measured at specified timepoints indicated in Sections 9.1 and 9.2. Results of the body weight are to be recorded in kilograms (kg) in the subject's study records.

9.3.2.5 Body Mass Index (BMI)

BMI will be calculated and recorded at the Initial Screening Visit only using the following formula: Weight (kg) / [height (m)]^2

9.3.3 Safety Assessments

9.3.3.1 Physical Examination (including a neurological examination)

A physical examination (including a neurological examination) will be performed at specified timepoints indicated in Sections 9.1 and 9.2. The physical examination will include, but not be limited to, assessments of the following:

- General Appearance
- Head/ Face
- Eyes
- Ears/Hearing
- Nose
- Mouth, Teeth and Throat
- Neck & Thyroid
- Chest/Lungs
- Abdomen
- Skin, Hair, and Nails
- Musculoskeletal: Extremities, Spine
- Vascular/Circulatory
- Lymphatic
- Psychiatric/Behavior
- Neurologic (see below)

The neurological examination will include assessments of the following:

- Cranial Nerves Function: Reactivity to Light, Accommodation of pupils, and Extraocular Eye Movements
- Coordination: Finger to Nose, Finger Tapping, Rapid alternating Hand Movements, Gait, and Romberg Test
- Deep Tendon Reflexes: Brachioradialis, Patellar, and Achilles
- Presence of Tremors: Resting, Postural, and Intension

- Sensory Exam: Pin Prick and Tactile Soft Touch
- Motor System: Spastic gait, Cerebellar ataxia, Sensory ataxia, Akinetic-rigid gait, Step age gait, Upper/Lower body strength
- Mental Status

The results of the physical examination are to be recorded in the subject's study records. Any clinically significant changes from baseline should be recorded as an AE.

9.3.3.2 Vital Signs

Vitals signs will be collected at specified timepoints indicated in Sections 9.1 and 9.2.

Vital signs will include oral body temperature (ºC), supine respiration rate, systolic and diastolic blood pressure (SBP, DBP), and heart rate, as well as standing SBP, DBP and heart rate. The supine BP and heart rate should be measured first after the subject has been lying down for ≥ 5 minutes. The subject should then be asked to stand and the standing BP and heart rate should be measured after the subject has been standing for at least 2 minutes. Vital signs are to be recorded in the subject's study records.

Criterion for orthostatic hypotension is defined as a 20% increase of the mean arterial pressure.

The following measurements for vital signs are considered normal ranges for this study. It is expected that the values will generally be within the normal range as indicated below, however minor deviations, where they are not considered to be clinically significant as per the investigator, are acceptable.

SBP	90 –139 mmHg
DBP	45 – 89 mmHg
Heart Rate	40 –100 bpm
Respiration Rate	16 – 20 breaths per min.
Temperature (oral)	33.3 – 37.7ºC

9.3.3.3 Electrocardiogram

All 12-lead ECGs will be complete, standardized recordings performed at specified timepoints indicated in Sections 9.1 and 9.2. The subject must rest in a supine position for ≥ 5 minutes before the ECG is obtained. ECG tracings (paper or electronic) will be reviewed and interpreted by a qualified clinician. ECG tracings and results (ventricular rate, PR, QRS, QT, QTcF intervals and investigator's interpretation) are to be included in the subject's study records.

9.3.3.4 Columbia - Suicide Severity Rating Scale (C-SSRS)

Administration and review of the C-SSRS will be performed by a qualified clinician at the specified timepoints indicated in Sections 9.1 and 9.2. C-SSRS (Baseline, version 1/14/09) will be administered at the Final Screening Visit and C-SSRS (Since Last Visit, version 1/14/09) will be administered thereafter. Results are to be included in the subject’s study records. In the opinion of the investigator, any subjects who pose a serious safety risk to themselves will be managed according to the study site’s Standard Operating Procedures (SOPs) for follow-up treatment and reporting.

9.3.4 Clinical Laboratory Tests

Table 9-2 summarizes the laboratory tests to be evaluated in the study. All laboratory reports must be reviewed by the investigator, and any abnormalities must be assessed for clinical significance. Results are to be included in the subject’s study records.

Table 9-2 Summary of Clinical Laboratory Test

Hematology	Biochemistry	Urinalysis	Serology
Red Blood Cell count (RBC) White Blood Cell count (WBC) WBC differential	Total Protein	Hydrogen ion concentration (pH)	Hepatitis B surface antigen
Hemoglobin	Albumin	Specific gravity	HIV
Hematocrit	Calcium	Protein	Hepatitis C antibody
Mean Corpuscular Hemoglobin (MCH)	Inorganic phosphorus	Glucose	
Mean Corpuscular Hemoglobin Concentration (MCHC)	Cholesterol (total, LDL, HDL)	Ketones	**Pregnancy**
Red cell distribution width (RDW)	Triglycerides	Leukocyte esterase	Serum/urine pregnancy (β-HCG)
Mean Corpuscular Volume (MCV)	Serum glutamic-pyruvic transaminase (SGPT/ALT)	Nitrites	
Platelet count	Serum glutamic-oxaloacetic transaminase (SGOT/AST)	Bacteria	**Toxicology**
Reticulocyte count	Electrolytes (Na, K, Mg, Cl) and Bicarbonate	Occult blood	Saliva alcohol screen
	Blood Urea Nitrogen (BUN)	WBC/high-power field	Urine cotinine screen
	Blood glucose	Crystals	Urine drug screen *(amphetamine, cocaine, barbiturates, methadone, benzodiazepines, MDMA morphine/opiates, PCP, methamphetamine, THC)*
	Uric Acid	Casts	
	Total and direct bilirubin	Epithelial cells	
	Alkaline phosphatase (ALP)	Mucous Thread	
	Lactate dehydrogenase (LDH)		
	Gamma Glutamyl Transpeptidase (GGT)		
	Creatinine Phosphokinase (CPK)		
	Serum Creatinine		

9.3.4.1 Serology Screen

A blood sample will be collected at the Initial Screening Visit for Hepatitis B surface antigen, HIV screening and serum antibodies to hepatitis C. In the event that the HIV results are positive, the study site must follow appropriate study site specific SOPs for informing the subject of their status, follow-up treatment and reporting.

9.3.4.2 Urine Cotinine Screen

A urine sample will be collected at the Initial Screening Visit to screen for tobacco use.

9.3.4.3 Saliva Alcohol Screen

Saliva alcohol screens will be performed at specified timepoints indicated in Sections 9.1 and 9.2 to test for alcohol levels.

9.3.4.4 Urine Drug Screen

A urine sample will be collected at specified timepoints indicated in Sections 9.1 and 9.2 to

screen for controlled substances. The following controlled substances will be tested: amphetamine, barbiturates, benzodiazepines, cocaine, methadone, morphine/opiates, methamphetamine, marijuana (THC), phencyclidine (PCP), ecstasy (MDMA).

9.3.4.5 Serum/Urine Pregnancy

For all female participants, serum/urine pregnancy samples will be drawn at specified timepoints indicated in Sections 9.1 and 9.2.

9.3.4.6 Hematology and Biochemistry

Blood samples will be drawn preferably under fasting conditions for routine hematology and biochemistry (see Table 9-2) at specified timepoints indicated in Sections 9.1 and 9.2.

9.3.4.7 Urinalysis

Urine samples will be collected for routine urinalysis at specified timepoints indicated in Sections 9.1 and 9.2.

9.3.5 Pharmacodynamics (PD) Assessments

9.3.5.1 MRI Assessments with Ketamine Infusion

BOLD fMRI assessments with ketamine infusion will be conducted at specified timepoints indicated in Sections 9.1 and 9.2. The fMRI scanning time at the second session should be within ± 2 hour of the scanning time at the first session.

Subjects will be under close observation by an investigator, with visual and verbal contact maintained throughout the MRI session. Subjects who experience distress or become claustrophobic in the magnet will be removed immediately from the magnet room. At least one physician will be present during the course of the procedure. All staff involved in conduct of MRI scan will be thoroughly familiar with the study site’s emergency plan.

Total duration of MRI scanning will be approximately 40 minutes. The scanning will be comprised of a 10-minute structural MRI scan (a 7-minute structural scan, a 2-minute phase map, and 2 sets of 30-second BOLD top-up scans), a 15-minute pre-ketamine BOLD fMRI scan, and a 15-minute post-ketamine BOLD fMRI scan. During the 15-minute pre-ketamine BOLD fMRI scan, subjects will receive continuous intravenous administration (i.e., drip) of saline to keep their intravenous line open for subsequent ketamine infusion. Subjects will be under constant monitoring by ECG and pulse oximeter, as well as by frequent BP measurements throughout the MRI scan, which will be recorded in the subject’s study records. The make and model of the MRI scanner used, date and time of initiation and cessation of MRI scanning are to be recorded in the subject’s study record.

9.3.5.1.1 Ketamine Infusion

Subjects will be administered with a racemic ketamine hydrochloride intravenous constant infusion. Ketamine will be infused as 0.23 mg/kg bolus over 1 minute in accordance with the subject's weight (measured at each ketamine session day) inside the MRI scanner. Ketamine dosage should not exceed 20 mg per subject per session.

Ketamine infusion and subsequent 15-minute fMRI scan will be performed in the presence of the investigator. The date and time of initiation of ketamine infusion are to be recorded in the subject's study record.

9.3.5.1.2 Image Acquisition

A MRI manual is being provided in a separate document to take precedence over the protocol language in this section. Any revisions to the MRI manual that are implemented during the study will be described in the final report.

Subjects will be scanned using a 3.0T MRI scanner. An approximate 40-minute, eyes-open, resting-state BOLD fMRI scan will be acquired using echo-planar imaging (EPI). A total of 900 image volumes of 23 near-axial slices (4-mm thickness, aligned to the anterior commissure-posterior commissure) will be acquired per session; echo time (TE) = 30 msec; repetition time (TR) = 2000 msec; flip angle = 75°, in-plane resolution = 3.3 mm; matrix size = 64 x 64; field of view (FOV) = 21.1 x 21.1 cm. A T1 weighted SPGR scan will also be acquired (TE = 2.3msec, TR = 2400 msec, flip angle = 12°, 0.8 mm isotropic voxels, matrix size = 300 x 300, FOV = 24 x 24 cm).

9.3.5.1.3 Image Processing and Modeling

A MRI manual is being provided in a separate document to take precedence over the protocol language in this section. Any revisions to the MRI manual that are implemented during the study will be described in the final report.

No treatment identifiable information is encoded in the imaging data files and all MRI image analyses will be blinded to treatement condition. The data manager will maintain the randomization table for each imaging treatment assignment set until study database is locked.

fMRI data will be preprocessed using FSL (https://fsl.fmrib.ox.ac.uk/fsl/fslwiki) and Matlab (https://www.mathworks.com/). Preprocessing of fMRI data includes slice time correction, motion correction, spatial smoothing (5 mm full width at half maximum Gaussian kernel), and high-pass filtering with a cutoff of 1200 s will be applied to the data to minimize the influence of very-low-frequency noise and scanner drift. Linear regression is performed using 27 motion parameters including 3 translations, 3 rotations, lag +1 derivatives, lag -1 derivatives, quadratic term, the mean relative motion, and the mean absolute motion. The model residual is then used

for evaluating all ketamine-evoked responses. Linear registration to the subject's high resolution SPGR image (6 degrees of freedom) and to the MNI152 standard space template (12 degrees of freedom) is performed to generate spatial transforms.

A ROI analysis is performed using the mid-cingulate cortex, which had the largest BOLD response at 50 ng/mL ketamine in the study conducted by De Simoni[18]. Two seeds are placed at (MNI: 4,2,42) and (MNI: -4,2,42) and spherically dilated by 8 mm. The masks are transformed to the individual subject's functional space and multiplied by the gray matter partial volume map to minimize the contribution of non-gray voxels. For each subject, the BOLD time series is extracted from the ROI and fit to a three parameter gamma model:

$$y(t) = a\left(\frac{t}{t_{max}}\right)^{b \cdot t_{max}} e^{(t_{max}-t) \cdot b}$$

where t_{max} is time of maximum response, *a* is amplitude of the response, and *b* is the shape parameter of the function. The fitted response is converted to a percent signal change by normalizing to the mean. Peak BOLD response is defined as the maximum value of the fitted gamma model. Mean BOLD response is defined as the area under the curve of the gamma model divided by the scan duration (600 sec). The mean BOLD response is highly correlated to the peak BOLD response but is less sensitive to outliers when comparing groups. A whole brain analysis is performed using multiple linear regression by constructing a gamma regressor with parameters equal to the group mean (b = 0.009, t_{max} = 226 sec). The beta images from the contrast of the first regressor will be used in the group-level analyses.

9.3.5.2 Behavioral Assessments

The investigator or a trained designee who is responsible for behavioral assessment scoring will be blinded from any treatment assignment information until the study database is locked.

9.3.5.2.1 Brief Psychiatric Rating Scale (BPRS)

BPRS is a clinician administered rating scale of 20 items that assesses common psychiatric symptoms such as depression, anxiety, and psychotic symptoms[22]. The assessment takes approximately 20-30 minutes to administer.

BPRS will be administered by the investigator or a trained designee at specified timepoints indicated in Sections 9.1 and 9.2. Subjects will be rated outside of the MRI scanner for all timepoints. For post-ketamine ratings, subjects will be instructed to answer questions with regards to their experience during the resting BOLD fMRI scan. Results are to be included in the subject's study record.

9.3.5.2.2 Clinician Administered Dissociative States Scale (CADSS)

CADSS is a clinician administered rating scale that assesses dissociative symptoms[23]. Twenty-three subjective items, rated 0-4 will be assessed. The assessment takes approximately 5 minutes to administer.

CADSS will be administered by the investigator or a trained designee at specified timepoints indicated in Sections 9.1 and 9.2. Subjects will be rated outside of the MRI scanner for all timepoints. For post-ketamine ratings, subjects will be instructed to answer questions with regards to their experience during the resting BOLD fMRI scan. Results are to be included in the subject's study record.

9.3.6 Assessments of Pharmacokinetics (PK)

9.3.6.1 MGS0008

Plasma samples will be analyzed to determine the concentration of MGS0008 using a validated, specific and sensitive liquid chromatography - tandem mass spectrometry (LC-MS/MS) method. A description of the bioanalytical methods will be included in the final report. The determination of MGS0008 will be performed by [redacted]

9.3.6.1.1 Blood Sampling (MGS0008)

Blood samples for determination of PK of MGS0008 will be collected at Day 6, 3 hours post administration of investigational product. The date and time of blood sampling and the last investigational product administration before blood sampling must be recorded in the subject's study records.

<u>Blood collection tubes:</u> 10 mL plastic vacuum tubes containing heparin sodium.

<u>Plasma sample storage vessels:</u> 2.0 mL polypropylene cryovials

<u>Labeling:</u> The labels on tubes for plasma samples should include: the protocol number, subject number, study day, nominal time point, sample type (e.g., MGS0008 PK Plasma), aliquot type (Primary or Backup), and barcode / barcode number (if applicable).

After collection, the blood sample will be kept in an ice bath (or other cooling systems, except for refrigerator) until centrifuged. Sample must be centrifuged (4ºC, 3000 rpm, 10 min.) within 30 minutes after blood collection to obtain plasma. After centrifugation, plasma sample shall be distributed into two polypropylene tubes, in which 11% acetic acid (50 µL) was added beforehand (volume of acetic acid may be adjusted depending on obtained plasma volume), as follows:

- 0.5 mL (Primary) for MGS0008 pharmacokinetic analysis
- 0.5 mL (Backup) for MGS0008 pharmacokinetic analysis

All tubes shall be stored at or below -70ºC.

9.3.6.1.2 Shipping (MGS0008)

Shipment of Samples

Site staff personnel will send frozen samples under dry ice to the measurement institution shown below. The coordinating investigator will retain copies of shipment documents generated at the time of shipping. Shipment to the measurement facility will be carried out in two separate batches (primary and backup). All samples will be transported by World Courier with fax/email confirmation of receipt.

Shipping Address

Plasma samples for MGS0008 measurement will be shipped to:

[redacted]

9.3.6.2 Ketamine

Plasma samples will be analyzed to determine concentrations of ketamine. The determination of ketamine will be performed by the Analytical Psychopharmacology Laboratory at the Nathan S. Kline Institute (NKI).

Blood samples (10 mL) for determination of PK of ketamine will be collected on Final Screening Visit and Day 6, at 5 minutes after cessation of the MRI scan. The date and time of blood sampling must be recorded in the subject's study records. Instructions for preparation, storage and shipping of PK (ketamine) samples are found in the laboratory manual.

9.3.7 Pharmacogenomic (PGx) Blood Sampling

A single blood sample (8.5 mL) for PGx will be collected into a PAXgene™ Blood DNA tube for initial screening eligible subjects during Final Screening Visit. Only subjects who have consented to allow PGx research may have sample collected. The subject may choose to participate in the trial, but not participate in the PGx research. Instructions for preparation, storage and shipping of the PGx samples are found in the laboratory manual. The date of blood

sampling must be recorded in the subject's study records.

9.3.8 Blood Sample Volume for the Study

The total blood volume withdrawn from any single subject for all blood sampling during the course of the study is approximately 100 mL. If additional pharmacokinetic and/or safety analysis is necessary, additional blood may be obtained. The total blood volume withdrawn from any single subject will not exceed 150 mL.

9.4 Time Tolerances and Windows

Visit windows for each visit are summarized in Section 9.1. Time tolerances and windows, as defined below, are permitted for this study.

- PK sample collection for MGS0008 (3 h postdose of investigation drug): ± 5 minutes
- Ketamine infusion at Day 6 (8 h postdose of investigation drug): ± 2 hours
    - Relative time to ketamine infusion for MRI scan, safety monitoring (continuous ECG, pulse oximeter, BP monitoring), PK sample collection (ketamine) and post-ketamine behavioral assessments must be maintained
- MRI scan (25 minutes predose of ketamine at Final Screening visit and 7 h 35 min postdose of investigational drug on Day 6) start time: - 30 minutes (up to - 55 minutes of initiation of ketamine infusion)
- Post ketamine MRI scan end time (15 min postdose of ketamine at Final Screening visit and 8 h 15 min postdose of investigational drug at Day 6): + 3 minutes (up to 18 minutes in duration)
- PK sample collection for ketamine (20 min postdose of ketamine at Final Screening visit and 8 h 20 min postdose of investigational drug at Day 6): ± 5 minutes
- Behavioral assessments at Day 6 (pre-ketamine; 6 h postdose of investigation drug): between assessments at 3h postdose and initiation of MRI scan
- Behavioral assessments at Final Screening Visit (pre-ketamine; - 2 h predose of ketamine): between "before behavioral assessments" and initiation of MRI scan
- C-SSRS at Day 6 (3 h postdose of investigational drug): between post administration of investigational product and initiation of behavioral assessments
- Pre-infusion assessments at Day 6 (Body weight, physical examination [including a neurological examination], vital signs and 12-lead ECG): between post administration of investigational product on Day 5 and initiation of Day 6 ketamine infusion

Any deviation found from the planned procedure time (outside the window noted above) must be documented and explained in the site source document by the site staff.

10 STATISTICAL METHODS

In addition to this section of the study protocol, a more detailed statistical analysis plan (SAP) will also be provided in a separate document. If circumstances arise during the study that make these analyses inappropriate or if improved methods become available, the SAP will take precedence and may be revised, as needed. Any revisions (both alternative and additional methods) to the SAP that are used in the final report, and reasons for such revisions, will be described in the final report.

10.1 Sample Size Determination

The evaluable sample size was selected based on sample sizes of available similar ketamine studies.

10.2 Analysis Populations

The PD analysis population will include all randomized subjects who received correct dosing and those with at least 1 baseline and with sufficient post-baseline PD data. Missing data will not be imputed and are to be excluded from the analysis.

The safety analysis population will include all subjects with at least one dose of study medication.

The PK analysis population will include all dosed subjects with evaluable MGS0008 PK data and may exclude subjects if their data are considered invalid due to relevant missing values, or if any other problem occurred during sampling, laboratory analysis, dosing or AEs which invalidates the concentration measurements.

10.3 General Analysis Conventions

The scope of most analyses will be primarily descriptive. For continuous measures, data will be summarized by means, standard deviations, medians, minimum, and maximum. Where appropriate, change from baseline will also be summarized. For categorical measures, data will be summarized with frequency counts and percentages. All data analyzed will be displayed in detailed listings.

10.4 Demographic and Baseline Characteristics

Demographics and baseline characteristics including but not limited to age, gender, body weight, height, BMI, BOLD fMRI data, BPRS and CADSS scores will be tabulated by group using descriptive statistics.

10.5 Pharmacodynamics (PD) Analysis

10.5.1 Primary Endpoint

To evaluate the treatment effect of TS-134 on ketamine-induced BOLD signals in pre-specified ROIs (ACC and anterior insula) in resting fMRI, analysis of covariance (ANCOVA) with change from baseline at Day 6 BOLD as the outcome predicted by baseline (Final Screening Visit) BOLD and treatment in 3-treatment groups (TS-134 20 mg group, TS-134 60 mg group and placebo group) will be performed, and the mean and 95% confidence interval (CI) will be estimated. Separate ANCOVAs will be fit to each ROI. Tests of treatment effect will be made within each treatment group, between each active treatment group (TS-134 20 mg group and TS-134 60 mg group) versus placebo group, and also between the two active treatment groups from the ANCOVA. A statistically significant negative change of BOLD found in the active treatment groups compared to placebo group would be consistent with the hypothesis. Cohen's d effect sizes will be reported using the standard deviation of baseline BOLD.

10.5.2 Secondary Endpoint

To evaluate the treatment effect of TS-134 on ketamine-induced BOLD signals in whole brain in resting fMRI, ANCOVA similar to analysis for the primary endpoint will be performed.

To evaluate the treatment effect of TS-134 on clinical measures of BPRS and CADSS, ANCOVA similar to analysis for the primary endpoint will be performed.

10.5.3 Interim Data Transfer

Interim data transfer for change in BOLD signals in pre-specified ROIs in resting fMRI will be conducted for every 12 first and second ketamine session sets of assessments (i.e. following completion of 12th, 24th, 36th, 48 h, and 60th subjects). Each interim analysis result will be provided to the sponsor within 7 days following the completion of BOLD assessments of respective cut-off subjects.

10.6 Safety Analysis

Safety will be assessed for each group based on reported AEs, vital signs measurements, 12-lead ECGs, C-SSRS and clinical laboratory assessment results.

10.6.1 Adverse Events

Treatment emergent adverse events (TEAEs) will be listed by lower level terms and summarized by system organ class with preferred term based on the MedDRA reporting system. The number and percentage of subjects who experience one or more TEAEs will be tabulated. The tabulation will be further classified by severity and relationship to investigational product.

AEs reported from the time of initial informed consent through the initial administration of an investigational product will be listed in a separate listing from TEAEs.

10.6.2 Laboratory Values

Laboratory data from each sampling and changes from baseline will be summarized by the mean, median, standard deviation, minimum, and maximum. Laboratory analyses will utilize Day -1 (Admission) assessments as baseline values (preferably drawn under fasting conditions).

Parameters will be categorized as low, normal, or high according to the laboratory normal range specifications. Shift tables that show the change in laboratory values from baseline to Discharge (Day 7) will be constructed.

Clinical laboratory outlier criteria will be used to identify values and trends of potential medical importance. A summary for the number and percentage of subjects with clinical laboratory outliers for each specified laboratory test will be presented.

Criteria for clinical laboratory outliers will be described in the SAP.

10.6.3 Vital Signs

Vital signs will be summarized by mean, median, standard deviation, minimum and maximum at each measured time point. Vital signs analyses will utilize Day -1 (Admission) assessments as baseline values.

10.6.4 Electrocardiogram

Characteristics of cardiac events at each reading and changes from baseline (Day -1) will be summarized descriptively. Means, standard deviations, median, minimum and maximum of interval data (ventricular rate, RR, PR, QRS, and QTc) will be calculated for each group. The incidence of abnormalities, based on the clinical interpretations from the investigator, will be enumerated.

QTc as corrected by the Fridericia's method will be used as the primary measure of change in QT interval. Changes from baseline for each of the ECG parameters will be summarized using descriptive statistics. Borderline and prolonged QTc will be listed and frequency table provided. Additionally, the numbers of subjects with change from baseline in QTc at appropriate timepoints categorized as ≤ 30 msec, > 30 to 60 msec, and >60 msec, will be tabulated and summarized by treatment group as will the number of subjects with a QTc interval > 500 msec.

10.6.5 C-SSRS

Any suicidal ideation or behavior on the C-SSRS that develops during the study will be listed. Further details will be provided in the SAP.

10.7 Pharmacokinetics (PK)

10.7.1 MGS0008

The plasma concentration of MGS0008, final dose of TS-134, the last dosing date and time, and actual sampling date and time will be listed for each subject.

Further details will be provided in the SAP.

10.7.2 Ketamine

The plasma concentration of ketamine, the date and time of initiation of ketamine infusion, and actual sampling date and time will be listed for each subject.

Further details will be provided in the SAP.

10.8 Pharmacogenomic (PGx) Analysis

If safety or PK results warrant further investigation of genomic variation postulated to be involved in the metabolism, toxicity, or pharmacological response to TS-134 and/or ketamine, the sponsor or sponsor's designee may conduct genetic and other biomedical research on specimens, including genetic analyses (DNA). PGx samples will be stored under frozen conditions for up to 15 years and may be evaluated as information is gained on the PK, PD or AEs associated with TS-134 and/or ketamine.

11 ADVERSE EVENT REPORTING

11.1 Definitions, Grading & Relationship, Outcome and Follow-up

11.1.1 Definitions

Adverse Event (AE): Any untoward medical occurrence in a subject from the time of initial informed consent. An AE can be any unfavorable or unintended sign (including an abnormal finding), symptom, or disease temporally associated with the use of a medicinal (investigational) product. AEs include any occurrence that is new in onset or aggravated in severity or frequency from the baseline condition, or abnormal results of diagnostic procedures (including clinically-significant laboratory test abnormalities).

Treatment Emergent Adverse Event (TEAE): Any untoward medical occurrence in a subject from the time of initial administration of investigational product, part of AE.

Any AEs occurring from the time of the subject's first informed consent through the final study procedure shall be reported, whether or not the events are deemed to be related to the study product. Adverse incidents that occur before the subject's first informed consent are considered pre-existing conditions and will be recorded as medical history findings.

Events should be considered AEs if they:

- result in discontinuation from the study,
- require treatment or any other therapeutic intervention,
- require further diagnostic evaluation (excluding a repetition of the same procedure to confirm the abnormality),
- are associated with clinical signs or symptoms judged by the investigator to have a significant clinical impact.

Appropriate therapeutic action and follow-up measures will be performed by the investigator in accordance with GCP. These actions and measures will continue until the condition is resolved and/or the etiology is identified. Any non-serious AEs (including laboratory results or other clinical findings) that result in the subject's withdrawal from the study will require the subject to undergo early discontinuation procedures. The Medical Monitor must be informed of withdrawals due to AEs and the length of follow-up will be agreed upon between the Medical Monitor and the investigator at the site.

Laboratory Abnormalities: It is the responsibility of the investigator to review all laboratory findings in all subjects. Abnormal values should be commented upon as to clinical relevance or importance in the study records or the laboratory report as appropriate. An abnormal laboratory value may be considered an AE if the identified laboratory abnormality leads to any type of

intervention whether prescribed in the protocol or not. Medical and scientific judgment should be exercised in deciding whether an isolated laboratory abnormality should be classified as an AE.

All laboratory abnormalities considered to constitute an AE should be reported on the appropriate section of the subject's study records. Laboratory abnormalities do not need to be listed as separate AEs if they are considered to be part of a clinical syndrome that is being reported as an AE.

11.1.2 Grading and Relationship

The investigator will evaluate all AEs as to their severity, and record the outcome and action taken. The investigator will also judge the likelihood that the AE was related to the investigational product and document this in the subject's study records. See Table 11-1 below:

Table 11-1. Classification of Adverse Events

**Severity**	**Mild**	awareness of sign or symptom, but easily tolerated
	**Moderate**	discomfort enough to cause interference with usual activity
	**Severe**	incapacitating with inability to work or do usual activity
**Duration**	Record the start and stop dates of the adverse experience. If less than 1 day, indicate the appropriate length of time and units	
**Action taken**	Did the adverse experience cause the test drug to be discontinued? Interrupted? Dosage increased or decreased? Other action?	
**Relationship to test drug**	Did the test drug cause the adverse experience? The determination of the likelihood that the test drug caused the adverse experience will be provided by an investigator. The investigator's signed/dated initials on the source document supporting the causality noted on the AE form ensures that a medically qualified assessment was done. This initialed document must be retained for the required regulatory time frame. The criteria below are intended as reference guidelines to assist the investigator in assessing the likelihood of a relationship between the test drug and the adverse experience based upon the available information. The greater the correlation with the components and their respective elements (in number and/or intensity), the more likely the test drug caused the adverse experience (AE):	
	**Exposure**	Is there evidence that the subject/patient was actually exposed to the test drug such as: reliable history, acceptable compliance assessment (pill count, diary, etc.), expected pharmacologic effect, or measurement of drug/metabolite in bodily specimen?
	**Time Course**	Did the AE follow in a reasonable temporal sequence from administration of the test drug? Is the time of onset of the AE compatible with a drug-induced effect?
	**Likely Cause**	Is the AE not reasonably explained by another etiology such as underlying disease, other drug(s)/vaccine(s), or other host or environmental factors?
	**Dechallenge**	Was the dose of test drug discontinued or reduced?<br>If yes, did the AE resolve or improve?<br>If yes, this is a positive dechallenge. If no, this is a negative dechallenge.<br>**(Note: This criterion is not applicable if: (1) the AE resulted in death or permanent disability; (2) the AE resolved/improved despite continuation of the test drug; or (3) the study is a single-dose drug study.)**
	**Rechallenge**	Was the subject/patient reexposed to the test drug in this study?<br>If yes, did the AE recur or worsen?<br>If yes, this is a positive rechallenge. If no, this is a negative rechallenge.<br>**(Note: This criterion is not applicable if:**<br>**(1) the initial AE resulted in death or permanent disability, or (2) the study is a single-dose drug study.)**
	**Consistency with Investigational Product Profile**	Is the clinical/pathological presentation of the AE consistent with previous knowledge regarding the test drug or drug class pharmacology or toxicology?
	The assessment of relationship will be reported on the case report forms/worksheets by an investigator according to his/her best clinical judgment, including consideration of the above elements.<br>**Use the following scale of criteria as guidance (not all criteria must be present to be indicative of a drug relationship).**	
	**Definitely related**	A clinical event, including laboratory test abnormality, occurring in a plausible time relationship to drug administration, and which cannot be explained by concurrent disease or other drugs or chemicals. The response to withdrawal of the drug (dechallenge) should be clinically plausible. The event must be definitive pharmacologically or phenomenologically, using a satisfactory rechallenge procedure if necessary.

	**Probably related**	A clinical event, including laboratory test abnormality, with a reasonable time sequence to administration of the drug, unlikely to be attributed to concurrent disease or other drugs or chemicals, and which follows a clinically reasonable response on withdrawal (dechallenge). Rechallenge information is not required to fulfil this definition.
	**Possibly related**	A clinical event, including laboratory test abnormality, with a reasonable time sequence to administration of the drug, but which could also be explained by concurrent disease or other drugs or chemicals. Information on drug withdrawal may be lacking or unclear.
	**Not related**	A clinical event, including laboratory test abnormality, with a temporal relationship to drug administration which makes a causal relationship improbable, and in which other drugs, chemicals or underlying disease provide plausible explanations.

11.1.3 Outcome

The seriousness of an event is determined by its outcome (e.g., hospitalization, death, etc.). The action taken to treat an AE and the outcome of the action must be recorded. Outcomes may be categorized as shown below:

- FATAL: The termination of life as a result of an adverse event. Death Related to Adverse Event
- NOT RESOLVED: One of the possible results of an adverse event outcome that indicates that the event has not improved or recuperated.
- RESOLVED: One of the possible results of an adverse event outcome that indicates that the event has improved or recuperated.
- RESOLVED WITH SEQUELAE: One of the possible results of an adverse event outcome where the subject recuperated but retained pathological conditions resulting from the prior disease or injury.
- RESOLVING: One of the possible results of an adverse event outcome that indicates that the event is improving.
- UNKNOWN: Not known, not observed, not recorded, or refused.

11.1.4 AE Follow-Up

Any AEs that are ongoing after Day 14 should be followed for 30 days. Information collected after Day 14, however, will not be captured in the database, but will be maintained in the subject's study records and made available upon request.

11.2 Serious Adverse Event Reporting

11.2.1 Definition of Serious Adverse Event

A serious adverse event (SAE) is an untoward medical occurrence that:

- results in death (**Note**: Death is an outcome and not an event and the cause of death should be listed as the SAE);
- is life-threatening (i.e., the subject was at immediate risk of death from the AE);
- requires inpatient hospitalization or prolongation of existing hospitalization;
- results in persistent or significant disability/ incapacity; or
- is a congenital anomaly or birth defect (in the child of a subject who was exposed to the investigational product)

Any other important medical event that may result in death, be life-threatening, or require hospitalization, may be considered a SAE when, based upon appropriate medical judgment, the event may jeopardize the subject and may require medical or surgical intervention to prevent one of the outcomes listed above.

11.2.2 Serious Adverse Event Reporting Procedure

If in the opinion of a study investigator the event meets the criteria of a SAE, the following procedures will be followed:

- Immediately upon becoming aware of the event, the investigator will report the SAE by fax/email directly to the sponsor as specified in the safety plan. The following documents will be forwarded to the sponsor within 24 hours of reporting the event:
    - SAE Report
    - Con Med CRF page
    - Medical History page
    - Any other applicable information
- The initial report of a SAE may be submitted by email. The investigator must provide the minimal information: i.e. the protocol number, subject number, date of SAE, SAE term, short description of the event, causality (if determined) and the reason why the event is categorized as serious.
- The investigator will also notify the IRB of the event within the time frame specified in the IRB's SOPs after becoming aware of the SAE. An initial report followed promptly by a complete report will be forwarded to the IRB, or in accordance with the IRB policy.
- The subject will be observed and monitored carefully until (1) the condition stabilizes and/or resolves; (2) its cause is identified; or (3) it is otherwise determined by the Medical Monitor and investigator. Follow-up information relating to the SAE must be submitted to the sponsor by email as soon as additional data related to the event are available.
- If a subject is hospitalized or hospitalization is prolonged due to the SAE, the hospital discharge summary should be obtained if possible when it becomes available.
- If a death occurs and an autopsy is performed, a copy of the autopsy report should be obtained if possible when it becomes available. All efforts must be undertaken to obtain follow-up information promptly.

Any SAEs occurring from time of informed consent through 30 days after last dose

administration must be reported. All SAEs will be reported, whether or not they are deemed to be related to study product. Timely reporting of SAEs will be followed in accordance with regulatory requirements as outlined in the safety plan.

11.2.3 SAE Follow-Up

If a SAE is reported while the event is still ongoing, the investigator must submit follow-up reports to the sponsor regarding the subject's subsequent course. All SAEs, including those that are ongoing at the end of the study or upon discontinuation must be followed until resolution or stabilization.

11.3 Pregnancy Reporting

If a subject does become pregnant during the course of the study, the subject should be discontinued immediately. The site must report all pregnancies to the sponsor via email or telephone within 24 hours of receiving the report of pregnancy. The site should record and maintain all relevant information on the appropriate study form, including the follow-up and outcome of the pregnancy.

Pregnancy itself is not regarded as an AE/ SAE. Congenital abnormalities/birth defects and spontaneous miscarriages should be reported and handled as SAEs. Elective abortions without complications should not be handled as AEs. The outcome of all pregnancies (spontaneous miscarriage, elective termination, ectopic pregnancy, normal birth or congenital abnormality) should be followed up and documented even if the subject was discontinued from the study (with the subject's consent).

Male subjects should refrain from fathering a child or donating sperm during the study and for at least 90 days following the last dose. Pregnancy of the subject's partners is not considered to be an AE, however, the outcome of all pregnancies should be followed up and documented if possible.

12 KNOWN AND POTENTIAL RISKS AND BENEFITS TO HUMAN SUBJECTS

12.1 Benefits to Human Subjects

This study enrolls healthy volunteers and there are no direct benefits for subjects from taking part in this study.

[redacted]

12.3 Risks Associated with Ketamine Administration

Ketamine is a FDA-approved dissociative anesthetic. Ketamine exposure at sub-anesthetic doses can be associated with a moderate dissociative state, which is well tolerated in the majority of cases and spontaneously reversible[24]. There is extensive clinical experience with ketamine used at anesthetic doses, and no long-term detrimental effects of ketamine exposure have been reported. Administration of sub-anesthetic doses of ketamine may induce increase in blood pressure and pulse rate, nausea and vomiting[25, 26]. Ketamine administration may increase the risk of psychosis, even in healthy subjects. Ketamine is also known as a street drug of abuse, as such, its use may predispose subjects to subsequent abuse of this drug.

This study will minimize above risks of ketamine by imposing an upper limit of ketamine dosage per ketamine session (20 mg), by excluding participants with a history of cardiovascular disease, recreational ketamine or PCP use, or no more than 5 previous ketamine infusions, by evaluating vital signs and 12-Lead ECG at Screening Visits and Day -1 to exclude possible

hypertension subjects, by monitoring ECG, pulse and blood pressure during ketamine administration in the presence of a physician.

12.4 Risks Associated with MRI Scanning

3.0T MRI scanners used in this study satisfy FDA criteria for nonsignificant risk in all risk categories. The study will follow guidelines set by the FDA with regard to specific absorption ratio (SAR), limits on gradient slew rate (dB/dt), and noise. Subjects can experience acoustic noise associated with MRI, and in some instances, physical discomfort, stimulation of peripheral nerves (twitching or tingling, in very rare cases, painful) as a result of time-varying magnetic fields, some heating of body tissues due to absorption of radiofrequency energy. The risk of MRI to the fetus is unknown.

This study will minimize above risks of MRI scanning by taking following steps

- **Acoustic noise:** As suggested by the FDA, this study will take steps to reduce the noise levels experienced by subjects. The easiest and most reliable means of preventing hearing loss is to use disposable earplugs, which will be used for all scans. Acoustically shielded headsets will also be used for attenuating further noise.
- **Physical discomfort:** All subjects will be able to communicate directly with technologist and study staff to inform them of any emotional or physical distress during the scanning procedure. If they wish, the scan will be terminated immediately and the subject will be removed from the scanner.
- **Fetal exposure:** While there is no known risk of MR scans to the fetus, the study will exclude women who are pregnant, intend to become pregnant (within 90 days of the last dose), or are breastfeeding by investigator’s assessments of inclusion/exclusion criteria as well as blood pregnancy test at Initial Screening Visit and urine pregnancy test at Final Screening Visit and Day -1.
- **Static magnetic field:** This study will use the MRI facilities having a range of SOPs to assure security of the restricted access area, careful metal screening of subjects before they enter the restricted access area and a metal detector positioned at the doorway leading into the magnet room within the MRI suite.
- **Nerve Stimulation:** A record of dB/dt value will be included with the imagining data to help in analysis of levels of peripheral nerve stimulation possibly perceived by subjects. In addition, detailed calculations of the changes in magnetic field over time that our gradient system is capable of, and conservative values will be selected as limits that will be used to determine when special additional monitoring is indicated. In these cases, the monitoring procedures recommended by the FDA will be conducted. The gradient switching times and strengths will also be monitored together with the routine assessment of all electrical components of the system.

In addition, MR technologists receive special training to prevent peripheral nerve stimulation. Before any scanning procedure that might stimulate peripheral nerves, technologist will inform the subject that peripheral nerve stimulation may occur; describe the nature of the sensation to the subjects; instruct subjects not to clasp their hand, since this may create a conductive loop which will increase the possibility of stimulation; maintain constant verbal contact with the subject; instruct subjects to inform the MR technologist if they experience discomfort of pain; terminate the scan if the subject complains of discomfort or pain; complete a report of any incidents involving severe discomfort or pain, including describing the associated circumstances (imaging parameters, dB/dt value, level of pain, etc.), and submit this report immediately to the IRB.

- **SAR absorption:** The magnitude of temperature increase during MRI scanning is minimal. Increases are always within in FDA guidelines, which include core temperature increases less than 1°C, as well as localized heating to less than 38°C in the head, 39°C in the trunk, and 40°C in the extremities. 3.0T MRI to be used for this study will have in place a means to monitor RF power levels and ensure that energy deposition in sufficiently low to stay well within these guidelines for temperature increases. First, a “system security” unit is employed to integrate the output of the RF amplifies. If security detects an output that might exceed the guidelines noted above, it automatically shuts down the entire RF power system. Secondly, all pulse sequences are evaluated, based on calculations and sound scientific measurements, to ensure that SAR remains within FDA-approved guidelines, prior to their use in humans. Any experiment performed on the 3.0 Tesla system will comply with all FDA guidelines with regard to RF power deposition. Proper and routing monitoring of all RF electronics (e.g., coils, transmitters, system security, etc.) will be performed on a regular basis. All pulse sequences will be evaluated (by calculation and by valid scientific measurement) prior to use in human.

13 INVESTIGATOR OBLIGATIONS

This study will be conducted in accordance with GCP, Title 21 of the CFR, Part 50, Subparts A and B; Part 56; and Part 312, Subpart D; and the Consolidated Guidance for Industry GCP E6, April 1999; and 1996 ICH GCP E6.

13.1 Ethical Considerations

The investigator will ensure that the study is conducted in full conformance with the FDA standards for human research as specified in 21 CFR 312, Part D (Responsibilities of Sponsors and Investigators) and in accordance with the Declaration of Helsinki.

13.2 Institutional Review Board (IRB) Approval

Prior to initiating the study and receiving study medication, the investigator must obtain written approval to conduct the study from the appropriate IRB. The investigator must have signed the protocol signature page. When changes to the study protocol become necessary, protocol amendments will be submitted in writing by the investigator to the IRB for approval prior to implementation. Protocol administrative changes will be submitted in writing by the investigator to the IRB for review and notification. The IRB must be informed of all serious and unexpected AEs occurring during the study that are likely to affect the safety of the subjects or the conduct of the study.

In addition to the IRB approval, other documentation including FDA Form 1572, financial disclosure forms, investigator CV, applicable licensure for investigators and sub-investigators, a copy of the IRB approval letter, and an IRB approved consent form must be on file with Taisho Pharmaceutical R&D Inc. before an institution may enter subjects.

13.3 Informed Consent

All potentially eligible subjects for the study will be given a copy of the study ICF to read. All study subjects must sign this ICF if he/she decides to participate in the study. The investigational products will not be released to the subject who has not signed the ICF. The investigator or designee will inform all subjects as to the nature, aims, duration, potential hazards, and procedures to be performed during the study and that his or her medical records may be reviewed. The investigator will explain all aspects of the study in lay language and answer all the subject's questions regarding the study. The investigator must also explain that the subjects are completely free to refuse to enter the study or to withdraw from it at any time. All revisions of the protocol must be reflected in the ICF and reviewed by the IRB. Subjects who refuse to participate or who withdraw from the study will be treated without prejudice.

13.3.1 Health Insurance Portability and Accountability (HIPAA) Authorization

The Health Insurance Portability and Accountability Act of 1996 (HIPAA) contains provisions to protect the confidentiality and security of personally-identifiable information that arises in the course of providing health care. In order to understand how HIPAA affects research, there are a few important terms that are defined by the law. A covered entity is the organization that has to comply with HIPAA. Such organizations may be a Hybrid Covered Entity because, in addition to providing health care at medical facilities, it also has other organizational activities such as education and research.

The HIPAA Privacy Rule governs Protected Health Information (PHI) which is defined as information that can be linked to a particular person (i.e. is person-identifiable) that arises in the course of providing a health care service.

When PHI is communicated inside of a covered entity, this is called a use of the information. When PHI is communicated to another person or organization that is not part of the covered entity, this is called a disclosure. HIPAA allows both use and disclosure of PHI for research purposes, but such uses and disclosures have to follow HIPAA guidance and have to be part of a research plan that is reviewed and approved by an IRB. An institutionally approved HIPAA authorization must be completed for each subject. The Authorization can be included as part of the main study informed consent, but must have a second signature section in addition to signature section of the study informed consent. The HIPAA authorization will include the following attributes consistent with local privacy requirements:

13.3.1.1 Authorization Core Elements

- A description of the PHI to be used or disclosed, identifying the information in a specific and meaningful manner
- The names or other specific identification of the person or persons (or class of persons) authorized to make the requested use or disclosure
- The names or other specific identification of the person or persons (or class of persons) to whom the covered entity may make the requested use or disclosure
- A description of each purpose of the requested use or disclosure
- Authorization expiration date or expiration event that relates to the individual or to the purpose of the use or disclosure ("end of the research study" or "none" are permissible for research, including for the creation and maintenance of a research database or repository)
- Signature of the individual and date. If the individual's legally authorized representative signs the Authorization, a description of the representative's authority to act for the individual must also be provided

13.3.1.2 Authorization Required Statements

- A statement of the individual's right to revoke Authorization and how to do so, and, if applicable, the exceptions to the right to revoke Authorization or reference to the corresponding section of the covered entity's notice of privacy practices
- Whether treatment, payment, enrollment, or eligibility of benefits can be conditioned on Authorization, including research-related treatment and consequences of refusing to sign the Authorization, if applicable
- A statement of the potential risk that PHI will be re-disclosed by the recipient and no longer protected by the Privacy Rule. This may be a general statement that the Privacy Rule may no longer protect health information disclosed to the recipient

13.4 Subject Confidentiality

All reports and subject samples will be identified only by a coded number to maintain subject confidentiality. All records will be kept confidential to the extent permitted by law. The investigator should keep a separate log of subjects, codes, names and addresses. Documents which identify the subject by name (informed consent) should be kept in strict confidence.

Taisho Pharmaceutical R&D Inc. and its vendors agree to keep all subject information confidential. Only coded, blinded data will be released. Data resulting from analysis will be entered into a database that is not accessible to the public. Subject data will be identified only by the subject (randomization) number, and not by other annotation or identifying information. Taisho Pharmaceutical R&D Inc. and its vendors will take every possible step to reduce the risk of releasing information to its customers that would enable the customer to personally identify subjects.

13.5 Subject's Financial Responsibilities During the Study

Subjects will not be financially responsible for any procedures or tests of any investigational nature.

14 DOCUMENTATION, RECORD KEEPING, AND DATA MANAGEMENT

14.1 Source Data/Documents

Source data is defined as all information in original records and certified copies of original records of clinical findings, observations, or other activities in a clinical trial necessary for the reconstruction and evaluation of the trial. Source document is defined as the first place that data is captured/recorded. Any and all source data/documents must be maintained and be stored and/or retrievable at the site. Subjects' data recorded on or transferred to the CRF will be identified by a unique subject number. As an exception, if it is necessary to identify the subject for safety or regulatory reasons, Taisho Pharmaceutical R&D Inc. and the investigator are bound to keep this information confidential.

The investigator must maintain source data/documents for each subject in the study. All information appearing on the CRFs must be traceable to its source (paper or electronic), which are generally maintained in the subject's file/record. Source data should contain all demographic and medical information, including but not limited to:

- Laboratory data
- ECGs
- Vital signs assessments
- Physical exam findings
- SCID-5-CT results
- MRI results
- Behavioral assessment results
- C-SSRS results
- Prior and concomitant medications
- Signed original subject informed consent form (indicating the study number and title of the study)
- Pharmacy (investigational product) accountability/dispensation/destruction records
- Direct-entered electronic data from automated instrumentation
- Certified copies of original documents

Corrections or changes to data that are transcribed or transferred to the CRF must be traceable to source and must be initialed and dated by the (authorized) person making the changes. A reason for the change(s) or any discrepancies must be explained (i.e. audit trial).

14.2 Records Retention

Essential documents must be retained by the investigator for a period of at least two years after the last FDA marketing approval, or at least two years following the withdrawal of the NDA or cessation of investigational product development, or until written approval to destroy the documentation is provided by the sponsor. Taisho Pharmaceutical R&D Inc. will notify the investigator/institution in writing when the study-related records are no longer required. The investigator agrees to adhere to the document retention procedures by signing the protocol.

Essential documents include, but are not limited to:

- Study protocol and amendments (signed and IRB-approved)
- IRB composition/membership roster and/or Compliance Statement
- IRB correspondence/approvals
- IRB-approved advertising materials
- IRB-approved informed consent form
- CV of the principal investigator and sub-investigator(s)
- Investigator's Brochure
- Sample CRFs
- Monitoring visit logs
- Pre-study site evaluation report
- Subject screening (and enrollment) log(s)
- Delegation of Authority and/or Authorized Signatures log(s)
- Laboratory records (reference ranges, certifications, quality control documentation, and any other pertinent documents)
- Safety reports and/or Investigator Notifications of SAEs
- Quality control documentation
- Minutes of the safety review committees and documentation of changes to dosing (where applicable)
- Any other pertinent documents

The documentation must be retained longer, if so required by local law. Investigators must notify the sponsor, in writing, of changes in address, sales or practices or site closures in order to make arrangements for the maintenance of study files.

14.3 Data Management

All data management procedures will be detailed in a separate Data Management Plan (DMP).

15 CHANGES TO THE PROTOCOL AND STUDY TERMINATION

15.1 Study Design/ Procedure Modifications Permitted Within Protocol Parameters

This is a phase I exploratory study to assess TS-134 in humans; the safety and PD of TS-134 are still being explored. This protocol is written with some flexibility to accommodate the inherent dynamic nature of phase I exploratory clinical studies. Modifications to the dose level of TS-134 and to the PD procedures (MRI scan and behavioral assessments, including ketamine administration) that are currently outlined above may be required to achieve the scientific goals of the study objectives and/or to ensure appropriate safety monitoring of the study subjects. However any modifications that may increase the known safety risk on subjects will require protocol amendment and IRB approval with the protocol amendment.

As such, some alterations from the currently outlined procedures for dose level of TS-134, MRI scan, ketamine administration and behavioral assessments may be permitted based on newly available data, but the maximum daily dose of TS-134 (60 mg/day), the maximum ketamine dosage (20 mg/session), and total duration of MRI scan (40 minutes with additional 30 min as window allowance) will not exceed those currently outlined in the protocol. Below are examples of such alterations:

- Reduction of dose level of TS-134
- Adjustment of titration scheme of TS-134
- Adjustment of the ketamine administration
- Adjustment of the MRI scan duration
- Addition of safety monitoring procedures during MRI scan
- Adjustment of the timepoints of behavioral assessments.

The timing of procedures for assessment of safety parameters (e.g., vital signs, ECG, safety laboratory tests, etc.) currently outlined in the protocol may be modified during the study based on newly available safety, tolerability or PK data.

Assessment-specific exceptions to the time allowance windows noted in Section 9.4 may be granted for needed logistic flexibility if the scientific goals of the study will not be compromised, the needed schedule modification is documented and retained in study records, and written approval is granted by the sponsor.

It is understood that the current study may employ some or none of the alterations described above. Any alteration made to this protocol to meet the study objectives that do not increase subjects' safety risk must be detailed by the sponsor in a memo to the study file and forwarded to the investigator for review and retention. The memo may be forwarded to the IRB at the discretion of the investigator.

15.2 Protocol Amendment

If applicable, proposed dosing of investigational product, PD assessment procedures and other related assessment procedures may be modified based on PD and/or safety findings determined in the study on an ongoing basis, should the modification meet the scientific objective of the study and not increase safety risks for subjects. All changes to the protocol must be documented by amendments, or administrative changes where applicable, and the amended protocol must be signed by the sponsor and the investigator(s) and submitted for approval to the IRB. A copy of the approval will be provided to the site. Where the local IRB regulations regarding protocol amendments differ from this policy, the local regulations will apply.

15.3 Protocol Termination

The sponsor and the investigator reserve the right to terminate the study at any time. In terminating the study, the sponsor and the investigator will ensure that adequate consideration is given to the protection of each subject's interest.

16 STUDY MONITORING

16.1 Clinical Monitoring

An initiation meeting will be conducted by the sponsor or an authorized representative (e.g., clinical monitor). During this meeting, the protocol, CRFs, ICF and pertinent aspects of the US CFR will be reviewed with the investigator and all study staff.

Interim monitoring visits will be conducted by a clinical research associate (CRA) during the study at predefined intervals. Upon reasonable notice, the investigator and/or an authorized designee will set aside sufficient time to be available to the CRA to assist with the data query and resolution process, as well as to provide access to any additional records required for source data verification. During the course of the study, the responsible study staff will be available to discuss any matters relating to the conduct of the study.

At each site visit, the CRA will review CRFs and source documents to ensure that all items have been completed and that the data provided are accurate and obtained in the manner specified in the protocol. Incorrect, inappropriate, or illegible entries onto the CRFs will be returned to the

investigator and/or an authorized designee for correction. Monitoring visit agendas should also leave sufficient time to review the investigator site files for completeness and accuracy, and for investigational product inventories as appropriate.

Further details will be outlined in a clinical monitoring plan.

16.2 Auditing Procedures

A quality assurance audit may be performed by authorized representatives of Taisho Pharmaceutical R&D Inc., a regulatory agency or an IRB. If a regulatory authority requests an audit of the study site, the investigator is required to inform the sponsor immediately.

17 REFERENCES

[1] Regier DA, Narrow WE, Rae DS, et al. The de facto US mental and addictive disorders service system. Epidemiologic catchment area prospective 1-year prevalence rates of disorders and services. Arch Gen Psychiatry. 1993;50:85-94.

[2] Lieberman JA, Stroup TS, McEvoy JP, et al. Effectiveness of antipsychotic drugs in patients with chronic schizophrenia. N Engl J Med. 2005;353:1209-1223.

[3] Kew JN, Kemp JA. Ionotropic and metabotropic glutamate receptor structure and function. Psychopharmacology. 2005;179:4-29.

[4] Ohishi H, Shigemoto R, Nakanishi S, et al. Distribution of the messenger RNA for a metabotropic glutamate receptor, mGluR2, in the central nervous system of the rat. Neuroscience. 1993a;53:1009-1018.

[5] Ohishi H, Shigemoto R, Nakanishi S, et al. Distribution of the mRNA for a metabotropic glutamate receptor (mGluR3) in the rat brain: an in situ hybridization study. J Comp Neurol. 1993b;335:252-266.

[6] Schoepp DD, Wright RA, Levine LR, et al. LY354740, an mGlu2/3 receptor agonist as a novel approach to treat anxiety/stress. Stress. 2003;6:189-197.

[7] Swanson CJ, Bures M, Johnson MP, et al. Metabotropic glutamate receptors as novel targets for anxiety and stress disorders. Nat Rev Drug Discov. 2005;4:131-144.

[8] Cartmell J, Schoepp DD. Regulation of neurotransmitter release by metabotropic glutamate receptors. J Neurochem. 2000;75:889-907.

[9] Coyle JT. Glutamate and schizophrenia: beyond the dopamine hypothesis. Cell Mol Neurobiol. 2006;26:365-384.

[10] Moghaddam B, Adams BW. Reversal of phencyclidine effects by a group II metabotropic glutamate receptor agonist in rats. Science. 1998;281:1349-1352.

[11] Monn JA, Massey SM, Valli MJ, et al. Synthesis and metabotropic glutamate receptor activity of S-oxidized variants of (-)-4-amino-2-thiabicyclo-[3.1.0]hexane- 4,6-dicarboxylate: identification of potent, selective, and orally bioavailable agonists for mGlu2/3 receptors. J

Med Chem. 2007;50:233-240.

[12] Rorick-Kehn LM, Johnson BG, Burkey JL, et al. Pharmacological and pharmacokinetic properties of a structurally novel, potent, and selective metabotropic glutamate 2/3 receptor agonist: in vitro characterization of agonist (-)-(1R,4S,5S, 6S)-4-amino-2-sulfonylbicyclo[3.1.0]-hexane-4,6-dicarboxylic acid (LY404039). J Pharmacol Exp Ther. 2007;321:308-317.

[13] Krystal JH, Abi-Saab W, Perry E, et al. Preliminary evidence of attenuation of the disruptive effects of the NMDA glutamate receptor antagonist, ketamine, on working memory by pretreatment with the group II metabotropic glutamate receptor agonist, LY354740, in healthy human subjects. Psychopharmacology. 2005;179: 303-309.

[14] Millan MJ, Andrieux A, Bartzokis G, et al. Altering the course of schizophrenia: progress and perspectives. Nat Rev Drug Discov. 2016 Jul;15(7): 485-515.

[15] Stone JM, Dietrich C, Edden R, et al. Ketamine effects on brain GABA and glutamate levels with 1H-MRS: relationship to ketamine-induced psychopathology. Mol Psychiatry. 2012; 17 (7): 664-665.

[16] Moghaddam B, Adams B, Verma A, et al. Activation of glutamatergic neurotransmission by ketamine: a novel step in the pathway from NMDA receptor blockade to dopaminergic and cognitive disruptions associated with the prefrontal cortex. J Neurosci. 1997; 17 (8): 2921-2927

[17] Kim SY, Lee H, Kim HJ, et al. In vivo and ex vivo evidence for ketamine-induced hyperglutamatergic activity in the cerebral cortex of the rat: potential relevance to schizophrenia. NMR Biomed. 2011; 24 (10); 1235-1242.

[18] De Simoni S, Schwarz AJ, O'Daly OG, et al. Test-retest reliability of the BOLD pharmacological MRI response to ketamine in healthy volunteers. Neuroimage. 2013 Jan 1; 64: 75-90.

[19] Stone J, Kotoula V, Dietrich C, et al. Perceptual distortions and delusional thinking following ketamine administration are realted to increased pharmacological MRI signal changes in the parietal lobe. Journal of Psychopharmacology. 2015; Vol. 29(9) 1025-1028.

[20] Doyle OM, De Simoni S, Schwarz AJ, et al. Quantifying the attenuation of the ketamine pharmacological magnetic resonance imaging response in humans: a validation using antipsychotic and glutamatergic agents. J Pharmacol Exp Ther. 2013 Apr;345(1):151-60.

[21] Shellock FG. Guide to MR procedures and metallic objects. Lippincott Williams and Wilkins Healthcare, Philadelphia, 2001

[22] Overall JE, Gorham DR. The brief psychiatric rating scale. Psychol Reports. 1962; 10: 799-812.

[23] Bremner JD, Krystal JH, Putnam F, et al. Measurement of dissociative states with the clinician administered dissociative states scale (CADSS). J. Trauma. Stress. 1998; 11: 125-136.

[24] Krystal JH,Karper JP, Seibyl JP, et al. Subanesthetic effects of the noncompetitive NMDA antagonist, ketamine, in humans. Archives of General Psychiatry. 1994; 51(3):199-214.

[25] Kegeles LS, Abi-Dargham A, Zea-Ponce Y, et al. Modulation of amphetamine-induced striatal dopamine release by ketamine in humans: implications for schizophrenia. Biol Psychiatry. 2000; 48(7): 627-640.

[26] Kegeles LS, Mrtinez D, Kochan LD, et al. NMDA antagonist effects on striatal dopamine release: positron emission topography studies in humans. Synapse. 2002; 43(1): 19-29.
